# eHealth Technologies for Monitoring Pediatric Asthma at Home: Scoping Review

**DOI:** 10.2196/45896

**Published:** 2023-07-21

**Authors:** Mattiènne R van der Kamp, Vera S Hengeveld, Marjolein G J Brusse-Keizer, Boony J Thio, Monique Tabak

**Affiliations:** 1 Pediatric Department Medisch Spectrum Twente Enschede Netherlands; 2 Department of Biomedical Signals and Systems University of Twente Enschede Netherlands; 3 Medical School Twente Medisch Spectrum Twente Enschede Netherlands; 4 Health Technology and Services Research Faculty of Behavioral, Management and Social Sciences University of Twente Enschede Netherlands

**Keywords:** telemedicine, wearable electronic devices, asthma, child, pediatrics, internet-based interventions, monitoring, computers, hand-held device, medication, spirometry

## Abstract

**Background:**

eHealth monitoring technologies offer opportunities to more objectively assess symptoms when they appear in daily life. Asthma is the most common chronic disease in childhood with an episodic course, requiring close follow-up of pediatric asthma control to identify disease deterioration, prevent exacerbations, and enhance quality of life. eHealth technologies in pediatric asthma care show promising results regarding feasibility, acceptability, and asthma-related health outcomes. However, broad systematic evaluations of eHealth technologies in pediatric asthma are lacking.

**Objective:**

The objective of this scoping review was to identify the types and applications of eHealth technologies for monitoring and treatment in pediatric asthma and explore which monitoring domains show the most relevance or potential for future research.

**Methods:**

A scoping review was conducted using the PRISMA-ScR (Preferred Reporting Items for Systematic Reviews and Meta-Analyses extension for Scoping Reviews) guidelines. A systematic and comprehensive search was performed on English papers that investigated the development, validation, or application of eHealth technologies for home monitoring or treatment of pediatric asthma in the following databases: PubMed, Cochrane Library, IEEE, Scopus, CINAHL, PsycINFO, and ACM Digital Library. Two authors independently assessed eligibility and extracted data. Data were presented by a descriptive analysis of characteristics and a narrative report for each eHealth domain.

**Results:**

The review included 370 manuscripts. The following 10 monitoring domains were identified: air quality, airway inflammation markers, lung function, physical activity, sleep, audiovisual, other physiological measurements, questionnaires, medication monitoring, and digital environment (ie, digital platforms, applications, websites, and software tools to monitor or support monitoring). Rising numbers of studies were seen, and the numbers accelerated in the last few years throughout most domains, especially medication monitoring and digital environment. Limited studies (35/370, 9.5%) of multiparameter monitoring strategies, using three or more domains, were found. The number of monitoring validation studies remained stable, while development and intervention studies increased. Intervention outcomes seemed to indicate the noninferiority and potential superiority of eHealth monitoring in pediatric asthma.

**Conclusions:**

This systematic scoping review provides a unique overview of eHealth pediatric asthma monitoring studies, and it revealed that eHealth research takes place throughout different monitoring domains using different approaches. The outcomes of the review showed the potency for efficacy of most monitoring domains (especially the domains of medication monitoring, lung function, and digital environment). Future studies could focus on modifying potentially relevant hospital-based diagnostics for the home setting to investigate potential beneficial effects and focus on combining home-monitoring domains to facilitate multiparameter decision-making and personalized clinical decision support.

## Introduction

Asthma is the most common chronic childhood disease, affecting up to 10% of children worldwide [1]. Childhood asthma can lead to recurrent airflow limitation, which may hamper physical, psychological, and social development and well-being. The manifestation of asthma symptoms varies based on the asthma severity, the level of adequate disease management, and the influence of environmental triggers and the intrinsic waves of asthma, explaining its episodic fluctuating course. In clinical practice, assessment of asthma control is currently based on multiple diagnostic features (the combination of anamnesis, and physical examination and lung function measurements to assess airflow limitation). These scheduled elective outpatient clinic evaluations at infrequent intervals seem to conflict with the episodic nature of asthma, hampering timely and proper medical anticipation [2]. Closer follow-up of pediatric asthma control with a multifaceted assessment of disease parameters is thus needed to prevent disease deterioration, enhance self-management, and boost quality of life (QoL) [3,4].

eHealth technologies like wearable home-monitoring tools can longitudinally measure symptoms, risk factors, and treatment factors in daily life, outside elective visits. In combination with communication technologies, this type of eHealth technology provides opportunities to closely monitor asthma control at home and allows timely treatment as recommended by the GINA (Global Initiative for Asthma) guidelines [5]. To date, eHealth technology studies have reported on physiology monitoring (eg, lung function, respiratory rate, and nocturnal coughing), behavioral monitoring (eg, activity, therapy adherence, and trigger exposure), and self-management interventions (by education, health care provider support, etc), but they are often not specifically tailored to the pediatric population [6].

Studies applying eHealth technologies in pediatric asthma care have shown overall promising results regarding feasibility, acceptability, and asthma-related health outcomes [7-13]. For example, Ramsey et al [8] showed that digital interventions aimed at improving adherence resulted in improved adherence and asthma outcomes, and van den Wijngaart et al [14,15] showed that a digital asthma control test monitoring intervention reduced outpatient visits and was cost-effective. However, existing studies showed high heterogeneity in study endpoints, designs, and populations, which hampered systematic conclusions on the impact of eHealth in the management of pediatric asthma [11,12].

Clearly, studies on eHealth pediatric asthma monitoring and treatment are available, but they are very widespread and heterogeneous in terms of monitoring domains, applied methods, and evaluations. In order to be able to further identify future research directions, a broad overview of this research area is initially needed. We therefore conducted a scoping review of the available evidence for monitoring and treatment in pediatric eHealth, without being directed toward a single discrete monitoring domain or study design. Scoping reviews, combining analytic and narrative synthetization of evidence, have become increasingly common in the field of eHealth reviewing [16,17]. This method suits the fast-paced broad field of digital health in pediatric asthma, as it allows for the synthesis of a wide range of available evidence in the literature, the clarification of the key concepts of eHealth technology domains, and the identification of current knowledge gaps [18,19]. The objective of this review was to identify the types and applications of eHealth technologies for monitoring and treatment in pediatric asthma and to explore which monitoring domains show the most relevance or potential for future research.

## Methods

### Protocol and Registration

Our protocol was drafted using the Preferred Reporting Items for Systematic Reviews and Meta-Analyses extension for Scoping Reviews (PRISMA-ScR) guidelines [20].

### Eligibility Criteria

To be included in the review, papers needed to investigate the development, validation, or application of eHealth technologies for home monitoring and treatment in pediatric asthma. Peer-reviewed journal papers and conference papers were included if they were written in English. To avoid missing emerging technologies, quantitative, qualitative, and mixed method studies were included without restriction on study design, and study protocols were included as long as final study results had not been published. Papers were excluded if they did not fit into the conceptual framework of this scoping review as shown in Textbox 1.

**Inclusion and exclusion criteria of the scoping review.**

**Inclusion criteria**

Study goal

- Describes development and validation of the application of eHealth technologies for home monitoring of pediatric asthma.

Article type

- Peer-reviewed journal paper, protocol, or conference paper.

Study setting

- All clinical settings where children with asthma are treated: hospitals, outpatient clinics, public health/community clinics, or provider’s offices.

Language

- Written in English.

**Exclusion criteria**

Study goal

- No intention of longitudinal monitoring.

Article type

- In case of protocol studies, final study results are already published.

Study setting

- Community setting that is not at home (eg, school or recreational setting).
- Inclusion of both children and adults, and not explicitly specifying the results with children.

Language

- Written in a language other than English.

### Information Sources and Search

We searched PubMed, Cochrane Library, IEEE, Scopus, CINAHL, PsycINFO, and ACM Digital Library to identify potentially relevant documents until January 15, 2021. Since the primary aim of this scoping review was to provide an overview of developments within childhood asthma eHealth care, no lower limit for year of publication was set. The search strategies were drafted by the research team and refined by discussing them with an experienced librarian. The final search strategy for MEDLINE can be found in Multimedia Appendix 1. The final search results were exported to Rayyan, and duplicates were removed by 2 researchers.

### Selection of Sources of Evidence

To increase consistency among reviewers, both reviewers (MK and VSH) screened a random sample of 100 titles and abstracts (in chronological order), discussed the results, and amended the screening and data extraction manual before beginning screening for this review. The manual was refined to ensure that eHealth was used for monitoring or treatment purposes in all included articles. Two reviewers (MK and VSH) evaluated the titles and abstracts of all publications identified by our searches for potentially relevant publications. Articles not fulfilling the inclusion and exclusion criteria (Textbox 1) were excluded. Discrepancies were resolved by consensus and discussion between the 2 reviewers, and when disagreement persisted, the full-text article was analyzed to achieve consensus. eHealth domains were inductively constructed by 2 reviewers (MK and VSH) based on the title and abstract screening. The constructed eHealth domains were as follows: air quality, airway inflammation markers, lung function, activity, sleep, audiovisual, other physiological measurements, questionnaires, medication monitoring, and digital environment. Digital environment encompassed apps, websites, web portals, algorithms, and other digital tools that have the explicit goal to monitor asthma. This could be as a stand-alone tool (such as a digital diary and online communication to consult in times of symptoms) or could be supportive to monitoring tools from other eHealth domains (such as reminders and online asthma action plans). An overview of the constructed eHealth domains is provided in Table 1. One article could cover multiple eHealth domains. A second round of title and abstract screening was performed to label eligible articles with the applicable eHealth domains and classify them as “development/validation” or “intervention” studies. The full texts of eligible articles were then obtained and screened for eligibility based on the full texts by 1 reviewer (MK or VSH). Simultaneously, data extraction for eligible papers was performed, and the assigned eHealth domains and classification into “development/validation” or “intervention” were corrected when necessary. The full texts of 1 domain (airway inflammation markers) were assessed by both reviewers (MK and VSH) to ensure agreement on data extraction.

**Table 1:** Description per eHealth domain for pediatric asthma monitoring.

eHealth domain	Description
Air quality	Measures that reflect air quality, including air pollution, weather-related factors, and allergens
Airway inflammation markers	Markers that indicate airway inflammation
Lung function	Assessment of lung function, including peak expiratory flow and spirometry
Physical activity	Assessment of physical activity
Sleep	Assessment of sleep duration or quality
Audiovisual	Sound or video recordings to support asthma monitoring, such as recordings of cough, wheezing, or respiratory distress
Other physiological measurements	Physiological monitoring data that are not included in one of the other physiological monitoring domains
Questionnaires	Electronic questionnaires
Medication monitoring	Assessment of controller or reliever medication use, including inhaler adherence and inhaler technique
Digital environment	Digital platforms, applications, websites, and software tools that have the explicit goal to monitor asthma or are supportive to monitoring tools from other eHealth domains

### Data Extraction

A data extraction form was developed jointly by 2 reviewers (MK and VSH) in Google forms. We abstracted general data on article characteristics (eg, country of origin and year of publication), study design, and study population (eg, number of participants and age group), as well as the measurement methods used per domain and the main conclusions. For the domains of lung function and medication monitoring, we specifically extracted data on the evaluation of the spirometry technique and inhalation technique, respectively, at home. For papers describing or including an intervention, we additionally extracted who performed the intervention and the effects on (1) symptoms, (2) QoL, (3) lung function, (4) adherence, (5) self-management, (6) health care use and costs, and (7) school absence. Fitting the purpose of a scoping review, no critical appraisal of individual sources of evidence was performed.

### Synthesis of Results

Visual representations of publications on different domains over the years were made. We performed a descriptive analysis of the characteristics of the included papers for the year of publication and country of origin of all eHealth domains. Moreover, we analyzed the interactions between the monitoring domains. Thereafter, data were presented in a narrative format per eHealth domain.

## Results

### Search Results

**Figure 1:**
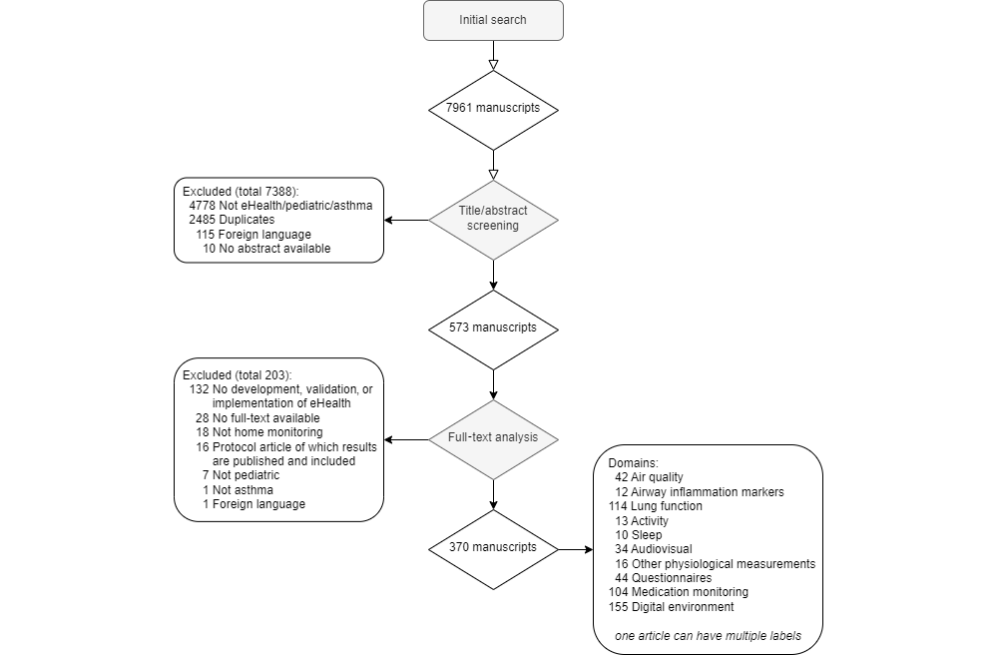
Flowchart of the selection of evidence sources.

The search resulted in 7961 records. The selection of sources of evidence is shown in the PRISMA-ScR flow diagram (Figure 1). All included studies with corresponding domain labels are shown in Multimedia Appendix 2 [3,7-15,21-378].

### Characteristics of the Sources of Evidence

**Figure 2:**
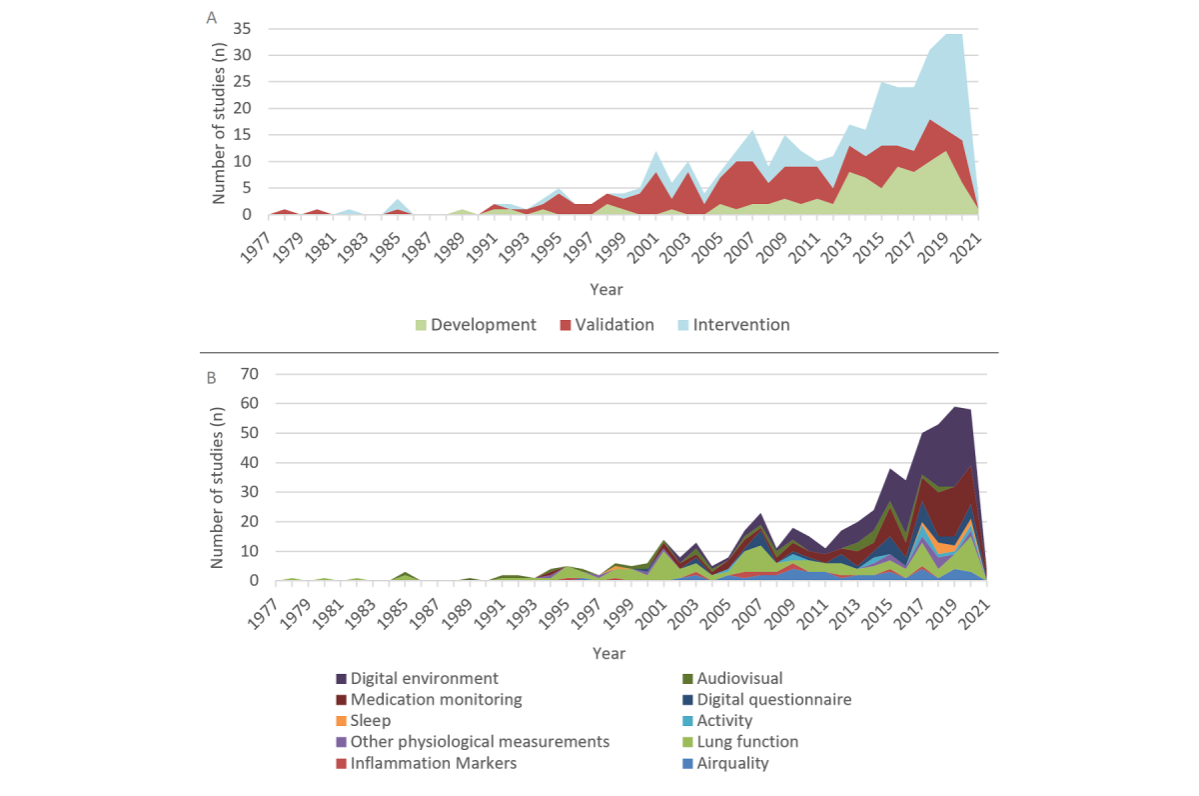
(A) Number of development, validation, and intervention studies per year. (B) Number of studies per eHealth domain per year.

Increasing numbers of studies regarding eHealth technologies for monitoring and treatment in pediatric asthma were found over time (Figure 2). From 1990 to 2014, the largest proportion of studies were validation studies evaluating the correlation of home-measured signals with asthma features. From 2014, increasing proportions of intervention studies were seen, with a share of 63% (24/38) in 2020.

With regard to the monitoring domains, an increasing variety of monitoring domains was seen over the years (Figure 2B). The first home studies in the 1980s focused on lung function monitoring. Moreover, several studies in the audiovisual domain followed in those years. From 1993, other domains started to sporadically appear in the pediatric asthma home-monitoring research field. For example, medication monitoring involved growing numbers of studies at every innovation step, with digital counters in 1990-2000, smart inhalers in 2000-2010, and the increased connectivity of smart inhalers with mobile devices in 2010-2020. Home monitoring of inflammation markers was an often researched topic from 2006, but this decreased after 2010. Air quality research showed a similar arrival period, but it remained quite stable after 2012 with regard to the absolute numbers of studies. Together with the rise of home computers, starting in 2008, a steady increase in studies in the digital questionnaire and digital environment domains was seen, and with the introduction of smartphones, the share of these domains grew enormously to over 40% of all home-monitoring studies. With the introduction of wearable technologies and smart watches, from 2015, an increase was seen in home-monitoring studies involving activity, sleep, and other physiological measurements.

**Figure 3:**
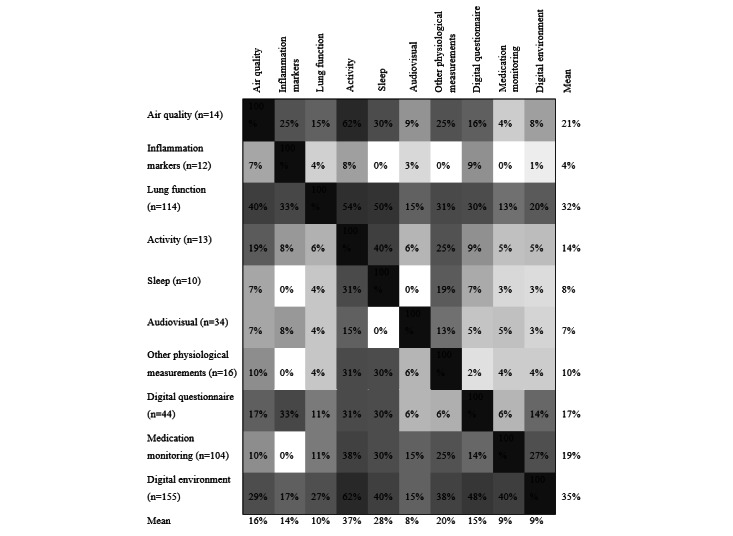
Overlap between domains. Each value in a column represents the percentage of articles falling under the domain of the row. Mean values were calculated for both the rows and columns (excluding the 100% diagonal). A darker color indicates a higher percentage.

In 30.8% (114/370) of the included studies, two or more monitoring domains were combined, of which the largest part (80/114, 70.2%) combined a digital environment with one or more other monitoring domains. Overall, 8.6% (32/370) and 3.5% (13/370) of studies used a multiparameter monitoring strategy combining three or more domains and four or more monitoring domains, respectively. Detailed domain interactions are visualized in Figure 3. These data show that the domains of activity and sleep were most frequently combined with another domain (average 37% and 28%, respectively), whereas the domain of audiovisual was least combined. Many domains were combined with the domain of lung function (average 32%) or digital environment (average 35%). However, within the domain of digital environment, there were relatively few interactions (average 9%), indicating many stand-alone digital environments as well (n=75).

**Figure 4:**
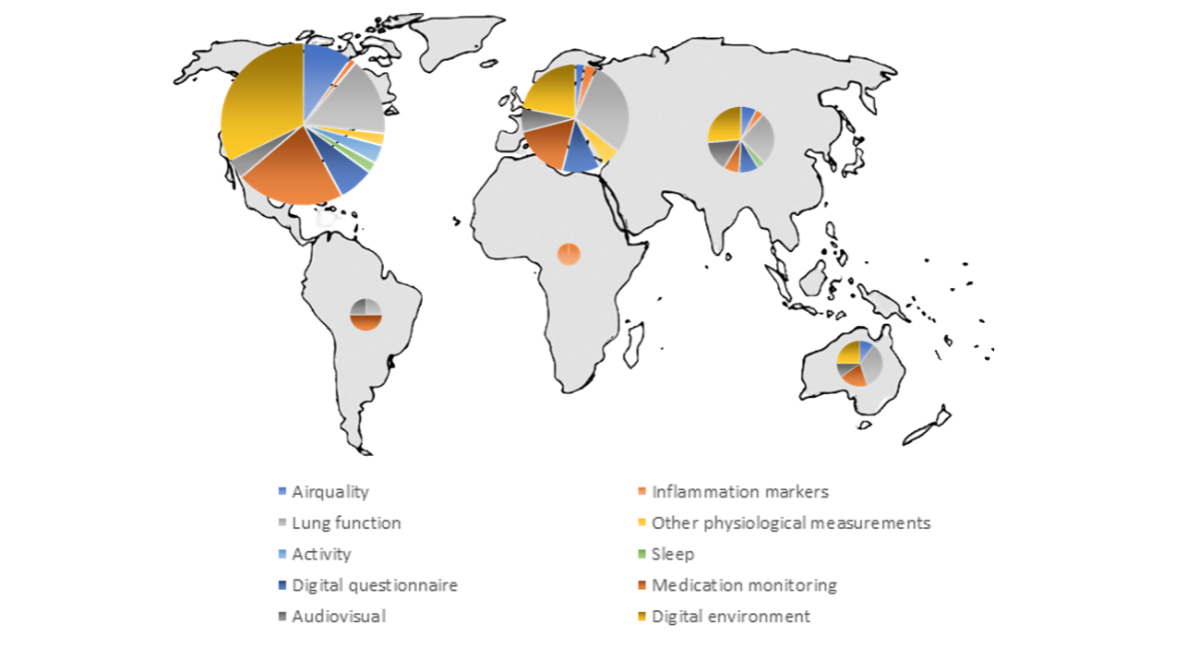
Visual representation of studies per eHealth domain per continent. The size of the circle indicates the total number of studies in the continent.

eHealth pediatric asthma research is performed throughout the world (Figure 4); however, the share of research from South America and Africa is limited (5/370, 1.4%) compared with the other continents. The 5 countries with the most studies were the United States (n=202), the Netherlands (n=41), Great Britain (n=31), Australia (n=13), and Taiwan (n=9). eHealth pediatric asthma research in North America mainly focused on the monitoring domains of digital environment (105/211, 49.8%) and medication monitoring (70/211, 33.2%), while lung function monitoring was the main monitoring domain in Europe (41/101, 40.6%). Moreover, Europe had a relatively large share of research focusing on questionnaire monitoring (16/101, 15.8%). Although the proportional share of sleep monitoring was the highest in Asia (2/35, 5.7%), the absolute count of activity and sleep monitoring studies in North America was overwhelmingly the largest (n=19) compared with the other continents (n=4). Home-based air quality research has a lower relative share in Europe (4/101, 4.0%) compared with Asia, North America, and Australia (>10%).

### eHealth Domains

The development, validation, and intervention results are reported below for each eHealth domain. No intervention studies were found for the domains of air quality, activity, sleep, and audiovisual.

#### Air Quality Domain

Forty-two studies were found, in which monitoring for air quality could roughly be divided into air pollution (PM2.5, O_3_, and NO_2_), weather-related factors (temperature and humidity), and allergy-triggering factors (pollen and dust), using a NO_2_ sensor, air pollution sensor, pollen tracker, or GPS tracker. Monitoring of air quality was based on (1) measurements in the bedroom [21,22], (2) measurements using data from weather stations adjusted to GPS tracking or mathematical models [23], or (3) measurements using a wearable monitor [24]. Wearable devices to monitor air quality were developed from 2017 onwards [21,23,25-29]. An example is the PIPER robot, which can move like a child and measures air quality in the home at a child’s height [30]. Some air quality devices were integrated in eHealth asthma tools to alert for bad air quality and provide feedback to the child [21,24,31-35].

Observational studies mostly focused on air pollution (29/32, 91%), with occasional combination with weather-related (8/32, 25%) or allergy-triggering (7/32, 22%) factors. There were significant negative effects of air pollution on children’s pulmonary health, especially for those with asthma [36]. Several studies found an association between increased air pollution and increased asthma symptoms [37-40] or lung function deficit [41,42]. There often was a delay between exposure and asthma symptoms (lag of 1-2 days) and a cumulative effect of exposure [43,44]. Studies on allergy-triggering and weather-related factors were limited [40,45]. Lawson et al [40] suggested that exposure to endotoxins may influence asthma symptoms or result in exacerbations. Li et al [45] showed an association between increased diurnal temperature range and reduced peak expiratory flow (PEF) and increased respiratory symptoms [45]. No intervention studies on air quality were found.

#### Airway Inflammation Markers Domain

Ten development/validation studies used fractional exhaled nitric oxide (FeNO) to measure airway inflammation. The oldest study addressed the potential of FeNO measurements for use as a home-monitoring tool [46]. Subsequently, Paredi et al [47] described a device that used a reservoir to allow delayed assessment of FeNO values and enabled home measurements. Thereafter, a hand-held electrochemical device, the NIOX-MINO, was developed and validated against standard FeNO measurements, with good between-method agreement within a clinically acceptable range [48,49]. Good feasibility and repeatability of FeNO home monitoring were reported [50-52].

In a proof-of-concept study, van der Valk et al [53] used the NIOX-MINO device for daily observation of FeNO in relation to asthma symptoms. They concluded that single FeNO values were not predictive to detect upcoming exacerbations, but that multiple data points were required.

##### Intervention Studies

One randomized controlled trial intervention study showed no added benefits of daily FeNO monitoring on symptoms, lung function, and airway inflammation in a telemonitoring program; however, the authors reported a tendency toward fewer exacerbations [52].

#### Lung Function Domain

Seventy-four development/validation articles were found that monitored lung function at home, and all used spirometric measurements, such as PEF and forced expiratory volume in 1 second (FEV1). Older articles mainly used hand-held peak flow devices, whereas more recent articles more often used hand-held spirometers. Sixteen studies combined spirometry with other home-monitoring devices, such as cough, sleep, and air quality sensors [21,22,31,34,36,40-45,51,54-58].

A good correlation of portable devices with hospital-based measurements was reported for both the PEF and FEV1 measurements [52,59-61], where home measurements tended to be lower (mean difference PEF: 22-55 L/min; FEV1: 0.02-0.15 L) [52,60,62]. The frequently incorrect, invented, or missing data in written diaries, with discrepancy between measured and self-reported values up to 35-50% [63-68], emphasize the need for electronic monitoring to prevent reporting bias [63,67,69,70].

There was little evidence evaluating the quality of execution of home-measured lung function. The quality of spirometry at home seemed acceptable and reproducible [71-74], even when compared with in-office spirometry [73,74]. Gamification methods were developed, aiming to enhance the quality and adherence of lung function measurements [75-77].

From the 1990s onwards, multiple studies reported on the limited value of PEF monitoring [78-80], as it was not a sensitive or specific objective index of lung function [81,82] and it poorly reflected changes in asthma activity [64,66,83-85]. When comparing PEF monitoring and spirometry, Sly et al [86] showed a moderate correlation between these. Two other validation studies using FEV1 for home-monitoring purposes showed a poor concordance of FEV1 with disease activity [84] and only small differences in FEV1 between symptom days and symptom-free days [85]. On the contrary, van der Kamp et al [3] described that variation in FEV1 distinguished between controlled and uncontrolled asthma.

##### Intervention Studies

Thirty-eight intervention studies on lung function monitoring were found. Within these intervention studies, lung function was commonly measured on standard intervals, with few studies reporting reversibility at home [87-90] or additional measurement of lung function when experiencing an increase in asthma symptoms [89-91]. Lung function monitoring was combined with symptom monitoring with a diary, questionnaire, or online contact in several studies [92-98]. Eleven studies took the quality of execution of spirometric maneuvers into account by providing feedback on the technique at more than one moment during clinical visits or by video assessment [88-90,92,95,99-103].

Patients and parents reported high rates of satisfaction with PEF monitoring [87,92,93,102], for example, to detect poor symptom perception, to assess bronchodilator response, or for reassurance in case of normal PEF values [87]. Despite known technical and logistical errors with PEF monitoring, acceptable compliance and feasibility were shown when introduced in a motivated group [90,104]. Multiple studies used a decision-support tool to adjust treatment based on home-monitored PEF values [97,100,105-107]. For example, Savva et al [105] showed a decrease in short-acting beta-agonist (SABA) use when SABA use was based on personal PEF values.

Myers et al [108] described the use of PEF monitoring in their review and concluded that the debate on the added value of PEF monitoring for asthma outcomes remains inconclusive. The reported advantages of PEF monitoring are increased self-management [98,109,110], increased trigger identification [111], and decreased emergency department contacts [92,110] and hospital admissions [92]. However, other studies reported no reduction in morbidity [112], QoL [88,92], or health care use [88], or improvement in symptom perception [88] when using PEF monitoring [112]. Moreover, Kamps et al [112] and Brouwer et al [113] concluded that PEF monitoring is not recommended in children with asthma on a routine basis, but can be helpful in some patients and can help to identify asthma triggers.

For interventions that include home spirometry monitoring, the results are also inconclusive, as some studies showed an improved QoL [94] and increased asthma control [96,114], whereas others showed no improvement in QoL [102] or the number of exacerbations [102]. Spirometry and PEF measurements at home are still an ongoing research topic, as several protocol studies included home monitoring of lung function to study the effects on perception, QoL, adherence, and health care use [115-117].

#### Physical Activity Domain

Our search yielded 13 studies. Accelerometry is a common method to objectively track physical activity and was used in all included studies. Accelerometry was often used complementary to measurements from other asthma monitoring domains [3,23,24,28,31-34,118,119]. For example, Rhee et al [118], Buonocore et al [31], and Hosseini et al [24] combined physical activity measurements with monitoring asthma symptoms, as physical activity is known to be a potential trigger for childhood asthma. Moreover, Fletcher et al [23] used activity levels to correct for increased exposure to air pollution due to an increased breathing frequency during activity.

Studies that investigated the correlation between physical activity levels and asthma symptoms revealed different relations. Some showed no correlation between activity levels and asthma symptoms [3,120], whereas others showed decreased physical activity levels in children with a history of wheezing in the last 12 months [121].

#### Sleep Domain

Nine articles were found for the sleep domain. Different methods of sleep assessment were used. The studies mainly used actigraphy [3,33,54,57,120,122], and some studies used ballistography [123] and a digital questionnaire [113,124]. Bian et al [120] used both actigraphy and a sleep questionnaire, and showed only a moderate correlation between the 2 approaches.

Most studies were observational studies [3,33,54,57,120,122-124] that investigated the relation between sleep and asthma. Sadeh et al [57] described a close correlation between pulmonary function and sleep quality in children with asthma, as well as significantly different sleep patterns in stable asthmatics compared with healthy controls. van der Kamp et al [3] revealed a significantly earlier wake-up time in uncontrolled asthmatics compared with controlled asthmatics [3]. In the study by Reynolds et al [122], children with more asthma-related comorbidities (such as allergic rhinitis, overweight, and sleep-disordered breathing) were at greater risk for shorter sleep duration.

#### Audiovisual Measures Domain

Twenty-seven studies were found, and all used sound recordings, focusing on the presence, quantity, or characterization of wheeze or cough (13 studies focused on wheeze, 8 on cough, and 5 on both), mainly in primary school children (20/27, 74%). None of the retrieved studies used video recordings. There were several ways that cough or wheeze were recorded at home, such as microphones placed in the room [22], close to the mouth [125-129], or at the chest or trachea [128-130]. Yu et al [131] used a soft stethoscope that reduced ambient sounds, and Satat et al [132] used an array of stethoscopes to allow localization of respiratory sounds. Electromyography measurements in addition to audio recordings of cough can be helpful to reduce misinterpretation of audio signals [22,133,134]. From 2015 onwards, smartphones were increasingly used as a recording device for respiratory sounds [135].

Already in 1985, Archer et al [136] reported that cough recordings were a feasible way of objectifying nocturnal cough symptoms at home, and that objective measurements did not correlate with diary card scores reported by parents [137]. Bentur et al [58] found a similar result regarding wheezing.

Many studies focused on the development of algorithms to accurately identify wheeze [128,129,135,138,139] or cough [118,126,140,141] by signal processing. Wheezing algorithms traditionally used short-time Fourier transformations to retrieve dominant sound components and extract identifying wheezing features [128,139]. New methods have been developed to increase its sensitivity for low-intensity wheezes [129] and to decrease the computation time for real-time application [138]. Cough algorithms often used both temporal and spectrum analysis techniques to retrieve relevant characteristics [126,140]. Machine learning approaches, such as hidden Markov models, could then be used to combine relevant characteristics in order to distinguish silence, background noise, and cough [141].

The first development and pilot studies on cough monitoring showed some significant differentiating characteristics between asthmatic and nonasthmatic cough; however, there was a considerable error rate, suggesting that multivariate analysis would be required for accurate discrimination [125-127].

The cough count was the primary parameter used for monitoring purposes. Several studies found that cough occurred more frequently in children with current wheezing compared to asymptomatic children (39% vs 19%) [137] and also in children with mild asthma compared to healthy children [133]. Rietveld et al [55] showed that explicitly during exacerbations, children with asthma coughed significantly more than children without asthma. When cough counts were monitored over time, low to moderate temporal correlations were found with conventional measures of asthma symptoms and symptom control [142].

Several studies investigated the correlation of the presence of wheezing and lung function parameters. The diagnostic sensitivity and specificity of wheezing for a reduction in PEF of >20% were 88% and 92%, respectively [56]. Patients with objective nocturnal wheezing were characterized by a low morning FEV1 (51%) and a larger diurnal variation in FEV1 [58], and the nocturnal wheeze rate corresponded well with changes in FEV1 and symptom scores [143].

#### Other Physiological Measurements Domain

Fourteen studies were found, and of these, 11 described measurements related to heart rate [3,21,24,31,34,123,144-148]. Other measurements involved respiratory rate [3,123], electromyography [137], and pulse oximetry [22]. Most studies focused on younger children (aged <12 years). Starting around 2017, some studies combined different wearable sensors [24,31,34] or integrated sensors with smart watches [21].

Several studies found a relation between physiological monitoring data and asthma control. Kazuma et al [144-146] in their studies from 1997 and 2000 showed decreased heart rate variability with seasonal variation in children with asthma compared to healthy controls. Huffaker et al [123] showed that heart rate parameters during nighttime were able to predict loss of asthma control before the subject’s perception of symptoms with high specificity and accuracy, but low sensitivity. van der Kamp et al [3] discovered a prolonged respiratory and heart rate recovery time after exercise in patients with uncontrolled asthma compared to patients with controlled asthma and healthy controls. Additionally, patients with uncontrolled asthma showed a higher nighttime respiratory rate.

##### Intervention Studies

One systematic review was found, which revealed no evidence to support or refute the added value of pulse oximeters to self-monitor oxygen saturation levels as part of home monitoring using a personalized asthma action plan, since the systematic search yielded zero randomized controlled trial studies [149].

#### Questionnaires Domain

Twenty-four studies were found, and they focused on both primary school children and adolescents. Digital questionnaires could be divided into asthma symptom questionnaires, such as the Childhood Asthma Control Test (ACT) or Asthma Control Questionnaire (ACQ), and ecological momentary assessment, which is the collection of data from individuals in their own environment close to the occurrence to capture momentary experiences, such as thoughts, behaviors, or symptoms, when they occur. This can provide context to gathered data and prevent recall bias [31,150-152].

When comparing electronic and paper questionnaires, equal symptom scores and missing answers [153,154] and good internal consistency [155] were reported. Vargas et al [156] described higher concordance of a nurse interview and an electronic questionnaire compared to a paper questionnaire. Patients and their parents preferred electronic questionnaires over paper questionnaires [155], and electronic questionnaires tended to be classified as feasible [155-157]. van Vliet et al [158] stated that combining electronic symptom questionnaires and spirometry provides a more realistic view on asthma control than retrospective assessment during consultations in the hospital; however, adherence to electronic questionnaires and spirometry monitoring was low [158].

Nkoy et al [159] adjusted the ACT so that it could be used as a weekly monitoring tool and included a color-coded visual representation of symptom severity, which was shown to be reliable, valid, and responsive to change over time. Digital questionnaires were also used to validate other home-monitoring devices, such as FeNO [50,53], air quality [38,44], and cough sensors [142].

##### Intervention Studies

Eleven intervention studies using digital questionnaires were found, and of these, 8 used the ACT [14,15,96,160-164], 1 used the ACQ [165], and 2 used a different type of symptom score questionnaire [98,166]. Questionnaires were used within the interventions to keep track of asthma symptoms [96,98,160,161] or to alert health care professionals (HCPs) when symptoms increased [15,163,166]. Questionnaires were sometimes combined with other asthma control parameters (eg, lung function [98,167]) or a digital action plan [96,167].

Rikkers-Mutsaert et al [167] found a significant increase in asthma control and QoL after 3 months of “internet-based self-management” monitoring using an algorithm based on the ACQ to adjust asthma treatment when compared to usual care. However, this effect was no longer seen after 12 months [167]. Additionally, van den Wijngaart et al [15,162] showed a larger increase in the ACT score and more symptom-free days after receiving care through the “Virtual Asthma Clinic” that uses ACT monitoring compared to usual care, despite a decrease in login frequency during the 16 months of use. Asthma monitoring by use of questionnaires was cost-effective in the studies by van den Wijngaart et al; however, in the study by Beerthuizen et al [161], cost-effectiveness was not significantly proven.

#### Medication Monitoring Domain

Our search identified 39 development/validation studies on medication monitoring, focusing on both younger children (25 studies) and adolescents (27 studies; 18 studies focused on children of both age groups). The adherence rate of maintenance medication is an important and often monitored parameter [28,168,169], and other measurements at home include the assessment of inhaler technique and the timing, frequency, and location of rescue medication use. HCPs reported that the most significant benefit of medication monitoring is the ability to obtain real-time intervisit data and set alert thresholds based on the frequency of rescue inhaler use or the proportion of rescue versus controller inhaler use [170]. Others pointed out that it can be helpful to determine the context in which inhaler medication was used to assess the reasons for nonadherence [150,171,172].

Several ways of monitoring adherence were described, including electronic dose counters [3,33,42,169,173-185], electronic self-report of adherence [118,186], ecological momentary assessment [150,151,172], a chatbot that informs about medication adherence [187], and monitoring of inhaler adherence or technique through video or daily phone diary [49,151]. Electronic dose counters usually have the ability to register the amount of puffs used and sometimes to remind when the canister is empty [188] or when a dose is missed [189].

Electronic dose counters have shown satisfactory feasibility [189] and reliability [174,180] to remotely monitor real-time medication use, with 98% of returned monitors fully functioning and only 3.5% data loss [174]. The most common failure was actuation underrecording, indicating the importance of quality control [180]. Reviews concluded that electronic monitors are accurate but more costly than other methods of medication monitoring [168,190,191]. Chen et al [173,176] therefore described the development of low-cost electronic dose counters. Limited studies investigated inhaler technique monitoring. Nichols et al [151] concluded that monitoring of inhaler technique by video capture was feasible, and Nikander et al [184] showed a stable inhaler technique over time, even with declining adherence.

Several studies compared the accuracy of electronic adherence monitoring and self-report [178,185,191,192]. Pearce et al [191] and Bender et al [185] reported that self-report is insufficient to provide a stand-alone measure of adherence, as adherence is generally overreported in interviews, and others concluded that electronic dose counters are more accurate than adherence self-report [192] and adequately reliable when validated to canister weight [31]. Butz et al [178] furthermore concluded that electronic medication monitoring is a more precise measure of long-term medication use than self-report on diary cards, but that diary cards seem to be a valid alternative for short-term monitoring of medication use.

Increased usage of reliever medication was correlated with worse asthma control [3,172]. Walders et al [177] however stated that monitoring controller medication adherence may be more predictive of long-term morbidity than rescue medication use.

##### Intervention Studies

Sixty-nine intervention studies were found, and of these, 23% (16/69) were review or opinion articles. Boutopoulou et al [193] emphasized the remarkable heterogeneity between adherence assessment tools, although electronic metered-dose inhaler counters were the most common type of medication monitoring tool used in the intervention studies (over two-thirds [36/53, 68%] of studies). Most (38/53, 72%) medication monitoring interventions exclusively used medication monitoring as a single monitoring domain. A total of 9 studies (9/69, 13%) reported the combined use of medication monitoring with lung function monitoring [34,89,92,101,103,108,194-197]. Only Bui et al [34] reported a pilot intervention of a multimodal monitoring intervention including medication monitoring.

Katwa et al [198] described that a device to monitor adherence ideally reminds patients about taking medication. Overall, 40% (21/53) of intervention studies used medication reminders as a tool to increase adherence. Two distinct ways of reminding were (1) standard reminders [199-202] and (2) reminders based on monitored use [203-207]. Some studies used automatic reminders (text message feedback [206], audiovisual reminders in the device [204], and app-based reminders [207]), whereas other studies had nurses [205,208] or pharmacists [78,200,209] providing feedback. Moreover, 2 studies used the pharmacy refill data to remind patients that their inhaled corticosteroids prescription would soon be overdue [210,211].

Moreover, several protocol articles on eHealth and medication monitoring were found [78,196,197,209,212-215], and all mentioned that electronic medication monitoring will be used. In most of these protocols, medication adherence will be monitored and reminders will be used to optimize adherence [129,196,197,212,214,215]. Sportel et al [196] will also measure the inhalation technique with a smart inhaler device that can measure inhalation flow, duration, and orientation based on accelerometer sensors in the add-on device.

##### Usability and Feasibility

User experiences with electronic medication monitors vary when used in intervention studies. Interviews with HCPs revealed nearly unanimous agreement on the importance of electronic medication monitoring in outpatient asthma management [216]. It provides patients with an opportunity to demonstrate the responsibility they have for their condition and allows them to experience a greater sense of independence [217]. Several studies showed satisfactory feasibility of electronic monitoring [218-220]. Children and caregivers prefer devices that provide objective measures of activation, inhalation, and technique that are accurate, require little effort, and are easy to use and fit into existing routines [221,222]. Howard et al [217] reported on adolescents’ perspectives with regard to the use of automatic reminders in electronic medication monitoring and found that despite appreciating the helpfulness and overall benefit, many participants indicate that reminders could be annoying.

Feasibility concerns with electronic monitors were data transmission failure or data loss [223-225], device loss [225], and misfit of the device and canister [224]. Implementation of medication monitoring in clinical practice also revealed the challenges of the integration of sensor data with electronic health records and the adequate education of clinical staff to work with the electronic medication monitors and their generated data [216].

##### Effects on Adherence

In several eHealth intervention studies, automatic dose counters [194,202,226,227], pharmacy refill data [208,228], or digital diaries [195,199,201] were only used to assess medication adherence as an outcome measure, whereas other studies intervened based on medication monitoring data [190,203,229]. All studies that used reminders based on electronic medication monitoring showed a positive effect of reminders on inhaled corticosteroid adherence [203-206,210,211,230]. Furthermore, Kosse et al [200,231] showed that increased activity in a digital chat with an HCP correlated with improved self-reported adherence, while other mobile app functionalities like therapy education, peer chat, and symptom questionnaires did not affect adherence.

Monitoring interventions (with or without reminders) yielded positive effects; however, effect sizes were variable [190,203,229]. In the pooled analysis of Jeminiwa et al [10], the comparison of an eHealth intervention and control indicated a small but significant effect on medication adherence (standard mean difference=0.41; 95% CI 0.02-0.79). This was especially true for mobile health studies including audiovisual and text message reminders. Moreover, some specifically investigated nonadherence rates among pediatric asthma patients showed a decline in nonadherence rates using electronic medication monitoring with feedback [229].

Data on the sustained effects of improvement in adherence following medication monitoring are limited. Behrooz et al [230] showed a decrease in electronically measured adherence over a 12-week period while the intervention continued, whereas Spaulding et al [232] showed a sustained effect of electronic monitoring and feedback on medication adherence in their small pilot study up until 30 days after they stopped feedback (5 patients).

##### Effects on Asthma Outcomes

Increased asthma control, and reduced night-time wheezing, emergency department visits, and oral corticosteroid use were reported with sensor-based medication monitoring [233], whereas others showed no significant improvement in asthma outcomes [210,234]. A systematic review by Adejumo et al [235] showed meaningful sustained improvement in asthma-related outcomes in 20% of the included studies.

##### Monitoring Reliever Use and Inhalation Technique

While most studies focused on monitoring controller inhalation therapy, 3 intervention studies focused on monitoring rescue inhalation therapy [236-238]. These studies showed that monitoring SABA use and providing feedback led to a reduction in SABA use and an increase in symptom-free days [236-238]. Moreover, Barrett et al [236] used electronic monitoring of SABA use in combination with air quality data to identify geographical “hot spots” triggering asthma, and the data were used for policy recommendations regarding improvement of air quality.

Although the inhalation technique is an important aspect to achieve good effects of inhalation therapy, only 9 studies reported on monitoring of the inhalation technique at home by use of eHealth [92,239,240]. In these articles, there were 2 distinct ways of monitoring the inhalation technique: remote observation of therapy [92,239,240] and electronic measurement of aspects of the inhalation technique [89,196,232]. It was demonstrated that audiovisual support regarding the inhalation technique is feasible [229], can help to improve effective medication use by providing feedback on the inhalation technique [92,239,240], and can lead to improved ACT scores [229]. Bynum et al [240] showed that education and feedback for the metered-dose inhaler technique by video consultation were more effective than written instructions.

#### Digital Environment Domain

Our search identified 83 development and validation studies. Digital interventions used different strategies to allow remote monitoring, such as diaries and communication tools. Education, digital action plans, and automatic reminders were other components of digital environments to support asthma monitoring and treatment.

Several development studies emphasized the importance of patient-centered design approaches to meet the needs and priorities of users [241-247]. Jácome et al [248] reported that two-thirds of asthma patients expressed interest in using an app to manage their asthma, which was similar to the finding in patients with other health conditions. Interviews with patients about user preferences revealed which features of hypothetical apps were deemed important, such as reminders [230,247,249,250], tracking [230,247,249,250], social interaction [247,250,251], educational content [230,246,247,252], emergency support [247,250], and expert access [247,250]. Furthermore, the preference of customization of app features according to preferences and schedules was mentioned in several studies [247,249,253-257]. Moreover, younger children and adolescents showed different needs and user patterns regarding asthma apps [162].

The facilitators for using digital environments for pediatric asthma management were enthusiastic initiators, tailoring of care to individual patients, and long-term profit and efficiency [164]. The main barriers for the use of apps were technical problems or loss of data due to software updates [224], loss of devices [224], concerns about privacy [258], integration with the electronic medical record [164], increased clinical workload [164], and lack of financial reimbursement for services outside the routine of HCPs [258]. Meischke et al [259] showed that demographics and computer-related variables were not related to the engagement of interactive web programs.

Digital environments are increasingly including options to acquire and share monitoring data from previously mentioned monitoring domains, including medication monitoring [34,151,189,224,231,236,256], lung function [34,151,189,224,231,236,256], air pollution [23,24,28,31,33-35,124,236,260,261], and sleep or activity [28,33,34,118]. Digital environments enable easy interpretation using simple visualization of monitoring data [21,24]. Real-time analytics through clinical decision algorithms could eventually provide medical recommendations based on actual monitoring data [24].

##### Monitoring Components

A digital diary was often included to monitor asthma features, such as a report of personal asthma symptoms, medication use, or activity [28,33,118,124,153,186,187,199,200,249, 252,262-266]. Furthermore, several studies used predefined methods of logging, such as recurrent online questionnaires [35,54,124,249,259,267-271] or ecological momentary assessment [28,31,151,183]. Simple diary visualization by using icons in a digital calendar can be comprehended by primary school children and is therefore a usable tool to report data [264]. Equivalent [153] or better [265] test-retest reliability was reported for eDiaries compared to paper-and-pencil diaries, with a higher compliance [265].

Over 25% (21/83) of studies on digital interventions described the use of some form of online communication with peers, physicians, nurses, pharmacists, or other HCPs within the intervention. The different forms of communication included chatting [187,264,272], text messaging [224,257,258,267,273,274], video consulting [275], and calling [208,276]. Several studies reported the use of automated chatbots, which revealed good usability and the ability to elicit daily responses [187,264,272]. Yoo et al [250] described that most online communication tools were task focused instead of socioemotional. Roberts et al [277] showed that the majority of adolescents believed that apps could enhance communication with their medical provider and give them more control in the patient-provider relationship. This was also recognized in a pilot study by Haze et al [267], where teenagers and HCPs perceived improved access and quick response times when using a telephone app to communicate.

##### Supportive Components

Many studies included some form of educational content [187,200,219,231,242,243,249,252,262,263,267,276,278-281]. Others included education by peer support as sharing experiences can help to learn practical skills such as managing asthma, seeking support, or self-advocacy [251,263]. Schneider et al [243] demonstrated that patients preferred education through concise text or short videos (<10 min). Moreover, over the past years, asthma action plans have been integrated into digital asthma care technology [28,33,267,268,271,278,282]. Odom et al [268] showed the positive feasibility of digital asthma action plans.

Gamification was sometimes used with the aim to improve asthma knowledge and management [249,261,278,283], or more specifically improve treatment adherence [199,203] or spirometric adherence and quality [75,76]. An example of a visual incentive to improve home spirometry is real-time feedback through a dragon that spits fire based on spirometric performance [75,76]. Another game element that was often included to achieve and maintain compliance was a reward system [199].

Furthermore, automatic reminders were integrated into many digital environments, for example, to send sensor data or take maintenance medication [35,67,118,187,199,200,203,231,249, 263,276,282,284].

##### Intervention Studies

Seventy-six intervention studies were found within the digital intervention domain. Digital interventions for optimal and personalized asthma management included a range of digital tools for self-monitoring of symptoms or disease control, and addressed self-management action plans and patient educational materials [285]. The interventions were mostly performed by a nurse or were app based, and sometimes a medical specialist, pharmacist, or other HCP performed the intervention. Overall, 74% (56/76) of these interventions were mobile based, especially in the last few years. Future studies will focus on a variety of digital interventions, often combining different monitoring tools, such as a diary, lung function measurements, medication adherence, and ACT scores [117,152,197,209,213,285,286], with a trend toward more automatization in remote monitoring [117,209,213].

Several reviews showed that digital interventions can be beneficial for adherence, asthma self-management, and asthma control [7,8,287-289]. The effects of digital interventions were categorized into the following outcome measures: symptoms, QoL, lung function, adherence, self-management, health care use, and school absence.

##### Symptoms

Twenty-two studies showed significant asthma-related symptom reduction [8,14,98,290], whereas 9 studies showed no effect of the intervention on asthma symptoms [52,194,202,291-296]. Larger effects on asthma symptoms were reported when children had uncontrolled asthma at baseline [189,211,297].

Asthma questionnaires, such as the ACT [14,65,93,94,96,98,163,195,202,237,290,291,298-300] and ACQ [208,301] were often used to report asthma symptoms. Other measures used to specify asthma symptoms were SABA use [236-238] and symptom-free days [236,237,294,302] or alternative quantitative or qualitative methods to assess asthma symptoms. Overall, 87% (20/23) of the studies that showed significant improvements in asthma symptoms used a quantitative measure (eg, ACT, ACQ, symptom-free days, or SABA use).

Although some studies within the development and validation phase focused on symptom perception [118,245], no intervention studies reported on symptom perception as an outcome measure.

##### QoL

Of the 19 studies that reported on the effect regarding QoL, 10 reported a positive effect [8,98,290,303] and the other 9 reported no difference [14,293,304,305] regarding the studied intervention. There was no clear difference in the types of interventions that had significant or no significant effect on QoL.

##### Lung Function

A limited number of studies used lung function as an outcome measure. Five studies showed an improvement in lung function [8,92,95,98,289], whereas 4 studies showed equal lung function after the intervention [52,94,195,302]. There was no clear difference in the types of interventions that had significant or no significant effect on lung function outcomes.

##### Adherence

Most studies (19/23, 83%) showed a positive effect of their digital intervention on controller medication adherence [96,98,304,305], whereas few studies showed no effect [208,297]. This corresponds with the review results of Ramsey et al [8], who showed that 87% of digital interventions improved adherence. Furthermore, Fiks et al [228] reported an improvement only in children with uncontrolled asthma at baseline, and Wiecha et al [194] only reported improvement when baseline adherence was poor. Factors that had a positive impact on adherence were related to active involvement with the intervention, such as regular use of an app [96] and the use of the provided chat function [231].

##### Self-management

Different measures were used to define self-management, such as asthma knowledge score, self-confidence, support-seeking, coping, and self-efficacy score. Five studies showed an improvement in self-management [98,103,290,300,306], whereas 2 studies showed no effect [93,208]. The review by Tan et al [307] scored different asthma apps on a list of 6 self-management principles and reported that many apps fell short on these self-management principles. The 2 main components of digital interventions that reported on the influence on asthma self-management were (1) online education [98,103,290,300,306] and (2) the use of a digital asthma action plan [93,300].

##### Health Care Use and Costs

Within the studies that reported on health care use and costs, 2 types of interventions could be identified. The first type of intervention tried to replace current outpatient visits with digital alternatives, such as teleconsultation [291,302,308] or digital symptom monitoring [14,15]. These studies all reported reductions in outpatient care [14,291,302,308]. The Cochrane review by Kew et al [291] concluded that current randomized evidence does not demonstrate any important differences between face-to-face and remote asthma check-ups in terms of exacerbations, asthma control, or QoL. The second type of digital intervention aimed to reduce total or urgent health care use by improving asthma management and asthma control. Within this category, 6 studies showed reduced health care use [163,289,298,305,309,310], while 4 studies showed no significant change in health care use [14,87,293,297,311]. It is remarkable that although many studies adopted urgent care use as an outcome measure (emergency visits and hospital admissions), few studies reported on health care costs or cost effectiveness [298,311].

##### School Absence

Four studies showed a positive effect of the intervention on school absence [8,163,290,298], whereas 8 studies showed no significant effect [14,194,289,294,296,297,305]. A common factor in successful interventions was active involvement or interaction with HCPs after the digital intervention in terms of personalized feedback [290], nurse-led management [298], or proactive contact when issues were identified [163].

## Discussion

### Reflection on Review Results

In this scoping review, we identified and analyzed the relevant monitoring domains of the large spectrum of eHealth technologies for the monitoring and treatment of pediatric asthma. We identified the noninferiority and potential superiority of eHealth monitoring, although there was a large heterogeneity in study designs and outcome measures. This review furthermore revealed limited literature on multiparameter monitoring strategies.

The application of eHealth technologies has shown an accelerated increase in the last couple of years. The outcomes of the review showed the potency for efficacy of most monitoring domains, with the requirement of further research to eventually achieve benefits in the treatment of pediatric asthma. Medication monitoring appeared to be an important domain, with an overall positive effect on adherence as well as asthma outcomes. However, the potential bias of study participation on increasing medication adherence should be taken into account. The digital environment domain showed that mobile-based apps especially promoted easy use of eHealth tools and provided support in monitoring and treatment through various functionalities (such as diaries, digital action plans, and education). Studies in the review furthermore indicated that acceptable and reproducible lung function monitoring could be performed at home, which may allow the objective follow-up of airway obstruction over time. Digital questionnaires seemed useful to monitor asthma symptoms in an easy and low-cost way. The remaining domains (sleep, audiovisual, and other physiological measurements) showed potential to objectively map manifestations of asthma, but there was a lack of intervention studies to determine their added value for the monitoring or treatment of pediatric asthma. Monitoring air quality and physical activity can provide context to asthma control monitoring and potentially fit within a multiparameter monitoring strategy. Contrary to other domains, airway inflammation markers (ie, FeNO) seemed to show no additional benefit for home monitoring of asthma and may have better effectiveness as targeted diagnostics in specific patients in a hospital setting [379,380]. These eHealth developments match the main focus of asthma monitoring in the GINA asthma guideline, indicating that asthma monitoring should include assessment of asthma control parameters, treatment issues, and other factors that contribute to symptom burden and poor QoL.

The development of eHealth technologies has become more user-centered and focused on digital connectivity and integration into existing health systems, as well as feasibility and usability. Validation studies focused on the validity of home-based measurements compared to hospital-based measurements and the correlation of home-measured data with asthma outcomes. Increasing numbers of intervention studies were seen from 2014, and they focused on the application and implementation of eHealth technologies, with various pragmatic study designs.

Although the heterogeneity of eHealth study designs and outcome measures complicates comparability, this review showed that eHealth technologies may benefit health outcomes and may at least show no adverse effects on asthma-related health outcomes or asthma management. Scoping reviews for eHealth in other fields have confirmed the heterogeneity in study outcomes [16,17] and have found sparse evidence on negative outcomes or adverse effects [381,382]. This may indicate either the overall noninferiority of eHealth efficacy or the underreporting of adverse outcomes [383].

### Future Perspectives

The domain-specific discussions of results, research gaps, and future research opportunities are presented in the domain-specific sections in Multimedia Appendix 3 [2,3,5,8,10,13,22-24,28,31,33,34,55,58,59,64,66-70,73,75,76,78,83-85, 87-90,92,96,102,113,118,120,121,123,125-132,144-146,155, 156,168,169,176,179,181,189,196,211, 212,228,229,231,239,240,248,297,327,333,343,353,374,375,384-411], and the future research opportunities are summarized in Table 2.

Overarching future directions of studies in the field of eHealth technologies for pediatric asthma care are discussed below, and they involve: (1) modifying potentially relevant hospital-based diagnostics for the home setting, and (2) developing multiparameter monitoring strategies (combining parameters from different monitoring domains), with the aim to adequately assess the factors influencing asthma control and provide personalized asthma treatment.

This review focused on eHealth technologies that were applied in the home setting. Hospital-based monitoring techniques and parameters might also potentially benefit home monitoring. Some hospital-based studies may use similar monitoring techniques and parameters as found in the included home-based studies (such as oxygen saturation, acoustic parameters, and lung function indices). Future research on these studies can enhance the understanding of these parameters, the monitoring domains, and the relations to clinical asthma outcomes. On the other hand, some hospital-based studies may provide new diagnostic opportunities such as bioimpedance [412], airway resistance during tidal breathing [413], forced oscillation technique [414], exhaled breath profiles and temperatures [415], diaphragm electromyography [416], and plethysmography variability [417-419]. Future research is needed to specifically review which hospital-based monitoring technologies may be beneficial for the home assessment of pediatric asthma. This may bring additional clinically relevant diagnostic technologies to the home environment, allowing assessment of real-life symptomatic periods, and may enable temporal monitoring strategies following the fluctuating asthma course and possibly provide opportunities to design cost-effective eHealth monitoring strategies.

This scoping review revealed several common overlapping monitoring domains, such as questionnaires and digital environment, lung function measurements and air quality, and medication monitoring and digital environment. However, limited literature on multiparameter monitoring using three or more domains was found. Childhood asthma however is a heterogenous and dynamic disease, which encompasses different phenotypes and variable clinical manifestations depending on, for example, the disease course, asthma management, and environmental influences [420,421]. In daily practice, the pediatrician combines and weighs the dynamics of different medical domains, such as anamnesis, physical examination, diagnostic tests, and previous experiences, for clinical decision-making, in order to optimize the assessment of asthma control. Most home diagnostics only encompass information of a specific asthma domain, lacking sensitivity to assess the control of asthma. Combining different home-monitoring domains and allowing multiparameter monitoring could promote the identification of personal cues of disruption of asthma control. This may facilitate the development of effective and personalized health care strategies and decision-making through monitoring of personal disrupting cues in order to allow timely short-term detection of asthma control deterioration [422]. The lack of multiparameter eHealth intervention studies emphasizes the need to further investigate multidomain monitoring. Furthermore, advanced analyses of multiparameter-generated data, such as machine learning and artificial intelligence data, could reveal new knowledge regarding asthma classification, monitoring, and treatment from different domains of home-monitoring data [423].

**Table 2:** Summary of domain-specific research opportunities.

Domain	Future research opportunities
Air quality	Investigate the added value to other asthma monitoring tools and the application or air quality monitoring in multiparameter asthma interventions
Airway inflammation markers	Not applicable
Lung function	Investigate the effects on asthma outcomes of home-monitoring interventions where patients perform additional lung function measurements when they experience symptoms or measure reversibility
Physical activity	Perform a systematic evaluation of the relation between physical activity and asthma control Investigate how physical activity monitoring can be applied to benefit asthma outcomes
Sleep	Investigate what sleep parameters are most strongly related to asthma control and asthma manifestations Investigate what factors influence the relation between sleep parameters and asthma control in children with asthma, such as age, sleep behavior, and chronic rhinitis
Audiovisual	Investigate if there are specific patient characteristics (eg, age, sex, and asthma severity) to identify children who benefit most from audiovisual monitoring Investigate the added value of visual recordings compared to audio recordings alone
Other physiological measurements	Investigate the added value of other physiological parameters (such as heart rate, respiratory rate, pulse oximetry, and electromyography) in asthma monitoring Investigate the added value of other physiological measurements in a multiparameter asthma monitoring strategy at home.
Questionnaires	Investigate if there are specific patient characteristics (eg, age, sex, and asthma severity) that can identify children with a good symptom perception who can be adequately monitored by questionnaires and children with a poor symptom perception who require additional monitoring Investigate the added value of questionnaire monitoring in multiparameter asthma monitoring
Medication monitoring	Investigate the long-term effects of medication monitoring interventions on adherence and asthma outcomes Investigate the best personalized strategies to provide feedback based on medication monitoring data to optimize adherence
Digital environment	Investigate which components of digital environments are most effective Investigate how digital environments for pediatric asthma can be optimized to maximize effectiveness for health outcomes

### Strengths and Limitations

This study provides a unique broad overview of all pediatric asthma eHealth home-monitoring literature as it reviewed the available evidence of monitoring domains and identified future directions in all monitoring domains. A disadvantage of such a broad scoping search was the large number of studies in both the title/abstract screening and the full-text review, which led to the limitation of no second search. For systematic reviews, a second search close to publication is preferred to ensure that the review is up-to-date [424]. However, in this case, with the expanding field of eHealth and the use of a broad scoping review question, there would have been a delay in providing information, and timely publication of scoping information may particularly be of great relevance in the current rapidly advancing field, allowing the identification of research gaps and future research opportunities [18,425].

### Conclusion

This systematic scoping review provides a unique overview of eHealth pediatric asthma monitoring studies, and it revealed that eHealth research takes place throughout different monitoring domains using different approaches. Moreover, it seemed that intervention outcomes of eHealth pediatric asthma monitoring are noninferior and show potential superiority. Future studies could focus on combining home-monitoring domains to facilitate multiparameter decision-making and personalized clinical decision support.

## Appendix

Multimedia Appendix 1 PubMed search strategy.

Multimedia Appendix 2 Included studies.

Multimedia Appendix 3 Domain-specific discussion and future perspectives.

## Abbreviations

ACQ: Asthma Control Questionnaire
ACT: Asthma Control Test
FeNO: fractional exhaled nitric oxide
FEV1: forced expiratory volume in 1 second
GINA: Global Initiative for Asthma
HCP: health care professional
PEF: peak expiratory flow
QoL: quality of life
SABA: short-acting beta-agonist

## Data Availability

The data sets generated during or analyzed during this study are available from the corresponding author on reasonable request.

## References

[1] Asher MI, Montefort S, Björkstén B, Lai CK, Strachan DP, Weiland SK, Williams H (2006). Worldwide time trends in the prevalence of symptoms of asthma, allergic rhinoconjunctivitis, and eczema in childhood: ISAAC Phases One and Three repeat multicountry cross-sectional surveys. The Lancet.

[2] van der Kamp M, Reimering Hartgerink P, Driessen J, Thio B, Hermens H, Tabak M (2021). Feasibility, Efficacy, and Efficiency of eHealth-Supported Pediatric Asthma Care: Six-Month Quasi-Experimental Single-Arm Pretest-Posttest Study. JMIR Form Res.

[3] van der Kamp MR, Klaver EC, Thio BJ, Driessen JMM, de Jongh FHC, Tabak M, van der Palen J, Hermens HJ (2020). WEARCON: wearable home monitoring in children with asthma reveals a strong association with hospital based assessment of asthma control. BMC Med Inform Decis Mak.

[4] Pijnenburg M, Baraldi E, Brand P, Carlsen KA, Eber E, Frischer T, Hedlin G, Kulkarni N, Lex C, Mäkelä M, Mantzouranis E, Moeller A, Pavord I, Piacentini G, Price D, Rottier B, Saglani S, Sly P, Szefler S, Tonia T, Turner S, Wooler E, Lødrup Carlsen K (2015). Monitoring asthma in children. Eur Respir J.

[5] Global Initiative for Asthma – GINA.

[6] Wu YP, Steele RG, Connelly MA, Palermo TM, Ritterband LM (2014). Commentary: pediatric eHealth interventions: common challenges during development, implementation, and dissemination. J Pediatr Psychol.

[7] Alquran A, Lambert K, Farouque A, Holland A, Davies J, Lampugnani E, Erbas B (2018). Smartphone Applications for Encouraging Asthma Self-Management in Adolescents: A Systematic Review. Int J Environ Res Public Health.

[8] Ramsey R, Plevinsky J, Kollin S, Gibler R, Guilbert T, Hommel K (2020). Systematic Review of Digital Interventions for Pediatric Asthma Management. J Allergy Clin Immunol Pract.

[9] Betz CL, Lewinter K, Kysh L, Hudson S, Espinoza J (2019). Smart devices for the management of pediatric asthma. JBI Database of Systematic Reviews and Implementation Reports.

[10] Jeminiwa R, Hohmann L, Qian J, Garza K, Hansen R, Fox B (2019). Impact of eHealth on medication adherence among patients with asthma: A systematic review and meta-analysis. Respir Med.

[11] Morrison D, Wyke S, Agur K, Cameron EJ, Docking RI, Mackenzie AM, McConnachie A, Raghuvir V, Thomson NC, Mair FS (2014). Digital asthma self-management interventions: a systematic review. J Med Internet Res.

[12] Unni E, Gabriel S, Ariely R (2018). A review of the use and effectiveness of digital health technologies in patients with asthma. Ann Allergy Asthma Immunol.

[13] Ferrante G, Licari A, Marseglia G, La Grutta S (2021). Digital health interventions in children with asthma. Clin Exp Allergy.

[14] van den Wijngaart LS, Kievit W, Roukema J, Boehmer AL, Brouwer ML, Hugen CA, Niers LE, Sprij AJ, Rikkers-Mutsaerts ER, Rottier BL, Verhaak CM, Pijnenburg MW, Merkus PJ (2017). Online asthma management for children is cost-effective. Eur Respir J.

[15] van den Wijngaart LS, Roukema J, Boehmer AL, Brouwer ML, Hugen CA, Niers LE, Sprij AJ, Rikkers-Mutsaerts ER, Rottier BL, Donders ART, Verhaak CM, Pijnenburg MW, Merkus PJ (2017). A virtual asthma clinic for children: fewer routine outpatient visits, same asthma control. Eur Respir J.

[16] Tully L, Burls A, Sorensen J, El-Moslemany R, O'Malley G (2020). Mobile Health for Pediatric Weight Management: Systematic Scoping Review. JMIR Mhealth Uhealth.

[17] Shaffer KM, Tigershtrom A, Badr H, Benvengo S, Hernandez M, Ritterband LM (2020). Dyadic Psychosocial eHealth Interventions: Systematic Scoping Review. J Med Internet Res.

[18] Lewinter KE, Hudson SM, Kysh L, Lara M, Betz CL, Espinoza J (2020). Reconsidering reviews: the role of scoping reviews in digital medicine and pediatrics. NPJ Digit Med.

[19] Munn Z, Peters M, Stern C, Tufanaru C, McArthur A, Aromataris E (2018). Systematic review or scoping review? Guidance for authors when choosing between a systematic or scoping review approach. BMC Med Res Methodol.

[20] Tricco A, Lillie E, Zarin W, O'Brien K, Colquhoun H, Levac D, Moher D, Peters M, Horsley T, Weeks L, Hempel S, Akl E, Chang C, McGowan J, Stewart L, Hartling L, Aldcroft A, Wilson M, Garritty C, Lewin S, Godfrey C, Macdonald M, Langlois E, Soares-Weiser K, Moriarty J, Clifford T, Tunçalp Ö, Straus S (2018). PRISMA Extension for Scoping Reviews (PRISMA-ScR): Checklist and Explanation. Ann Intern Med.

[21] Hosseini A, Buonocore C, Hashemzadeh S, Hojaiji H, Kalantarian H, Sideris C, Bui A, King C, Sarrafzadeh M (2016). HIPAA Compliant Wireless Sensing Smartwatch Application for the Self-Management of Pediatric Asthma. Int Conf Wearable Implant Body Sens Netw.

[22] Brooke A, Lambert P, Burton P, Clarke C, Luyt D, Simpson H (1996). Night cough in a population-based sample of children: characteristics, relation to symptoms and associations with measures of asthma severity. Eur Respir J.

[23] Fletcher R, Oreskovic N, Robinson A (2014). Design and clinical feasibility of personal wearable monitor for measurement of activity and environmental exposure. Annu Int Conf IEEE Eng Med Biol Soc.

[24] Hosseini A, Buonocore C, Hashemzadeh S, Hojaiji H, Kalantarian H, Sideris C, Bui A, King C, Sarrafzadeh M (2017). Feasibility of a Secure Wireless Sensing Smartwatch Application for the Self-Management of Pediatric Asthma. Sensors (Basel).

[25] Lin EZ, Esenther S, Mascelloni M, Irfan F, Godri Pollitt KJ (2020). The Fresh Air Wristband: A Wearable Air Pollutant Sampler. Environ. Sci. Technol. Lett.

[26] Ryan PH, Son SY, Wolfe C, Lockey J, Brokamp C, LeMasters G (2015). A field application of a personal sensor for ultrafine particle exposure in children. Sci Total Environ.

[27] Li B, Dong Q, Downen RS, Tran N, Jackson JH, Pillai D, Zaghloul M, Li Z (2019). A Wearable IoT Aldehyde Sensor for Pediatric Asthma Research and Management. Sens Actuators B Chem.

[28] Jaimini U, Thirunarayan K, Kalra M, Venkataraman R, Kadariya D, Sheth A (2018). "How Is My Child's Asthma?" Digital Phenotype and Actionable Insights for Pediatric Asthma. JMIR Pediatr Parent.

[29] Dong Q, Li B, Downen R, Tran N, Chorvinsky E, Pillai D, Zaghloul M, Li Z (2019). Wearable and Stationary Point-of-Care IoT Air Pollution Sensors for Pediatric Asthma Research and Management. 2019 IEEE Healthcare Innovations and Point of Care Technologies, (HI-POCT).

[30] Shalat SL, Stambler AA, Wang Z, Mainelis G, Emoekpere OH, Hernandez M, Lioy PJ, Black K (2011). Development and in-home testing of the Pretoddler Inhalable Particulate Environmental Robotic (PIPER Mk IV) sampler. Environ Sci Technol.

[31] Buonocore C, Rocchio R, Roman A, King C, Sarrafzadeh M (2017). Wireless Sensor-Dependent Ecological Momentary Assessment for Pediatric Asthma mHealth Applications. IEEE Int Conf Connect Health Appl Syst Eng Technol.

[32] Seto E, Giani A, Shia V, Wang C, Yan P, Yang A, Jerrett M, Bajcsy R (2009). A wireless body sensor network for the prevention and management of asthma.

[33] Venkataramanan R, Thirunarayan K, Jaimini U, Kadariya D, Yip HY, Kalra M, Sheth A (2019). Determination of Personalized Asthma Triggers From Multimodal Sensing and a Mobile App: Observational Study. JMIR Pediatr Parent.

[34] Bui A, Hosseini A, Rocchio R, Jacobs N, Ross M, Okelo S, Lurmann F, Eckel S, Dzubur E, Dunton G, Gilliland F, Sarrafzadeh M, Habre R (2020). Biomedical REAl-Time Health Evaluation (BREATHE): toward an mHealth informatics platform. JAMIA Open.

[35] Anantharam P, Banerjee T, Sheth A, Thirunarayan K, Marupudi S, Sridharan V, Forbis S (2015). Knowledge-Driven Personalized Contextual mHealth Service for Asthma Management in Children.

[36] Li S, Williams G, Jalaludin B, Baker P (2012). Panel studies of air pollution on children's lung function and respiratory symptoms: a literature review. J Asthma.

[37] Hansel NN, Breysse PN, McCormack MC, Matsui EC, Curtin-Brosnan J, Williams DL, Moore JL, Cuhran JL, Diette GB (2008). A longitudinal study of indoor nitrogen dioxide levels and respiratory symptoms in inner-city children with asthma. Environ Health Perspect.

[38] Velická H, Puklová V, Keder J, Brabec M, Malý M, Bobák M, Kotlík B, Jiřík V, Janout V, Kazmarová H (2015). Asthma Exacerbations and Symptom Variability in Children Due to Short-term Ambient Air Pollution Changes in Ostrava, Czech Republic. Cent Eur J Public Health.

[39] McCormack MC, Breysse PN, Matsui EC, Hansel NN, Williams D, Curtin-Brosnan J, Eggleston P, Diette GB, Center for Childhood Asthma in the Urban Environment (2009). In-home particle concentrations and childhood asthma morbidity. Environ Health Perspect.

[40] Lawson J, Dosman J, Rennie D, Beach J, Newman S, Senthilselvan A (2011). Relationship of endotoxin and tobacco smoke exposure to wheeze and diurnal peak expiratory flow variability in children and adolescents. Respirology.

[41] Castro HAD, Cunha MFD, Mendonça G, Junger WL, Cunha-Cruz J, Leon APD (2009). Effect of air pollution on lung function in schoolchildren in Rio de Janeiro, Brazil. Rev Saude Publica.

[42] Delfino RJ, Staimer N, Tjoa T, Gillen D, Kleinman MT, Sioutas C, Cooper D (2008). Personal and ambient air pollution exposures and lung function decrements in children with asthma. Environ Health Perspect.

[43] Spira-Cohen A (2010). The role of traffic-related air pollution in PM-health effects associations among inner city children with asthma. Dissertation Abstracts International: Section B: The Sciences and Engineering.

[44] Tang C, Chang L, Lee H, Chan C (2007). Effects of personal particulate matter on peak expiratory flow rate of asthmatic children. Sci Total Environ.

[45] Li S, Baker PJ, Jalaludin BB, Guo Y, Marks GB, Denison LS, Williams GM (2014). An Australian national panel study of diurnal temperature range and children's respiratory health. Ann Allergy Asthma Immunol.

[46] Hunt J, Byrns R, Ignarro L, Gaston B (1995). Condensed expirate nitrite as a home marker for acute asthma. Lancet.

[47] Paredi P, Loukides S, Ward S, Cramer D, Spicer M, Kharitonov SA, Barnes PJ (1998). Exhalation flow and pressure-controlled reservoir collection of exhaled nitric oxide for remote and delayed analysis. Thorax.

[48] Schiller B, Hammer J, Barben J, Trachsel D (2009). Comparability of a hand-held nitric oxide analyser with online and offline chemiluminescence-based nitric oxide measurement. Pediatr Allergy Immunol.

[49] Bodini A, Peroni D, Loiacono A, Costella S, Pigozzi R, Baraldi E, Boner AL, Piacentini GL (2007). Exhaled nitric oxide daily evaluation is effective in monitoring exposure to relevant allergens in asthmatic children. Chest.

[50] Pijnenburg M, Floor S, Hop W, De Jongste JC (2006). Daily ambulatory exhaled nitric oxide measurements in asthma. Pediatr Allergy Immunol.

[51] Vahlkvist S, Sinding M, Skamstrup K, Bisgaard H (2006). Daily home measurements of exhaled nitric oxide in asthmatic children during natural birch pollen exposure. J Allergy Clin Immunol.

[52] de Jongste JC, Carraro S, Hop WC, Baraldi E (2009). Daily Telemonitoring of Exhaled Nitric Oxide and Symptoms in the Treatment of Childhood Asthma. Am J Respir Crit Care Med.

[53] van der Valk RJP, Baraldi E, Stern G, Frey U, de Jongste JC (2012). Daily exhaled nitric oxide measurements and asthma exacerbations in children. Allergy.

[54] Ghriwati NA, Everhart R, Winter M (2020). Interactive effects of family functioning and sleep experiences on daily lung functioning in pediatric asthma: An ecological momentary assessment approach. J Asthma.

[55] Rietveld S, Rijssenbeek-Nouwens LH (1998). Diagnostics of spontaneous cough in childhood asthma: results of continuous tracheal sound recording in the homes of children. Chest.

[56] Rietveld S, Oud M, Rijssenbeek-Nouwens LH, Vaghi D, Dooijes EH (1999). Characteristics and diagnostic significance of spontaneous wheezing in children with asthma: results of continuous in vivo sound recording. J Asthma.

[57] Sadeh A, Horowitz I, Wolach-Benodis L, Wolach B (1998). Sleep and pulmonary function in children with well-controlled, stable asthma. Sleep.

[58] Bentur L, Beck R, Irving CS, Godfrey S (2004). Nocturnal Wheeze Measurement in Young Asthmatics. Pediatric Asthma, Allergy & Immunology.

[59] Bastian-Lee Y, Chavasse R, Richter H, Seddon P (2002). Assessment of a low-cost home monitoring spirometer for children. Pediatr Pulmonol.

[60] Brouwer AF, Roorda RJ, Brand PL (2007). Comparison between peak expiratory flow and FEV(1) measurements on a home spirometer and on a pneumotachograph in children with asthma. Pediatr Pulmonol.

[61] Brand P, Waalkens H, Duiverman E, van Essen-Zandvliet EE (1997). Inaccuracy of portable peak flow meters: correction is not needed. Dutch CNSLD Study Group. Acta Paediatr.

[62] Gerzon FL, Jöbsis Q, Bannier MA, Winkens B, Dompeling E (2020). Discrepancy between Lung Function Measurements at Home and in the Hospital in Children with Asthma and CF. J Clin Med.

[63] Kamps A, Roorda R, Brand P (2001). Peak flow diaries in childhood asthma are unreliable. Thorax.

[64] Brand PLP, Roorda RJ (2003). Usefulness of monitoring lung function in asthma. Arch Dis Child.

[65] Burkhart PV, Dunbar-Jacob JM, Rohay JM (2001). Accuracy of children's self-reported adherence to treatment. J Nurs Scholarsh.

[66] Greineder DK, Loane KC, Parks P (1995). Reduction in resource utilization by an asthma outreach program. Arch Pediatr Adolesc Med.

[67] Gahleitner F, Legg J, Holland E, Pearson S, Roberts G (2016). The validity and acceptability of a text-based monitoring system for pediatric asthma studies. Pediatr Pulmonol.

[68] Anees W, Huggins V, Burge P (2001). Reliability of PEF diaries. Thorax.

[69] van der Meer V, Rikkers-Mutsaerts ERVM, Sterk PJ, Thiadens HA, Assendelft WJJ, Sont JK (2006). Compliance and reliability of electronic PEF monitoring in adolescents with asthma. Thorax.

[70] Sly P, Flack F (2001). Is home monitoring of lung function worthwhile for children with asthma?. West J Med.

[71] Pelkonen AS, Nikander K, Turpeinen M (2000). Reproducibility of home spirometry in children with newly diagnosed asthma. Pediatr Pulmonol.

[72] Wensley D, Silverman M (2001). The quality of home spirometry in school children with asthma. Thorax.

[73] Mortimer KM, Fallot A, Balmes JR, Tager IB (2003). Evaluating the use of a portable spirometer in a study of pediatric asthma. Chest.

[74] Thompson R, Delfino RJ, Tjoa T, Nussbaum E, Cooper D (2006). Evaluation of daily home spirometry for school children with asthma: new insights. Pediatr Pulmonol.

[75] Pinho B, Almeida R, Jácome C, Teixeira J, Amaral R, Lopes F, Jacinto T, Guedes R, Pereira M, Gonçalves I, Fonseca J (2018). Automatic Quality Assessment of Smart Device Microphone Spirometry. Proceedings of the 8th International Joint Conference on Pervasive and Embedded Computing and Communication Systems - PECCS.

[76] van Delden R, Plass-Oude BD, de With AJV, Vogel K, Klaassen R, Zwart N, Faber J, Thio B, van der Kamp M (2020). CHI PLAY '20: Proceedings of the Annual Symposium on Computer-Human Interaction in Play.

[77] Nikkila S, Patel G, Sundaram H, Kelliher A, Sabharwal A (2012). Wind runners: designing a game to encourage medical adherence for children with asthma.

[78] De Vera MA, Sadatsafavi M, Tsao NW, Lynd LD, Lester R, Gastonguay L, Galo J, FitzGerald JM, Brasher P, Marra CA (2014). Empowering pharmacists in asthma management through interactive SMS (EmPhAsIS): study protocol for a randomized controlled trial. Trials.

[79] Uwyyed K, Springer C, Avital A, Bar-Yishay E, Godfrey S (1996). Home recording of PEF in young asthmatics: does it contribute to management?. Eur Respir J.

[80] Linna OV (1993). Twice-daily peak expiratory flow rate monitoring for the assessment of childhood asthma. Allergy Proc.

[81] Klein RB, Fritz GK, Yeung A, McQuaid EL, Mansell A (1995). Spirometric patterns in childhood asthma: peak flow compared with other indices. Pediatr Pulmonol.

[82] Cai P, Hebert M, Cowie R, Meadows L (2016). Experience with home telehealth to support disease management in teenagers with asthma. J Telemed Telecare.

[83] Foo AL, Sly PD (1991). Pulmonary function in a hospital population of asthmatic children. J Asthma.

[84] Brouwer AFJ, Roorda RJ, Brand PLP (2006). Home spirometry and asthma severity in children. Eur Respir J.

[85] Brouwer A, Brand P, Roorda R, Duiverman E (2010). Airway obstruction at time of symptoms prompting use of reliever therapy in children with asthma. Acta Paediatr.

[86] Sly PD, Cahill P, Willet K, Burton P (1994). Accuracy of mini peak flow meters in indicating changes in lung function in children with asthma. BMJ.

[87] Lloyd BW, Ali MH (1992). How useful do parents find home peak flow monitoring for children with asthma?. BMJ.

[88] Wensley D, Silverman M (2004). Peak Flow Monitoring for Guided Self-management in Childhood Asthma. Am J Respir Crit Care Med.

[89] van der Kamp MR, Tabak M, de Rooij SEJA, van Lierop PPE, Thio BJ (2020). COVID-19: Technology-Supported Remote Assessment of Pediatric Asthma at Home. Front Pediatr.

[90] Willems DCM, Joore MA, Hendriks JJE, van Duurling RAH, Wouters EFM, Severens JLY (2007). Process evaluation of a nurse-led telemonitoring programme for patients with asthma. J Telemed Telecare.

[91] Schneider T, Baum L, Amy A, Marisa C (2020). I have most of my asthma under control and I know how my asthma acts: Users' perceptions of asthma self-management mobile app tailored for adolescents. Health Informatics J.

[92] Chan D, Callahan C, Sheets S, Moreno C, Malone F (2003). An Internet-based store-and-forward video home telehealth system for improving asthma outcomes in children. Am J Health Syst Pharm.

[93] Perry TT, Marshall A, Berlinski A, Rettiganti M, Brown RH, Randle SM, Luo C, Bian J (2017). Smartphone-based vs paper-based asthma action plans for adolescents. Ann Allergy Asthma Immunol.

[94] Montalbano L, Ferrante G, Cilluffo G, Gentile M, Arrigo M, La Guardia D, Allegra M, Malizia V, Gagliardo RP, Bonini M, La Grutta S (2019). Targeting quality of life in asthmatic children: The MyTEP pilot randomized trial. Respir Med.

[95] Guendelman S, Meade K, Benson M, Chen YQ, Samuels S (2002). Improving asthma outcomes and self-management behaviors of inner-city children: a randomized trial of the Health Buddy interactive device and an asthma diary. Arch Pediatr Adolesc Med.

[96] Ljungberg H, Carleborg A, Gerber H, Öfverström C, Wolodarski J, Menshi F, Engdahl M, Eduards M, Nordlund B (2019). Clinical effect on uncontrolled asthma using a novel digital automated self-management solution: a physician-blinded randomised controlled crossover trial. Eur Respir J.

[97] Battu K, Collins-Williams C, Zaleskey C (1982). Evaluation of home-monitoring of asthmatic children with the mini-Wright peak flow meter. J Asthma.

[98] Jan R, Wang J, Huang M, Tseng S, Su H, Liu L (2007). An internet-based interactive telemonitoring system for improving childhood asthma outcomes in Taiwan. Telemed J E Health.

[99] Sly PD, Landau LI, Weymouth R (1985). Home recording of peak expiratory flow rates and perception of asthma. Am J Dis Child.

[100] Boggs PB, Hayati F, Washburne WF, Wheeler DA (1999). Using statistical process control charts for the continual improvement of asthma care. Jt Comm J Qual Improv.

[101] Murphy J (2001). Telemedicine offers new way to manage asthma. Am J Health Syst Pharm.

[102] Deschildre A, Béghin L, Salleron J, Iliescu C, Thumerelle C, Santos C, Hoorelbeke A, Scalbert M, Pouessel G, Gnansounou M, Edmé J, Matran R (2012). Home telemonitoring (forced expiratory volume in 1 s) in children with severe asthma does not reduce exacerbations. Eur Respir J.

[103] Asthma In-Home Monitoring (AIM) Trial. ClinicalTrials.gov.

[104] Ryan D, Cobern W, Wheeler J, Price D, Tarassenko L (2005). Mobile phone technology in the management of asthma. J Telemed Telecare.

[105] Savva K, Rosen E, Bolton K, Thein O, Mayet Z (2001). Effect of the whistle watch device on bronchodilator use in children with asthma. S Afr Med J.

[106] Boggs PB, Wheeler D, Washburne WF, Hayati F (1998). Peak Expiratory Flow Rate Control Chart in Asthma Care: Chart Construction and Use in Asthma Care. Annals of Allergy, Asthma & Immunology.

[107] Dinakar C, Oppenheimer J, Portnoy J, Bacharier LB, Li J, Kercsmar CM, Bernstein D, Blessing-Moore J, Khan D, Lang D, Nicklas R, Randolph C, Schuller D, Spector S, Tilles SA, Wallace D, Joint Task Force on Practice Parameters, Practice Parameter Workgroup, American Academy of Allergy‚ AsthmaImmunology, American College of Allergy‚ AsthmaImmunology (2014). Management of acute loss of asthma control in the yellow zone: a practice parameter. Ann Allergy Asthma Immunol.

[108] Myers TR (2002). Improving Patient Outcomes with Tools for Asthma Self-Monitoring. Disease Management & Health Outcomes.

[109] Janson S (1995). Value of home peak flow monitoring for asthma control. West J Med.

[110] Everhart RS, Heron KE, Leibach GG, Miadich SA (2017). Developing a Mobile Health Intervention for Low-Income, Urban Caregivers of Children with Asthma: A Pilot Study. Pediatr Allergy Immunol Pulmonol.

[111] Janssens T, Harver A (2015). Effects of Symptom Perception Interventions on Trigger Identification and Quality of Life in Children with Asthma. Pulm Med.

[112] Kamps A, Brand P (2001). Education, self-management and home peak flow monitoring in childhood asthma. Paediatr Respir Rev.

[113] Brouwer AF, Brand PL (2008). Asthma education and monitoring: what has been shown to work. Paediatr Respir Rev.

[114] Marosi A, Stiesmeyer J (2001). Improving pediatric asthma patient outcomes by incorporation of effective interventions. J Asthma.

[115] Childhood Asthma Perception Study (CAPS). ClinicalTrials.gov.

[116] DragONE Study: Acquisition and Maintenance of Paediatric Asthma Control: Usual Care vs Innovative Devices (DragONE). ClinicalTrials.gov.

[117] Telemonitoring of Lung Function by Spirometry. ClinicalTrials.gov.

[118] Rhee H, Miner S, Sterling M, Halterman JS, Fairbanks E (2014). The development of an automated device for asthma monitoring for adolescents: methodologic approach and user acceptability. JMIR Mhealth Uhealth.

[119] Jaimini U (2017). PhD Forum: Multimodal IoT and EMR Based Smart Health Application for Asthma Management in Children.

[120] Bian J, Guo Y, Xie M, Parish AE, Wardlaw I, Brown R, Modave F, Zheng D, Perry TT (2017). Exploring the Association Between Self-Reported Asthma Impact and Fitbit-Derived Sleep Quality and Physical Activity Measures in Adolescents. JMIR Mhealth Uhealth.

[121] Firrincieli V, Keller A, Ehrensberger R, Platts-Mills J, Shufflebarger C, Geldmaker B, Platts-Mills T (2005). Decreased physical activity among Head Start children with a history of wheezing: use of an accelerometer to measure activity. Pediatr Pulmonol.

[122] Reynolds K, Boergers J, Kopel S, Koinis-Mitchell D (2018). Featured Article: Multiple Comorbid Conditions, Sleep Quality and Duration, and Academic Performance in Urban Children With Asthma. J Pediatr Psychol.

[123] Huffaker MF, Carchia M, Harris BU, Kethman WC, Murphy TE, Sakarovitch CCD, Qin F, Cornfield DN (2018). Passive Nocturnal Physiologic Monitoring Enables Early Detection of Exacerbations in Children with Asthma. A Proof-of-Concept Study. Am J Respir Crit Care Med.

[124] Chan T, Hu T, Chu Y, Hwang J (2019). Assessing effects of personal behaviors and environmental exposure on asthma episodes: a diary-based approach. BMC Pulm Med.

[125] Thorpe CW, Fright WR, Toop LJ, Dawson KP (1991). A microcomputer-based interactive cough sound analysis system. Comput Methods Programs Biomed.

[126] Thorpe C, Toop L, Dawson K (1992). Towards a quantitative description of asthmatic cough sounds. Eur Respir J.

[127] Toop LJ, Thorpe CW, Fright R (1989). Cough sound analysis: a new tool for the diagnosis of asthma?. Fam Pract.

[128] Furman EG, Yakovleva EV, Malinin SV, Furman G, Sokolovsky V (2014). Computer-assisted assay of respiratory sounds of children suffering from bronchial asthma. Sovremennye Tehnologii v Medicine.

[129] Jin F, Sattar F, Goh D (2008). Automatic wheeze detection using histograms of sample entropy.

[130] Gross V, Reinke C, Dette F, Koch R, Vasilescu D, Penzel T, Koehler U (2007). Mobile nocturnal long-term monitoring of wheezing and cough. Biomedizinische Technik/Biomedical Engineering.

[131] Yu C, Tsai T, Huang S, Lin C (2013). Soft stethoscope for detecting asthma wheeze in young children. Sensors (Basel).

[132] Satat G, Ramchander K, Raskar R (2016). Identi-wheez — A device for in-home diagnosis of asthma.

[133] Li AM, Tsang TWT, Chan DFY, Lam HS, So HK, Sung RYT, Fok TF (2006). Cough frequency in children with mild asthma correlates with sputum neutrophil count. Thorax.

[134] Li AM, Lex C, Zacharasiewicz A, Wong E, Erin E, Hansel T, Wilson NM, Bush A (2003). Cough frequency in children with stable asthma: correlation with lung function, exhaled nitric oxide, and sputum eosinophil count. Thorax.

[135] Bokov P, Mahut B, Flaud P, Delclaux C (2016). Wheezing recognition algorithm using recordings of respiratory sounds at the mouth in a pediatric population. Comput Biol Med.

[136] Archer LN, Simpson H (1985). Night cough counts and diary card scores in asthma. Arch Dis Child.

[137] Munyard P, Busst C, Logan-Sinclair R, Bush A (1994). A new device for ambulatory cough recording. Pediatr Pulmonol.

[138] Yu C, Hsiao T, Tsai T, Huang S, Lin C, Vander Sloten J, Verdonck P, Nyssen M, Haueisen J (2009). Rapid wheezing detection algorithm for real-time asthma diagnosis and personal health care. 4th European Conference of the International Federation for Medical and Biological Engineering. IFMBE Proceedings, vol 22.

[139] Kang S, Karpate Y, Almulla S, Teach S, Shekhar R (2016). Automatic identification of wheezing in auscultated lung sounds.

[140] Al-Khassaweneh M, Bani Abdelrahman R (2013). A signal processing approach for the diagnosis of asthma from cough sounds. J Med Eng Technol.

[141] Sterling M, Rhee H, Bocko M (2014). Automated Cough Assessment on a Mobile Platform. J Med Eng.

[142] Rhee H, Belyea MJ, Sterling M, Bocko MF (2015). Evaluating the Validity of an Automated Device for Asthma Monitoring for Adolescents: Correlational Design. J Med Internet Res.

[143] Bentur L, Beck R, Shinawi M, Naveh T, Gavriely N (2003). Wheeze monitoring in children for assessment of nocturnal asthma and response to therapy. Eur Respir J.

[144] Kazuma N, Otsuka K, Matsuoka I, Murata M (1997). Heart rate variability during 24 hours in asthmatic children. Chronobiol Int.

[145] Kazuma N, Otsuka K, Miyakawa M, Shirase E, Matsuoka I, Murata M (2000). Seasonal variation in heart rate variability in asthmatic children. Chronobiol Int.

[146] Kazuma N, Otsuka K (2001). Seasonal variation in 1/f fluctuations of heart rate in asthmatic children. Biomed Pharmacother.

[147] Messinger AI, Deterding RR, Szefler SJ (2018). Bringing Technology to Day-to-Day Asthma Management. Am J Respir Crit Care Med.

[148] Milagro J, Gil E, Lazaro J, Seppa V, Malmberg LP, Pelkonen AS, Kotaniemi-Syrjanen A, Makela MJ, Viik J, Bailon R (2018). Nocturnal Heart Rate Variability Spectrum Characterization in Preschool Children With Asthmatic Symptoms. IEEE J. Biomed. Health Inform.

[149] Welsh E, Carr R (2015). Pulse oximeters to self monitor oxygen saturation levels as part of a personalised asthma action plan for people with asthma. Cochrane Database Syst Rev.

[150] Dzubur E, Li M, Kawabata K, Sun Y, McConnell R, Intille S, Dunton GF (2015). Design of a smartphone application to monitor stress, asthma symptoms, and asthma inhaler use. Ann Allergy Asthma Immunol.

[151] Nichols M, Miller S, Treiber F, Ruggiero K, Dawley E, Teufel Ii R (2020). Patient and Parent Perspectives on Improving Pediatric Asthma Self-Management Through a Mobile Health Intervention: Pilot Study. JMIR Form Res.

[152] Nichols M, Teufel R, Miller S, Madisetti M, Giovanni CS, Chike-Harris K, Jones L, Prentice M, Ruggiero K, Kelechi T (2020). Managing Asthma and Obesity Related Symptoms (MATADORS): An mHealth Intervention to Facilitate Symptom Self-Management among Youth. Int J Environ Res Public Health.

[153] Ireland AM, Wiklund I, Hsieh R, Dale P, O'Rourke E (2012). An electronic diary is shown to be more reliable than a paper diary: results from a randomized crossover study in patients with persistent asthma. J Asthma.

[154] Raat H, Mangunkusumo R, Mohangoo A, Juniper E, Van Der Lei J (2007). Internet and written respiratory questionnaires yield equivalent results for adolescents. Pediatr Pulmonol.

[155] Bushnell DM, Martin ML, Parasuraman B (2003). Electronic versus paper questionnaires: a further comparison in persons with asthma. J Asthma.

[156] Vargas PA, Robles E, Harris J, Radford P (2010). Using information technology to reduce asthma disparities in underserved populations: a pilot study. J Asthma.

[157] Mussaffi H, Omer R, Prais D, Mei-Zahav M, Weiss-Kasirer T, Botzer Z, Blau H (2007). Computerised paediatric asthma quality of life questionnaires in routine care. Arch Dis Child.

[158] van Vliet D, van Horck M, van de Kant K, Vaassen S, Gulikers S, Winkens B, Rosias P, Heynens J, Muris J, Essers B, Jöbsis Q, Dompeling E (2014). Electronic monitoring of symptoms and lung function to assess asthma control in children. Ann Allergy Asthma Immunol.

[159] Nkoy F, Stone B, Fassl B, Uchida D, Koopmeiners K, Halbern S, Kim E, Wilcox A, Ying J, Greene T, Mosen D, Schatz M, Maloney C (2013). Longitudinal validation of a tool for asthma self-monitoring. Pediatrics.

[160] Legorreta AP, Leung K, Berkbigler D, Evans R, Liu X (2000). Outcomes of a population-based asthma management program: quality of life, absenteeism, and utilization. Annals of Allergy, Asthma & Immunology.

[161] Beerthuizen T, Voorend-van Bergen S, van den Hout W, Vaessen-Verberne A, Brackel H, Landstra A, van den Berg N, de Jongste J, Merkus P, Pijnenburg M, Sont J (2016). Cost-effectiveness of FENO-based and web-based monitoring in paediatric asthma management: a randomised controlled trial. Thorax.

[162] van den Wijngaart L, Geldtmeijer J, Roukema J, Boehmer A, Brouwer M, Hugen C, Niers T, Sprij A, Rikkers-Mutsaerts N, Rottier B, Verhaak C, Pijnenburg M, Merkus P (2016). The virtual asthma clinic: Description and analysis of website-use. European Respiratory Journal.

[163] Nkoy F, Fassl B, Wilkins V, Johnson J, Unsicker E, Koopmeiners K, Jensen A, Frazier M, Gaddis J, Malmgren L, Williams S, Oldroyd H, Greene T, Sheng X, Uchida D, Maloney C, Stone B (2019). Ambulatory Management of Childhood Asthma Using a Novel Self-management Application. Pediatrics.

[164] van den Wijngaart LS, Geense WW, Boehmer AL, Brouwer ML, Hugen CA, van Ewijk BE, Koenen-Jacobs M, Landstra AM, Niers LE, van Onzenoort-Bokken L, Ottink MD, Rikkers-Mutsaerts ER, Groothuis I, Vaessen-Verberne AA, Roukema J, Merkus PJ (2018). Barriers and Facilitators When Implementing Web-Based Disease Monitoring and Management as a Substitution for Regular Outpatient Care in Pediatric Asthma: Qualitative Survey Study. J Med Internet Res.

[165] Garbutt JM, Banister C, Highstein G, Sterkel R, Epstein J, Bruns J, Swerczek L, Wells S, Waterman B, Strunk RC, Bloomberg GR (2010). Telephone coaching for parents of children with asthma: impact and lessons learned. Arch Pediatr Adolesc Med.

[166] Jacobson JS, Lieblein A, Fierman AH, Fishkin ER, Hutchinson VE, Rodriguez L, Serebrisky D, Chau M, Saperstein A (2009). Randomized trial of an electronic asthma monitoring system among New York City children. Am J Manag Care.

[167] Rikkers-Mutsaerts E, Winters A, Bakker M, van Stel H, van der Meer V, de Jongste J, Sont J, SMASHING Study Group (2012). Internet-based self-management compared with usual care in adolescents with asthma: a randomized controlled trial. Pediatr Pulmonol.

[168] Desai M, Oppenheimer JJ (2011). Medication adherence in the asthmatic child and adolescent. Curr Allergy Asthma Rep.

[169] Vasbinder E, Dahhan N, Wolf B, Zoer J, van den Bemt P (2010). Electronic measurement of non-compliance to inhaled corticosteroids in a multicultural population of children with asthma in Amsterdam (Compliance Objectively measured in a Multicultural Population of children Living in Amsterdam Needing inhaled Corticosteroids for Effective asthma treatment; COMPLIANCE). Pharmaceutisch Weekblad.

[170] Hollenbach J, Cushing A, Melvin E, McGowan B, Cloutier M, Manice M (2017). Understanding clinicians' attitudes toward a mobile health strategy to childhood asthma management: A qualitative study. J Asthma.

[171] Modi AC, Quittner AL (2006). Utilizing Computerized Phone Diary Procedures to Assess Health Behaviors in Family and Social Contexts. Children's Health Care.

[172] Mulvaney SA, Ho Y, Cala CM, Chen Q, Nian H, Patterson BL, Johnson KB (2013). Assessing adolescent asthma symptoms and adherence using mobile phones. J Med Internet Res.

[173] Chen CC, Liu YJ, Wen SM, Yang CC, Chue JJ, Wu CM, Huang CM (2015). Low-cost electronic dose counter for pressurized metered dose inhaler.

[174] Patel M, Pilcher J, Travers J, Perrin K, Shaw D, Black P, Weatherall M, Beasley R (2013). Use of metered-dose inhaler electronic monitoring in a real-world asthma randomized controlled trial. J Allergy Clin Immunol Pract.

[175] O'Connor SL, Bender BG, Gavin-Devitt L, Wamboldt MZ, Milgrom H, Szefler S, Rand C, Wamboldt FS (2004). Measuring adherence with the Doser CT in children with asthma. J Asthma.

[176] Chen CC, Liu YJ, Sung GN, Yang CC, Wu CM, Huang CM (2015). Smart electronic dose counter for pressurized metered dose inhaler.

[177] Walders N, Kopel SJ, Koinis-Mitchell D, McQuaid EL (2005). Patterns of quick-relief and long-term controller medication use in pediatric asthma. J Pediatr.

[178] Butz AM, Donithan M, Bollinger ME, Rand C, Thompson RE (2005). Monitoring nebulizer use in children: comparison of electronic and asthma diary data. Annals of Allergy, Asthma & Immunology.

[179] Burgess S, Sly P, Devadason S (2011). Adherence with preventive medication in childhood asthma. Pulm Med.

[180] Chan A, Stewart A, Harrison J, Black P, Mitchell E, Foster J (2017). Electronic adherence monitoring device performance and patient acceptability: a randomized control trial. Expert Rev Med Devices.

[181] Feldman JM, McQuaid EL, Klein RB, Kopel SJ, Nassau JH, Mitchell DK, Wamboldt MZ, Fritz GK (2007). Symptom perception and functional morbidity across a 1-year follow-up in pediatric asthma. Pediatr Pulmonol.

[182] Fiese BH, Wamboldt FS, Anbar RD (2005). Family asthma management routines: connections to medical adherence and quality of life. J Pediatr.

[183] Fedele DA, McConville A, Graham Thomas J, McQuaid EL, Janicke DM, Turner EM, Moon J, Abu-Hasan M (2018). Applying Interactive Mobile health to Asthma Care in Teens (AIM2ACT): Development and design of a randomized controlled trial. Contemp Clin Trials.

[184] Nikander K, Turpeinen M, Pelkonen AS, Bengtsson T, Selroos O, Haahtela T (2011). True adherence with the Turbuhaler in young children with asthma. Arch Dis Child.

[185] Bender B, Bartlett S, Rand C, Turner C, Wamboldt F, Zhang L (2007). Impact of interview mode on accuracy of child and parent report of adherence with asthma-controller medication. Pediatrics.

[186] Ferreira A, Almeida R, Jácome C, Fernandes J, Fonseca J, Vieira-Marques P (2019). How inspiring is your app? A usability take on an app for asthma medication adherence.

[187] Kadariya D, Venkataramanan R, Yip H, Kalra M, Thirunarayanan K, Sheth A (2019). kBot: Knowledge-Enabled Personalized Chatbot for Asthma Self-Management.

[188] Vui Hin T, Ramli NI (2018). Design and Development of a Non-volatile Counter for Metered Dose Inhaler (MDI). IJIE.

[189] Cushing A, Manice M, Ting A, Parides M (2016). Feasibility of a novel mHealth management system to capture and improve medication adherence among adolescents with asthma. PPA.

[190] Jentzsch N, Camargos P (2008). Methods of assessing adherence to inhaled corticosteroid therapy in children and adolescents: adherence rates and their implications for clinical practice. J Bras Pneumol.

[191] Pearce C, Fleming L (2018). Adherence to medication in children and adolescents with asthma: methods for monitoring and intervention. Expert Review of Clinical Immunology.

[192] Jentzsch NS, Camargos PAM, Colosimo EA, Bousquet J (2009). Monitoring adherence to beclomethasone in asthmatic children and adolescents through four different methods. Allergy.

[193] Boutopoulou B, Koumpagioti D, Matziou V, Priftis KN, Douros K (2018). Interventions on Adherence to Treatment in Children With Severe Asthma: A Systematic Review. Front Pediatr.

[194] Wiecha JM, Adams WG, Rybin D, Rizzodepaoli M, Keller J, Clay JM (2015). Evaluation of a web-based asthma self-management system: a randomised controlled pilot trial. BMC Pulm Med.

[195] Reece ER, Burnette AF, Lewis-Land CJ (2017). Pilot Study of Asthmawin Mobile Iphone App in the Management of Asthma. Journal of Allergy and Clinical Immunology.

[196] Sportel ET, Oude Wolcherink MJ, van der Palen J, Lenferink A, Thio BJ, Movig KLL, Brusse-Keizer MGJ (2020). Does immediate smart feedback on therapy adherence and inhalation technique improve asthma control in children with uncontrolled asthma? A study protocol of the IMAGINE I study. Trials.

[197] A mHealth Intervention to Improve Symptom Control in Children and Adolescents With Difficult-to-control Asthma. ClinicalTrials.gov.

[198] Katwa U, Rivera E (2018). Asthma Management in the Era of Smart-Medicine: Devices, Gadgets, Apps and Telemedicine. Indian J Pediatr.

[199] Farooqui N, Phillips G, Barrett C, Stukus D (2015). Acceptability of an interactive asthma management mobile health application for children and adolescents. Ann Allergy Asthma Immunol.

[200] Kosse R, Bouvy M, de Vries T, Koster E (2019). Evaluation of a mobile health intervention to support asthma self-management and adherence in the pharmacy. Int J Clin Pharm.

[201] Johnson K, Patterson B, Ho Y, Chen Q, Nian H, Davison C, Slagle J, Mulvaney S (2016). The feasibility of text reminders to improve medication adherence in adolescents with asthma. J Am Med Inform Assoc.

[202] Kenyon CC, Gruschow SM, Quarshie WO, Griffis H, Leach MC, Zorc JJ, Bryant-Stephens TC, Miller VA, Feudtner C (2019). Controller adherence following hospital discharge in high risk children: A pilot randomized trial of text message reminders. J Asthma.

[203] Mosnaim G, Li H, Martin M, Richardson D, Belice PJ, Avery E, Silberstein A, Leigh J, Kenyon R, Jones S, Bender B, Powell LH (2015). A tailored mobile health intervention to improve adherence and asthma control in minority adolescents. J Allergy Clin Immunol Pract.

[204] Chan AHY, Stewart AW, Harrison J, Camargo CA, Black PN, Mitchell EA (2015). The effect of an electronic monitoring device with audiovisual reminder function on adherence to inhaled corticosteroids and school attendance in children with asthma: a randomised controlled trial. Lancet Respir Med.

[205] Bartlett SJ, Lukk P, Butz A, Lampros-Klein F, Rand CS (2002). Enhancing medication adherence among inner-city children with asthma: results from pilot studies. J Asthma.

[206] Chen J, Xu J, Zhao L, Zhang J, Yin Y, Zhang F (2020). The effect of electronic monitoring combined with weekly feedback and reminders on adherence to inhaled corticosteroids in infants and younger children with asthma: a randomized controlled trial. Allergy Asthma Clin Immunol.

[207] Grossman B, Conner S, Mosnaim G, Albers J, Leigh J, Jones S, Kenyon R (2017). Application of Human Augmentics: A Persuasive Asthma Inhaler. J Biomed Inform.

[208] Gustafson D, Wise M, Bhattacharya A, Pulvermacher A, Shanovich K, Phillips B, Lehman E, Chinchilli V, Hawkins R, Kim J (2012). The effects of combining Web-based eHealth with telephone nurse case management for pediatric asthma control: a randomized controlled trial. J Med Internet Res.

[209] Kosse RC, Bouvy M, de Vries T, Kaptein A, Geers HC, van Dijk L, Koster E (2017). mHealth intervention to support asthma self-management in adolescents: the ADAPT study. PPA.

[210] Bender BG, Cvietusa PJ, Goodrich GK, Lowe R, Nuanes HA, Rand C, Shetterly S, Tacinas C, Vollmer WM, Wagner N, Wamboldt FS, Xu S, Magid DJ (2015). Pragmatic trial of health care technologies to improve adherence to pediatric asthma treatment: a randomized clinical trial. JAMA Pediatr.

[211] Sabhae Gangadharappa V, Nagarajan S, Weaver D (2019). Controller Medication Refill Rates in Underserved Pediatric Asthma Patients After Use of a Smartphone Application. Annals of Allergy, Asthma & Immunology.

[212] Vasbinder EC, Janssens HM, Rutten-van Mölken M, van Dijk L, de Winter BCM, de Groot RCA, Vulto AG, van den Bemt PMLA, e-MATIC Study Group (2013). e-Monitoring of Asthma Therapy to Improve Compliance in children using a real-time medication monitoring system (RTMM): the e-MATIC study protocol. BMC Med Inform Decis Mak.

[213] Cushing CC, Fedele DA, Patton SR, McQuaid EL, Smyth JM, Prabhakaran S, Gierer S, Koskela-Staples N, Ortega A, Fleming KK, Nezu AM (2019). Responsive Asthma Care for Teens (ReACT): development protocol for an adaptive mobile health intervention for adolescents with asthma. BMJ Open.

[214] Henderson BR, Flaherty CM, Floyd GC, You J, Xiao R, Bryant-Stephens TC, Miller VA, Feudtner C, Kenyon CC (2020). Tailored Medication Adherence Incentives Using mHealth for Children With High-Risk Asthma (TAICAM): Protocol for a Randomized Controlled Trial. JMIR Res Protoc.

[215] Using Technology-Assisted Stepped Care Intervention to Improve Adherence in Adolescents With Asthma (TASC). ClinicalTrials.gov.

[216] Kan K, Shaunfield S, Kanaley M, Chadha A, Boon K, Morales L, Davis M, Vojta D, Gupta R (2022). Health provider perspectives of electronic medication monitoring in outpatient asthma care: a qualitative investigation using the consolidated framework for implementation research. J Asthma.

[217] Howard S, Lang A, Sharples S, Shaw D (2017). See I told you I was taking it! - Attitudes of adolescents with asthma towards a device monitoring their inhaler use: Implications for future design. Appl Ergon.

[218] Teufel Ii R, Patel SK, Shuler AB, Andrews AL, Nichols M, Ebeling MD, Dawley E, Mueller M, Ruggiero KJ, Treiber FA (2018). Smartphones for Real-time Assessment of Adherence Behavior and Symptom Exacerbation for High-Risk Youth with Asthma: Pilot Study. JMIR Pediatr Parent.

[219] Hoch H, Kempe A, Brinton J, Szefler S (2019). Feasibility of medication monitoring sensors in high risk asthmatic children. J Asthma.

[220] Rohan JM, Drotar D, Perry AR, McDowell K, Malkin J, Kercsmar C (2013). Training health care providers to conduct adherence promotion in pediatric settings: An example with pediatric asthma. Clinical Practice in Pediatric Psychology.

[221] Makhecha S, Chan A, Pearce C, Jamalzadeh A, Fleming L (2020). Novel electronic adherence monitoring devices in children with asthma: a mixed-methods study. BMJ Open Respir Res.

[222] Graves MM, Adams CD, Portnoy JM (2006). Adherence in young children with asthma. Curr Opin Allergy Clin Immunol.

[223] Simoneau T, Sun Y, Gherlone N, Almeida S, Manice M, Hollenbach J (2019). A Prospective, Randomized, Controlled Study of Inhaler Electronic Monitoring Devices to Improve Adherence in Children with Asthma. American Thoracic Society.

[224] Dodds C, Britto M (2018). Learnings from a pragmatic pilot trial of text messaging for high-risk adolescents with asthma. Ann Allergy Asthma Immunol.

[225] Kenyon CC, Chang J, Wynter S, Fowler JC, Long J, Bryant-Stephens TC (2016). Electronic Adherence Monitoring in a High-Utilizing Pediatric Asthma Cohort: A Feasibility Study. JMIR Res Protoc.

[226] Lin NY, Ramsey RR, Miller JL, McDowell KM, Zhang N, Hommel K, Guilbert TW (2020). Telehealth delivery of adherence and medication management system improves outcomes in inner-city children with asthma. Pediatr Pulmonol.

[227] Mosnaim G, Li H, Martin M, Richardson D, Belice PJ, Avery E, Ryan N, Bender B, Powell L (2013). The impact of peer support and mp3 messaging on adherence to inhaled corticosteroids in minority adolescents with asthma: a randomized, controlled trial. J Allergy Clin Immunol Pract.

[228] Fiks AG, DuRivage N, Mayne SL, Finch S, Ross ME, Giacomini K, Suh A, McCarn B, Brandt E, Karavite D, Staton EW, Shone LP, McGoldrick V, Noonan K, Miller D, Lehmann CU, Pace WD, Grundmeier RW (2016). Adoption of a Portal for the Primary Care Management of Pediatric Asthma: A Mixed-Methods Implementation Study. J Med Internet Res.

[229] Shields MD, ALQahtani F, Rivey MP, McElnay JC (2018). Mobile direct observation of therapy (MDOT) - A rapid systematic review and pilot study in children with asthma. PLoS One.

[230] Behrooz L, Dilley M, Petty C, Huffaker M, Sheehan W, Phipatanakul W (2020). The efficacy of a novel monitoring device on asthma control in children with asthma. Ann Allergy Asthma Immunol.

[231] Kosse RC, Bouvy ML, Belitser SV, de Vries TW, van der Wal PS, Koster ES (2019). Effective Engagement of Adolescent Asthma Patients With Mobile Health-Supporting Medication Adherence. JMIR Mhealth Uhealth.

[232] Spaulding S, Devine K, Duncan C, Wilson N, Hogan M (2012). Electronic monitoring and feedback to improve adherence in pediatric asthma. J Pediatr Psychol.

[233] Gupta R, Fierstein J, Boon K, Kanaley M, Bozen A, Kan K, Vojta D, Warren C (2021). Sensor-Based Electronic Monitoring for Asthma: A Randomized Controlled Trial. Pediatrics.

[234] Otsuki M, Eakin M, Rand C, Butz A, Hsu V, Zuckerman I, Ogborn J, Bilderback A, Riekert K (2009). Adherence feedback to improve asthma outcomes among inner-city children: a randomized trial. Pediatrics.

[235] Adejumo I, Shaw DE (2018). Electronic Monitoring Devices as an Intervention in Asthma: The Story So Far. CRMR.

[236] Barrett M, Combs V, Su JG, Henderson K, Tuffli M, Louisville Collaborative Air (2018). AIR Louisville: Addressing Asthma With Technology, Crowdsourcing, Cross-Sector Collaboration, And Policy. Health Aff (Millwood).

[237] Barrett MA, Humblet O, Marcus JE, Henderson K, Smith T, Eid N, Sublett JW, Renda A, Nesbitt L, Van Sickle D, Stempel D, Sublett JL (2017). Effect of a mobile health, sensor-driven asthma management platform on asthma control. Ann Allergy Asthma Immunol.

[238] Merchant RK, Inamdar R, Quade RC (2016). Effectiveness of Population Health Management Using the Propeller Health Asthma Platform: A Randomized Clinical Trial. J Allergy Clin Immunol Pract.

[239] Shields M, Alqahtani F, Rivey M, McElnay J (2017). Using remote directly observed therapy (R-DOT) for optimising asthma therapy. European Respiratory Journal.

[240] Bynum A, Hopkins D, Thomas A, Copeland N, Irwin C (2001). The effect of telepharmacy counseling on metered-dose inhaler technique among adolescents with asthma in rural Arkansas. Telemed J E Health.

[241] Peters D, Davis S, Calvo RA, Sawyer SM, Smith L, Foster JM (2017). Young People's Preferences for an Asthma Self-Management App Highlight Psychological Needs: A Participatory Study. J Med Internet Res.

[242] Sonney J, Duffy M, Hoogerheyde L, Langhauser E, Teska D (2019). Applying Human-Centered Design to the Development of an Asthma Essentials Kit for School-Aged Children and Their Parents. J Pediatr Health Care.

[243] Schneider T, Panzera AD, Couluris M, Lindenberger J, McDermott R, Bryant CA (2016). Engaging Teens with Asthma in Designing a Patient-Centered Mobile App to Aid Disease Self-Management. Telemed J E Health.

[244] Davis S, Peters D, Calvo R, Sawyer S, Foster J, Smith L (2018). "Kiss myAsthma": Using a participatory design approach to develop a self-management app with young people with asthma. J Asthma.

[245] McWilliams A, Reeves K, Shade L, Burton E, Tapp H, Courtlandt C, Gunter A, Dulin MF (2018). Patient and Family Engagement in the Design of a Mobile Health Solution for Pediatric Asthma: Development and Feasibility Study. JMIR Mhealth Uhealth.

[246] Fiks A, Mayne S, Karavite D, DeBartolo E, Grundmeier R (2014). A shared e-decision support portal for pediatric asthma. J Ambul Care Manage.

[247] Foster JM, Peters D, Davis S, Calvo R, Sawyer S, Smith L (2017). Using A Co-Design Approach To Develop An Appealing Goal-Setting And Self-Management App For Young People With Asthma. American Thoracic Society.

[248] Jácome C, Almeida R, Pereira A, Araújo L, Correia M, Pereira M, Couto M, Lopes C, Chaves Loureiro C, Catarata M, Santos L, Ramos B, Mendes A, Pedro E, Cidrais Rodrigues J, Oliveira G, Aguiar A, Arrobas A, Costa J, Dias J, Todo Bom A, Azevedo J, Ribeiro C, Alves M, Pinto P, Neuparth N, Palhinha A, Marques J, Martins P, Trincão D, Neves A, Todo Bom F, Santos M, Branco J, Loyoza C, Costa A, Silva Neto A, Silva D, Vasconcelos M, Teixeira M, Ferreira-Magalhães M, Taborda Barata L, Carvalhal C, Santos N, Sofia Pinto C, Rodrigues Alves R, Moreira A, Morais Silva P, Fernandes R, Ferreira R, Alves C, Câmara R, Ferraz de Oliveira J, Bordalo D, Calix M, Marques A, Nunes C, Menezes F, Gomes R, Almeida Fonseca J, INSPIRERS group (2020). Asthma App Use and Interest Among Patients With Asthma: A Multicenter Study. J Investig Allergol Clin Immunol.

[249] Sage A, Roberts C, Geryk L, Sleath B, Tate D, Carpenter D (2017). A Self-Regulation Theory-Based Asthma Management Mobile App for Adolescents: A Usability Assessment. JMIR Hum Factors.

[250] Yoo W, Kim SY, Hong Y, Chih M, Shah DV, Gustafson DH (2015). Patient-clinician mobile communication: analyzing text messaging between adolescents with asthma and nurse case managers. Telemed J E Health.

[251] Stewart M, Letourneau N, Masuda JR, Anderson S, McGhan S (2013). Online support for children with asthma and allergies. J Fam Nurs.

[252] Roberts CA, Sage AJ, Geryk LL, Sleath BL, Carpenter DM (2019). Adolescent feedback on predisposing, reinforcing and enabling features in asthma self-management apps. Health Education Journal.

[253] Roberts C, Sage A, Geryk L, Sleath B, Carpenter D (2018). Adolescent Preferences and Design Recommendations for an Asthma Self-Management App: Mixed-Methods Study. JMIR Form Res.

[254] Carpenter DM, Geryk LL, Arrindell C, Tate D, Alexander DS, Sage A, Sleath BL (2015). 164. Adolescent, Caregiver, and Provider Preferences for an Asthma Self-Management App. Journal of Adolescent Health.

[255] Ramsey RR, Carmody JK, Holbein CE, Guilbert TW, Hommel KA (2019). Examination of the uses, needs, and preferences for health technology use in adolescents with asthma. J Asthma.

[256] Fedele DA, Cushing CC, Koskela-Staples N, Patton SR, McQuaid EL, Smyth JM, Prabhakaran S, Gierer S, Nezu AM (2020). Adaptive Mobile Health Intervention for Adolescents with Asthma: Iterative User-Centered Development. JMIR Mhealth Uhealth.

[257] Doyle R, Albright K, Hurley LP, Chávez C, Stowell M, Dircksen S, Havranek EP, Anderson M (2019). Patient Perspectives on a Text Messaging Program to Support Asthma Management: A Qualitative Study. Health Promot Pract.

[258] Schneider T, Panzera AD, Martinasek M, McDermott R, Couluris M, Lindenberger J, Bryant C (2016). Physicians' perceptions of mobile technology for enhancing asthma care for youth. J Child Health Care.

[259] Meischke H, Lozano P, Zhou C, Garrison MM, Christakis D (2011). Engagement in "My Child's Asthma", an interactive web-based pediatric asthma management intervention. Int J Med Inform.

[260] Yun T, Jeong H, Lee H, Arriaga R, Abowd G (2010). Assessing asthma management practices through in-home technology probes.

[261] Thomson J, Hass C, Horn I, Kleine E, Mitchell S, Gary K, Ahmed I, Hamel D, Amresh A (2017). Aspira: Employing a serious game in an mHealth app to improve asthma outcomes.

[262] Lin H, Chiang L, Wen T, Yeh K, Huang J (2014). Development of online diary and self-management system on e-Healthcare for asthmatic children in Taiwan. Comput Methods Programs Biomed.

[263] Wise M, Gustafson DH, Sorkness CA, Molfenter T, Staresinic A, Meis T, Hawkins RP, Shanovich KK, Walker NP (2007). Internet telehealth for pediatric asthma case management: integrating computerized and case manager features for tailoring a Web-based asthma education program. Health Promot Pract.

[264] Nyapathy N, Arriaga R (2019). Tracking and Reporting Asthma Data for Children.

[265] Clark M, Martin S, Svedsater H, Dale P, Jacques L (2015). Measurement properties of an asthma symptom and rescue medication use diary. J Asthma.

[266] Carpenter DM, Geryk LL, Sage A, Arrindell C, Sleath BL (2016). Exploring the theoretical pathways through which asthma app features can promote adolescent self-management. Transl Behav Med.

[267] Haze K, Lynaugh J (2013). Building patient relationships: a smartphone application supporting communication between teenagers with asthma and the RN care coordinator. Comput Inform Nurs.

[268] Odom L, Christenbery T (2016). There is an "app" for that: Designing mobile phone technology to improve asthma action plan use in adolescent patients. J Am Assoc Nurse Pract.

[269] van Bragt S, van den Bemt L, Cretier R, van Weel C, Merkus P, Schermer T (2016). PELICAN: Content evaluation of patient-centered care for children with asthma based on an online tool. Pediatr Pulmonol.

[270] Mayoral K, Garin O, Caballero-Rabasco M, Praena-Crespo M, Bercedo A, Hernandez G, Castillo J, Lizano Barrantes C, Pardo Y, Ferrer M, ARCA group (2021). Smartphone App for monitoring Asthma in children and adolescents. Qual Life Res.

[271] Nkoy FL, Stone BL, Fassl BA, Koopmeiners K, Halbern S, Kim EH, Poll J, Hales JW, Lee D, Maloney CG (2012). Development of a novel tool for engaging children and parents in asthma self-management. AMIA Annu Symp Proc.

[272] Rhee H, Allen J, Mammen J, Swift M (2014). Mobile phone-based asthma self-management aid for adolescents (mASMAA): a feasibility study. PPA.

[273] Namazova-Baranova L, Molodchenkov A, Vishneva E, Antonova EV, Smirnov V (2015). Remote monitoring of children with asthma, being treated in multidisciplinary hospital.

[274] Osuntogun A, Arriaga R (2010). Physician usage of technology and opportunities for continuous care management of pediatric asthma patients.

[275] Mantzouranis EC (2018). User Friendliness Aspects of Home Care Telematics. Methods Inf Med.

[276] Internet Telehealth for Pediatric Asthma Case Management (CHESS). ClinicalTrials.gov.

[277] Roberts C, Geryk L, Sage A, Sleath B, Tate D, Carpenter DM (2016). Adolescent, caregiver, and friend preferences for integrating social support and communication features into an asthma self-management app. J Asthma.

[278] Jayaprakash K, Stephens C, Lesnick B, Arriaga R (2019). Asthma-nauts: Apps Using Gameplay to Collect Health Metrics and Educate Kids About Asthma.

[279] Iio M, Miyaji Y, Yamamoto-Hanada K, Narita M, Nagata M, Ohya Y (2020). Beneficial Features of a mHealth Asthma App for Children and Caregivers: Qualitative Study. JMIR Mhealth Uhealth.

[280] Fedele D, Lucero R, Janicke D, Abu-Hasan M, McQuaid E, Moon J, Fidler A, Wallace-Farquharson T, Lindberg D (2019). Protocol for the Development of a Behavioral Family Lifestyle Intervention Supported by Mobile Health to Improve Weight Self-Management in Children With Asthma and Obesity. JMIR Res Protoc.

[281] Luna-Aveiga H, Medina-Moreira J, Apolinario-Arzube O, Paredes-Valverde M, Lagos-Ortiz K, Valencia-García R (2018). Astmapp: A platform for asthma self-management. Journal of Universal Computer Science.

[282] Geryk LL, Roberts CA, Sage AJ, Coyne-Beasley T, Sleath BL, Carpenter DM (2016). Parent and Clinician Preferences for an Asthma App to Promote Adolescent Self-Management: A Formative Study. JMIR Res Protoc.

[283] Elias P, Rajan NO, McArthur K, Dacso CC (2013). InSpire to Promote Lung Assessment in Youth: Evolving the Self-Management Paradigms of Young People With Asthma. Med 2 0.

[284] Panzera AD, Schneider TK, Martinasek MP, Lindenberger JH, Couluris M, Bryant CA, McDermott RJ (2013). Adolescent asthma self-management: patient and parent-caregiver perspectives on using social media to improve care. J Sch Health.

[285] Licari A, Ferrante G, Marseglia Md G, Corsello Md G, La Grutta S (2019). What Is the Impact of Innovative Electronic Health Interventions in Improving Treatment Adherence in Asthma? The Pediatric Perspective. J Allergy Clin Immunol Pract.

[286] AIM2ACT: A Mobile Health Tool to Help Adolescents Self-Manage Asthma (AIM2ACT). ClinicalTrials.gov.

[287] Bonini M (2017). Electronic health (e-Health): emerging role in asthma. Curr Opin Pulm Med.

[288] Perry TT, Margiotta CA (2020). Implementing Telehealth in Pediatric Asthma. Pediatr Clin North Am.

[289] Moeinedin F, Moineddin R, Jadad AR, Hamid JS, To T, Beyene J (2009). Application of biomedical informatics to chronic pediatric diseases: a systematic review. BMC Med Inform Decis Mak.

[290] Bruzzese J, George M, Liu J, Evans D, Naar S, DeRosier M, Thomas J (2021). The Development and Preliminary Impact of CAMP Air: A Web-based Asthma Intervention to Improve Asthma Among Adolescents. Patient Educ Couns.

[291] Kew K, Cates C (2016). Remote versus face-to-face check-ups for asthma. Cochrane Database Syst Rev.

[292] Huss K, Winkelstein M, Nanda J, Naumann P, Sloand E, Huss R (2003). Computer game for inner-city children does not improve asthma outcomes. Journal of Pediatric Health Care.

[293] Willems D, Joore M, Hendriks J, Nieman F, Severens J, Wouters E (2008). The effectiveness of nurse-led telemonitoring of asthma: results of a randomized controlled trial. J Eval Clin Pract.

[294] Joseph CLM, Mahajan P, Stokes-Buzzelli S, Johnson DA, Duffy E, Williams R, Zhang T, Ownby DR, Considine S, Lu M (2018). Pilot study of a randomized trial to evaluate a Web-based intervention targeting adolescents presenting to the emergency department with acute asthma. Pilot Feasibility Stud.

[295] Murphy JA, Heisser JM, Montgomery M (2019). Evidence-Based Review of Smartphone Versus Paper Asthma Action Plans on Asthma Control. J Pharm Technol.

[296] Lu M, Zhang T, Ownby D, Zoratti E, Johnson D, William R, Miree C, Joseph C (2019). Phase II trial of web-based tailored asthma management intervention in adolescents at clinics. Contemp Clin Trials.

[297] Joseph CL, Ownby DR, Havstad SL, Saltzgaber J, Considine S, Johnson D, Peterson E, Alexander G, Lu M, Gibson-Scipio W, Johnson CC, Research team members (2013). Evaluation of a web-based asthma management intervention program for urban teenagers: reaching the hard to reach. J Adolesc Health.

[298] Lv S, Ye X, Wang Z, Xia W, Qi Y, Wang W, Chen Y, Cai X, Qian X (2019). A randomized controlled trial of a mobile application-assisted nurse-led model used to improve treatment outcomes in children with asthma. J Adv Nurs.

[299] Vallabhan M, Jimenez E, McCauley G, Willyard H, Kong A (2021). Formative Evaluation for Implementation of a Low Literacy Pictorial Asthma Action Plan Delivered via Telehealth Improves Asthma Control. Am J Med Qual.

[300] Burbank AJ, Lewis SD, Hewes M, Schellhase DE, Rettiganti M, Hall-Barrow J, Bylander LA, Brown RH, Perry TT (2015). Mobile-based asthma action plans for adolescents. J Asthma.

[301] Rikkers-Mutsaerts N, Beerthuizen T, Winters A, Bakker M, Sont J (2014). Internet-based self-management in adolescents with asthma: The role of education, monitoring and symptom perception. European Respiratory Journal.

[302] Chan D, Callahan C, Hatch-Pigott V, Lawless A, Proffitt H, Manning N, Schweikert M, Malone F (2007). Internet-based home monitoring and education of children with asthma is comparable to ideal office-based care: results of a 1-year asthma in-home monitoring trial. Pediatrics.

[303] Faraji S, Valizadeh S, Sharifi A, Shabazi S, Ghojazadeh M (2020). The effectiveness of telegram-based virtual education versus in-person education on the quality of life in adolescents with moderate-to-severe asthma: A pilot randomized controlled trial. Nurs Open.

[304] Hashi S, Tsukasaki K, Nakamura T, Kyota K, Itatani T (2019). Effects of maintaining web-based diaries by caregivers on adherence to care regimens in preschoolers with asthma. J Spec Pediatr Nurs.

[305] Fiks A, Mayne S, Karavite D, Suh A, O'Hara R, Localio A, Ross M, Grundmeier R (2015). Parent-reported outcomes of a shared decision-making portal in asthma: a practice-based RCT. Pediatrics.

[306] Stewart M, Letourneau N, Masuda JR, Anderson S, McGhan S (2013). Impacts of online peer support for children with asthma and allergies: It just helps you every time you can't breathe well". J Pediatr Nurs.

[307] Tan R, Cvetkovski B, Kritikos V, O'Hehir R, Lourenço O, Bousquet J, Bosnic-Anticevich S (2020). Identifying an effective mobile health application for the self-management of allergic rhinitis and asthma in Australia. J Asthma.

[308] Fisher-Owens SA, Boddupalli G, Thyne SM (2011). Telephone case management for asthma: an acceptable and effective intervention within a diverse pediatric population. J Asthma.

[309] Stukus D, Farooqui N, Strothman K, Ryan K, Zhao S, Stevens J, Cohen DM (2018). Real-world evaluation of a mobile health application in children with asthma. Ann Allergy Asthma Immunol.

[310] Coughey K, Klein G, West C, Diamond JJ, Santana A, McCarville E, Rosenthal MP (2010). The Child Asthma Link Line: a coalition-initiated, telephone-based, care coordination intervention for childhood asthma. J Asthma.

[311] Xu C, Jackson M, Scuffham PA, Wootton R, Simpson P, Whitty J, Wolfe R, Wainwright CE (2010). A randomized controlled trial of an interactive voice response telephone system and specialist nurse support for childhood asthma management. J Asthma.

[312] Kassem A, Hamad M, El-Moucary C, Neghawi E, Bou JG, Merhej C (2013). Asthma Care Apps.

[313] Kassem A, Hamad M, El Moucary C (2015). A smart spirometry device for asthma diagnosis.

[314] Gupta S, Chang P, Anyigbo N, Sabharwal A (2011). mobileSpiro: portable open-interface spirometry for Android. WH '11: Proceedings of the 2nd Conference on Wireless Health.

[315] Shenoy N, Nazeran H (2005). A PDA-based Network for Telemonitoring Asthma Triggering Gases in the El Paso School Districts of the US - Mexico Border Region.

[316] Lee C, Chen JC, Tseng VS (2011). A novel data mining mechanism considering bio-signal and environmental data with applications on asthma monitoring. Comput Methods Programs Biomed.

[317] Lee H, Panont W, Plattenburg B, de la Croix J, Patharachalam D, Abowd G (2010). Asthmon: empowering asthmatic children's self-management with a virtual pet. CHI EA '10: CHI '10 Extended Abstracts on Human Factors in Computing Systems.

[318] Aleksovska-Stojkovska L, Loskovska S (2011). Architectural and data model of clinical decision support system for managing asthma in school-aged children.

[319] Zaharudin S, Kazemi M, Malarvili M (2014). Designing a respiratory CO2 measurement device for home monitoring of asthma severity.

[320] Ginsberg D (2020). An Unidentified Monster in the Bed – Assessing Nocturnal Asthma in Children. MJM.

[321] Johnson M, Hudgens E, Williams R, Andrews G, Neas L, Gallagher J, Ozkaynak H (2009). A participant-based approach to indoor/outdoor air monitoring in community health studies. J Expo Sci Environ Epidemiol.

[322] Luo G, Stone BL, Fassl B, Maloney CG, Gesteland PH, Yerram SR, Nkoy FL (2015). Predicting asthma control deterioration in children. BMC Med Inform Decis Mak.

[323] Frima E, Theodorakopoulos I, Gidaris D, Karantaglis N, Chatziparasidis G, Plotas P, Anthracopoulos M, Fouzas S (2020). Lung Function Variability in Children and Adolescents With and Without Asthma (LUV Study): Protocol for a Prospective, Nonrandomized, Clinical Trial. JMIR Res Protoc.

[324] Seppä V, Gracia-Tabuenca J, Kotaniemi-Syrjänen A, Malmström K, Hult A, Pelkonen AS, Mäkelä M, Viik J, Malmberg LP (2020). Expiratory variability index is associated with asthma risk, wheeze and lung function in infants with recurrent respiratory symptoms. ERJ Open Res.

[325] Stewart AC, Gannon KN, Beresford F, Fleming L (2018). Adolescent and caregivers' experiences of electronic adherence assessment in paediatric problematic severe asthma. J Child Health Care.

[326] Dong Q, Li B, Downen RS, Tran N, Chorvinsky E, Pillai DK, Zaghloul ME, Li Z (2020). A Cloud-connected NO and Ozone Sensor System for Personalized Pediatric Asthma Research and Management. IEEE Sens J.

[327] Freeman B, Mayne S, Localio AR, Luberti A, Zorc JJ, Fiks AG (2017). Using Video from Mobile Phones to Improve Pediatric Phone Triage in an Underserved Population. Telemed J E Health.

[328] Johnson M, Macneill M, Grgicak-Mannion A, Nethery E, Xu X, Dales R, Rasmussen P, Wheeler A (2013). Development of temporally refined land-use regression models predicting daily household-level air pollution in a panel study of lung function among asthmatic children. J Expo Sci Environ Epidemiol.

[329] Floro JN, Dunton GF, Delfino RJ (2009). Assessing physical activity in children with asthma: convergent validity between accelerometer and electronic diary data. Res Q Exerc Sport.

[330] Rietveld S, Everaerd W (2000). Perceptions of asthma by adolescents at home. Chest.

[331] Brouwer AFJ, Roorda RJ, Duiverman EJ, Brand PLP (2008). Reference values for peak flow and FEV1 variation in healthy schoolchildren using home spirometry. Eur Respir J.

[332] Holguin F, Flores S, Ross Z, Cortez M, Molina M, Molina L, Rincon C, Jerrett M, Berhane K, Granados A, Romieu I (2007). Traffic-related Exposures, Airway Function, Inflammation, and Respiratory Symptoms in Children. Am J Respir Crit Care Med.

[333] Gauvin S, Reungoat P, Cassadou S, Déchenaux J, Momas I, Just J, Zmirou D (2002). Contribution of indoor and outdoor environments to PM2.5 personal exposure of children--VESTA study. Sci Total Environ.

[334] Rich M, Lamola S, Amory C, Schneider L (2000). Asthma in life context: Video Intervention/Prevention Assessment (VIA). Pediatrics.

[335] Hetzel MR, Clark TJ (1980). Comparison of normal and asthmatic circadian rhythms in peak expiratory flow rate. Thorax.

[336] Spira-Cohen A, Chen LC, Kendall M, Sheesley R, Thurston GD (2010). Personal exposures to traffic-related particle pollution among children with asthma in the South Bronx, NY. J Expo Sci Environ Epidemiol.

[337] Williams LK, Peterson EL, Wells K, Campbell J, Wang M, Chowdhry VK, Walsh M, Enberg R, Lanfear DE, Pladevall M (2010). A cluster-randomized trial to provide clinicians inhaled corticosteroid adherence information for their patients with asthma. J Allergy Clin Immunol.

[338] Wallace LA, Mitchell H, O'Connor GT, Neas L, Lippmann M, Kattan M, Koenig J, Stout JW, Vaughn BJ, Wallace D, Walter M, Adams K, Liu LS, Inner-City Asthma Study (2003). Particle concentrations in inner-city homes of children with asthma: the effect of smoking, cooking, and outdoor pollution. Environ Health Perspect.

[339] Enright PL, Sherrill DL, Lebowitz MD (1995). Ambulatory monitoring of peak expiratory flow. Reproducibility and quality control. Chest.

[340] Kamps JL, Rapoff MA, Roberts MC, Varela RE, Barnard M, Olson N (2008). Improving Adherence to Inhaled Corticosteroids in Children With Asthma: A Pilot of a Randomized Clinical Trial. Children's Health Care.

[341] Eisenberg SR, Jelalian E, Farrow M, Kopel SJ, Vehse N, Mitchell P, Dunsiger S, Koinis-Mitchell D (2020). Perceptions of Asthma and Exercise, and Associations With Weight Status and Asthma Morbidity in Urban Children. Acad Pediatr.

[342] Wong A, Hardaker K, Field P, Huvanandana J, King GG, Reddel H, Selvadurai H, Thamrin C, Robinson PD (2019). Home-based Forced Oscillation Technique Day-to-Day Variability in Pediatric Asthma. Am J Respir Crit Care Med.

[343] Nimmagadda S (2018). Electronic Monitoring of Adherence to Inhaled Corticosteroids: An Essential Tool in Identifying Severe Asthma in Children. Pediatrics.

[344] Wu C, Delfino R, Floro J, Quintana P, Samimi B, Kleinman M, Allen R, Sally Liu L (2005). Exposure assessment and modeling of particulate matter for asthmatic children using personal nephelometers. Atmospheric Environment.

[345] Belanger K, Holford TR, Gent JF, Hill ME, Kezik JM, Leaderer BP (2013). Household Levels of Nitrogen Dioxide and Pediatric Asthma Severity. Epidemiology.

[346] Gore RB, Curbishley L, Truman N, Hadley E, Woodcock A, Langley SJ, Custovic A (2006). Intranasal air sampling in homes: relationships among reservoir allergen concentrations and asthma severity. J Allergy Clin Immunol.

[347] Stahlman J, Alghamdi K, Salmun L, Borras I, Bailey E, Schneider L (2006). Adherence with a Hand-Held Electronic Device versus Conventional Peak Expiratory Flow Rate Monitoring in Children with Asthma. Pediatric Asthma, Allergy & Immunology.

[348] van Bragt S, van den Bemt L, Thoonen B, Jacobs J, Merkus P, Schermer T (2014). Validity, reliability and discriminative capacity of an electronic quality of life instrument (Pelican) for childhood asthma in the Netherlands. Qual Life Res.

[349] Goldberg S, Springer C, Avital A, Godfrey S, Bar-Yishay E (2001). Can peak expiratory flow measurements estimate small airway function in asthmatic children?. Chest.

[350] Koenig JQ, Jansen K, Mar TF, Lumley T, Kaufman J, Trenga CA, Sullivan J, Liu LS, Shapiro GG, Larson TV (2003). Measurement of offline exhaled nitric oxide in a study of community exposure to air pollution. Environ Health Perspect.

[351] Taplin PS, Creer TL (1978). A procedure for using peak expiratory flow rate data to increase the predictability of asthma episodes. J Asthma Res.

[352] Kannisto S, Korppi M (1999). Bronchial Lability Index in the Diagnosis of Asthma in Children. Pediatric Asthma, Allergy & Immunology.

[353] Kerwin E, Yiu G, Hickey L, Small C (2017). Analysis of the relationship between handheld and clinic-based spirometry measurements in a randomized, double-blind, placebo-controlled study of beclomethasone dipropionate via breath-actuated inhaler for persistent asthma. Am J Respir Crit Care Med.

[354] Hernández-Cadena L, Holguin F, Barraza-Villarreal A, Del Río-Navarro B, Sienra-Monge JJ, Romieu I (2009). Increased levels of outdoor air pollutants are associated with reduced bronchodilation in children with asthma. Chest.

[355] Brand PL, Mäkelä MJ, Szefler SJ, Frischer T, Price D, ERS Task Force Monitoring Asthma in Children (2015). Monitoring asthma in childhood: symptoms, exacerbations and quality of life. Eur Respir Rev.

[356] Nkoy F, Wilkins V, Fassl B, Sheng X, Stone B (2020). Impact of a self-monitoring application on pediatric asthma disparities. Int J Med Inform.

[357] Burkhart PV, Rayens MK, Oakley MG, Abshire DA, Zhang M (2007). Testing an intervention to promote children's adherence to asthma self-management. J Nurs Scholarsh.

[358] Blaiss M (2018). Asthma mobile applications: Are they ready for prime time?. Ann Allergy Asthma Immunol.

[359] Letourneau N, Stewart M, Masuda JR, Anderson S, Cicutto L, McGhan S, Watt S (2012). Impact of online support for youth with asthma and allergies: pilot study. J Pediatr Nurs.

[360] Kew K, Cates C (2016). Home telemonitoring and remote feedback between clinic visits for asthma. Cochrane Database Syst Rev.

[361] Bush A, Eber E (2008). The value of FeNO measurement in asthma management: the motion for Yes, it's NO--or, the wrong end of the Stick!. Paediatr Respir Rev.

[362] Bonini M, Usmani O (2018). Novel methods for device and adherence monitoring in asthma. Curr Opin Pulm Med.

[363] Blanchet KD (2009). Remote monitoring of asthma. Telemed J E Health.

[364] Baptist AP, Islam N, Joseph CL (2016). Technology-Based Interventions for Asthma-Can They Help Decrease Health Disparities?. J Allergy Clin Immunol Pract.

[365] Yun T, Jeong H, Hill T, Lesnick B, Brown R, Abowd G, Arriaga R (2012). Using SMS to provide continuous assessment and improve health outcomes for children with asthma. IHI '12: Proceedings of the 2nd ACM SIGHIT International Health Informatics Symposium.

[366] Plymat KR, Bunn CL (1985). Monitoring asthma with a Mini-Wright Peak Flow Meter. Nurse Pract.

[367] Guendelman S, Meade K, Chen YQ, Benson M (2004). Asthma Control and Hospitalizations Among Inner-City Children: Results of a Randomized Trial. telemed j e health.

[368] Pinnock H, Slack R, Pagliari C, Price D, Sheikh A (2007). Understanding the potential role of mobile phone-based monitoring on asthma self-management: qualitative study. Clin Exp Allergy.

[369] Lombardi C, Bonini M, Passalacqua G (2018). The role of mobile apps in allergic respiratory diseases: an Italian multicentre survey report. Eur Ann Allergy Clin Immunol.

[370] Anderson W, Gondalia R, Hoch H, Kaye L, Szefler S, Stempel D (2019). Screening for inhalation technique errors with electronic medication monitors. J Allergy Clin Immunol Pract.

[371] Bender BG (2018). Technology Interventions for Nonadherence: New Approaches to an Old Problem. J Allergy Clin Immunol Pract.

[372] Liptzin D, Szefler S (2016). Evolution of Asthma Self-Management Programs in Adolescents: From the Crisis Plan to Facebook. J Pediatr.

[373] Zhou Y, Lu Y, Zhu H, Zhang Y, Li Y, Yu Q (2018). Short-term effect of a smart nebulizing device on adherence to inhaled corticosteroid therapy in Asthma Predictive Index-positive wheezing children. PPA.

[374] Szefler SJ (2015). Monitoring and adherence in asthma management. The Lancet Respiratory Medicine.

[375] Morton RW, Everard ML, Elphick HE (2014). Adherence in childhood asthma: the elephant in the room. Arch Dis Child.

[376] Willems DC, Joore MA, Hendriks JJ, Wouters EF, Severens JL (2007). Cost-effectiveness of a nurse-led telemonitoring intervention based on peak expiratory flow measurements in asthmatics: results of a randomised controlled trial. Cost Eff Resour Alloc.

[377] Rand CS, Wise RA (1994). Measuring adherence to asthma medication regimens. Am J Respir Crit Care Med.

[378] Ramsey R, Guilbert T (2021). Exciting Era of Sensor-Based Electronic Monitoring of Adherence in Pediatric Asthma. Pediatrics.

[379] Brooks EA, Massanari M, Hanania NA, Weiner DJ (2019). Cost-effectiveness of fractional exhaled nitric oxide (FeNO) measurement in predicting response to omalizumab in asthma. CEOR.

[380] Harnan SE, Tappenden P, Essat M, Gomersall T, Minton J, Wong R, Pavord I, Everard M, Lawson R (2015). Measurement of exhaled nitric oxide concentration in asthma: a systematic review and economic evaluation of NIOX MINO, NIOX VERO and NObreath. Health Technol Assess.

[381] Borries T, Dunbar A, Bhukhen A, Rismany J, Kilham J, Feinn R, Meehan T (2019). The impact of telemedicine on patient self-management processes and clinical outcomes for patients with Types I or II Diabetes Mellitus in the United States: A scoping review. Diabetes Metab Syndr.

[382] Triberti S, Savioni L, Sebri V, Pravettoni G (2019). eHealth for improving quality of life in breast cancer patients: A systematic review. Cancer Treat Rev.

[383] Stevens WJM, van der Sande R, Beijer LJ, Gerritsen MG, Assendelft WJ (2019). eHealth Apps Replacing or Complementing Health Care Contacts: Scoping Review on Adverse Effects. J Med Internet Res.

[384] Pakkasela J, Ilmarinen P, Honkamäki J, Tuomisto L, Andersén H, Piirilä P, Hisinger-Mölkänen H, Sovijärvi A, Backman H, Lundbäck B, Rönmark E, Kankaanranta H, Lehtimäki L (2020). Age-specific incidence of allergic and non-allergic asthma. BMC Pulm Med.

[385] Lim H, Kwon H, Lim J, Choi JH, Ha M, Hwang S, Choi W (2016). Short-term Effect of Fine Particulate Matter on Children's Hospital Admissions and Emergency Department Visits for Asthma: A Systematic Review and Meta-analysis. J Prev Med Public Health.

[386] Zheng X, Ding H, Jiang L, Chen S, Zheng J, Qiu M, Zhou Y, Chen Q, Guan W (2015). Association between Air Pollutants and Asthma Emergency Room Visits and Hospital Admissions in Time Series Studies: A Systematic Review and Meta-Analysis. PLoS One.

[387] Mazenq J, Dubus J, Gaudart J, Charpin D, Viudes G, Noel G (2017). City housing atmospheric pollutant impact on emergency visit for asthma: A classification and regression tree approach. Respir Med.

[388] Janssens T, Ritz T (2013). Perceived triggers of asthma: key to symptom perception and management. Clin Exp Allergy.

[389] Guarnieri M, Balmes JR (2014). Outdoor air pollution and asthma. The Lancet.

[390] Vrijlandt EJLE, de Jongste JC (2009). Astma bij kinderen. KIND.

[391] Vahlkvist S, Pedersen S (2009). Fitness, daily activity and body composition in children with newly diagnosed, untreated asthma. Allergy.

[392] Sousa AW, Cabral ALB, Martins MA, Carvalho CRF (2014). Daily physical activity in asthmatic children with distinct severities. J Asthma.

[393] Cassim R, Koplin JJ, Dharmage SC, Senaratna BCV, Lodge CJ, Lowe AJ, Russell MA (2016). The difference in amount of physical activity performed by children with and without asthma: A systematic review and meta-analysis. J Asthma.

[394] Anthracopoulos MB, Fouzas S, Papadopoulos M, Antonogeorgos G, Papadimitriou A, Panagiotakos DB, Nicolaidou P, Priftis KN (2012). Physical activity and exercise-induced bronchoconstriction in Greek schoolchildren. Pediatr Pulmonol.

[395] Banasiak NC (2016). Understanding the Relationship Between Asthma and Sleep in the Pediatric Population. J Pediatr Health Care.

[396] Gregory AM, Sadeh A (2012). Sleep, emotional and behavioral difficulties in children and adolescents. Sleep Med Rev.

[397] Fadzil A (2021). Factors Affecting the Quality of Sleep in Children. Children (Basel).

[398] Seppä VP, Hult A, Gracia-Tabuenca J, Paassilta M, Viik J, Plavec D, Karjalainen J (2019). Airway obstruction is associated with reduced variability in specific parts of the tidal breathing flow-volume curve in young children. ERJ Open Res.

[399] Juniper E, O'Byrne PM, Guyatt G, Ferrie P, King D (1999). Development and validation of a questionnaire to measure asthma control. Eur Respir J.

[400] Nathan RA, Sorkness CA, Kosinski M, Schatz M, Li JT, Marcus P, Murray JJ, Pendergraft TB (2004). Development of the asthma control test: a survey for assessing asthma control. J Allergy Clin Immunol.

[401] Liu AH, Zeiger R, Sorkness C, Mahr T, Ostrom N, Burgess S, Rosenzweig JC, Manjunath R (2007). Development and cross-sectional validation of the Childhood Asthma Control Test. J Allergy Clin Immunol.

[402] Rapino D, Attanasi M, Consilvio NP, Scaparrotta A, Cingolani A, Cerasa M, Mohn A, Di Pillo S, Chiarelli F (2013). Evaluation of association between airway hyperresponsiveness, asthma control test, and asthma therapy assessment questionnaire in asthmatic children. Multidiscip Respir Med.

[403] Madhuban AA, Driessen JM, Brusse-Keizer MG, van Aalderen WM, de Jongh FH, Thio BJ (2011). Association of the asthma control questionnaire with exercise-induced bronchoconstriction. J Asthma.

[404] Still L, Dolen WK (2016). The Perception of Asthma Severity in Children. Curr Allergy Asthma Rep.

[405] Koster ES, Philbert D, de Vries TW, van Dijk L, Bouvy ML (2015). "I just forget to take it": asthma self-management needs and preferences in adolescents. J Asthma.

[406] van Buul A, Kasteleyn M, Arends J, Shi T, Kelly D, Chavannes N, Meijer E (2020). eHealth only interventions and blended interventions to support self-management in adolescents with asthma: A systematic review. Clinical eHealth.

[407] Schoultz K, Svensson A, Emilsson M (2022). Nurses' experiences of using AsthmaTuner - an eHealth self-management system for healthcare of patients with asthma. Digit Health.

[408] Ramsey WA, Heidelberg RE, Gilbert AM, Heneghan MB, Badawy SM, Alberts NM (2020). eHealth and mHealth interventions in pediatric cancer: A systematic review of interventions across the cancer continuum. Psychooncology.

[409] Lau N, Waldbaum S, Parigoris R, O'Daffer A, Walsh C, Colt SF, Yi-Frazier JP, Palermo TM, McCauley E, Rosenberg AR (2020). eHealth and mHealth Psychosocial Interventions for Youths With Chronic Illnesses: Systematic Review. JMIR Pediatr Parent.

[410] Eysenbach G (2004). Tackling publication bias and selective reporting in health informatics research: register your eHealth trials in the International eHealth Studies Registry. J Med Internet Res.

[411] Baker T, Gustafson D, Shaw B, Hawkins R, Pingree S, Roberts L, Strecher V (2010). Relevance of CONSORT reporting criteria for research on eHealth interventions. Patient Educ Couns.

[412] Peroni D, Bodini A, Loiacono A, Paida G, Tenero L, Piacentini G (2009). Bioimpedance monitoring of airway inflammation in asthmatic allergic children. Allergol Immunopathol (Madr).

[413] Coutier L, Varechova S, Demoulin B, Bonabel C, Roman-Amat C, Tuan TL, Ioan I, Schweitzer C, Marchal F (2014). Specific airway resistance in children: panting or tidal breathing?. Pediatr Pulmonol.

[414] Starczewska-Dymek L, Bożek A, Dymek T (2019). Application of the Forced Oscillation Technique in Diagnosing and Monitoring of Asthma in Preschool Children. Advances in Respiratory Medicine.

[415] Abdel-Aziz M, Brinkman P, Vijverberg S, Neerincx A, de Vries R, Dagelet Y, Riley J, Hashimoto S, Montuschi P, Chung K, Djukanovic R, Fleming L, Murray C, Frey U, Bush A, Singer F, Hedlin G, Roberts G, Dahlén S, Adcock I, Fowler S, Knipping K, Sterk P, Kraneveld A, Maitland-van der Zee A, U-BIOPRED Study Group, Amsterdam UMC Breath Research Group (2020). eNose breath prints as a surrogate biomarker for classifying patients with asthma by atopy. J Allergy Clin Immunol.

[416] Keijzer P, Thio B, de Jongh F, Driessen J (2019). Assessment of Asthma in Children Using Electromyography. American Thoracic Society.

[417] Brandwein A, Patel K, Kline M, Silver P, Gangadharan S (2018). Using Pleth Variability as a Triage Tool for Children With Obstructive Airway Disease in a Pediatric Emergency Department. Pediatric Emergency Care.

[418] Uong A, Brandwein A, Crilly C, York T, Avarello J, Gangadharan S (2018). Pleth Variability Index to Assess Course of Illness in Children with Asthma. J Emerg Med.

[419] Feitosa LADS, de Britto MCA, Aliverti A, Noronha JB, de Andrade AD (2019). Accuracy of optoelectronic plethysmography in childhood exercise-induced asthma. J Asthma.

[420] Anderson G (2008). Endotyping asthma: new insights into key pathogenic mechanisms in a complex, heterogeneous disease. The Lancet.

[421] Chung HL (2011). Asthma in childhood: a complex, heterogeneous disease. Korean J Pediatr.

[422] Kocsis O, Lalos A, Arvanitis G, Moustakas K (2019). Multi-model Short-term Prediction Schema for mHealth Empowering Asthma Self-management. Electronic Notes in Theoretical Computer Science.

[423] Exarchos K, Beltsiou M, Votti C, Kostikas K (2020). Artificial intelligence techniques in asthma: a systematic review and critical appraisal of the existing literature. Eur Respir J.

[424] Beller E, Chen J, Wang U, Glasziou P (2013). Are systematic reviews up-to-date at the time of publication?. Syst Rev.

[425] Tricco A, Lillie E, Zarin W, O'Brien K, Colquhoun H, Kastner M, Levac D, Ng C, Sharpe J, Wilson K, Kenny M, Warren R, Wilson C, Stelfox H, Straus S (2016). A scoping review on the conduct and reporting of scoping reviews. BMC Med Res Methodol.

