# Supplementary material for: eHealth Technologies for Monitoring Pediatric Asthma at Home: Scoping Review
Source: J Med Internet Res. 2023 Jul 21;25:e45896. doi: 10.2196/45896 (PMC10403763; doi:10.2196/45896)
Supplement: Multimedia Appendix 1 [file jmir_v25i1e45896_app1.docx]

**Multimedia Appendix 1.** PubMed search strategy.

**PUBMED:**

#1: asthma[MeSH Terms] OR asthma*[tiab] OR bronchoconstriction[tiab] OR bronchospas*[tiab] OR bronchial constriction[tiab]

AND

#2: pediatrics[MeSH Terms] OR pediatric*[tiab] OR paediatric*[tiab] OR child[MeSH Terms] OR child*[tiab] OR adolescent[MeSH Terms] OR adolescen*[tiab] OR youth*[tiab] OR teenage*[tiab]

AND

Wearable electronic devices[Mesh Terms] OR wearable*[tiab] OR computers, handheld[Mesh Terms] OR handheld computer*[tiab] OR hand-held computer*[tiab] OR smartphone*[tiab] OR electronics medical[Mesh Terms] OR medical electronic*[tiab] OR tracker*[tiab] OR telemetry[Mesh Terms] OR telemetr*[tiab] OR actigraphy[Mesh Terms] OR actigraph*[tiab] OR (home[tiab] AND sensor[tiab]) OR telemedicine[Mesh Terms] OR telemedicine[tiab] OR eHealth[tiab] OR e-Health[tiab] OR mHealth[tiab] OR m-Health[tiab] OR telemanagement[tiab] OR tele-management[tiab] OR (mobile[tiab] AND health[tiab]) OR (electronic[tiab] AND health[tiab]) OR telehealth[tiab] OR mobile applications[Mesh Terms] OR (mobile[tiab] AND application*[tiab]) OR internet based interventions[Mesh Terms] OR (internet based[tiab] AND intervention*[tiab]) OR (web based[tiab] AND intervention*[tiab]) OR platform*[tiab] OR (online[tiab] AND intervention*[tiab]) OR (digital*[tiab] AND intervention*[tiab]) OR (online[tiab] AND monitor*[tiab]) OR (digital[tiab] AND monitor*[tiab]) OR (mobile[tiab] AND (application*[tiab] OR app[tiab])) OR computer-assisted[tiab] OR monitoring, ambulatory[Mesh Terms] OR (ambula*[tiab] AND monitor*[tiab]) OR (home[tiab] AND monitor*[tiab]) OR (home[tiab] AND electronic*[tiab]) OR (home[tiab] AND diagnos*[tiab])
