# Supplementary material for: eHealth Technologies for Monitoring Pediatric Asthma at Home: Scoping Review
Source: J Med Internet Res. 2023 Jul 21;25:e45896. doi: 10.2196/45896 (PMC10403763; doi:10.2196/45896)
Supplement: Multimedia Appendix 2 [file jmir_v25i1e45896_app2.docx]

**Multimedia Appendix 2.** Included studies.

|  | **Title** | **Year** | **Authors** | ***Airquality*** | ***Inflammation markers*** | ***Lung function*** | ***Other phys. measurements*** | ***Activity*** | ***Sleep*** | ***Questionnaires*** | ***Medication monitoring*** | ***Audiovisual*** | ***Digital environment*** |
| --- | --- | --- | --- | --- | --- | --- | --- | --- | --- | --- | --- | --- | --- |
| ***PHASE: DEVELOPMENT*** | |  |  |  |  |  |  |  |  |  |  |  |  |
| [75] | Automatic Quality Assessment of Smart Device Microphone Spirometry | 2018 | Pinho et al. | 0 | 0 | 1 | 0 | 0 | 0 | 0 | 0 | 0 | 1 |
| [251] | Online support for children with asthma and allergies. | 2013 | Stewart et al. | 0 | 0 | 0 | 0 | 0 | 0 | 0 | 0 | 0 | 1 |
| [275] | User friendliness aspects of home care telematics. | 2002 | Mantzouranis et al. | 0 | 0 | 0 | 0 | 0 | 0 | 0 | 0 | 0 | 1 |
| [129] | Automatic wheeze detection using histograms of sample entropy | 2008 | Jin et al. | 0 | 0 | 0 | 0 | 0 | 0 | 0 | 0 | 1 | 0 |
| [32] | A wireless body sensor network for the prevention and management of asthma | 2009 | Seto et al. | 1 | 0 | 0 | 0 | 1 | 0 | 0 | 0 | 0 | 1 |
| [312] | Asthma Care Apps | 2013 | Kassem et al. | 0 | 0 | 1 | 0 | 0 | 0 | 0 | 0 | 0 | 1 |
| [170] | Understanding clinicians' attitudes toward a mobile health strategy to childhood asthma management: A qualitative study. | 2017 | Hollenbach et al. | 0 | 0 | 0 | 0 | 0 | 0 | 0 | 1 | 0 | 0 |
| [249] | A Self-Regulation Theory-Based Asthma Management Mobile App for Adolescents: A Usability Assessment. | 2017 | Sage et al. | 0 | 0 | 0 | 0 | 0 | 0 | 0 | 0 | 0 | 1 |
| [243] | Engaging Teens with Asthma in Designing a Patient-Centered Mobile App to Aid Disease Self-Management. | 2016 | Schneider et al. | 0 | 0 | 0 | 0 | 0 | 0 | 0 | 0 | 0 | 1 |
| [137] | A new device for ambulatory cough recording. | 1994 | Munyard et al. | 0 | 0 | 0 | 1 | 0 | 0 | 0 | 0 | 1 | 0 |
| [148] | Nocturnal Heart Rate Variability Spectrum Characterization in Preschool Children With Asthmatic Symptoms | 2018 | Milagro et al. | 0 | 0 | 0 | 1 | 0 | 0 | 0 | 0 | 0 | 0 |
| [277] | Adolescent, caregiver, and friend preferences for integrating social support and communication features into an asthma self-management app. | 2016 | Roberts et al. | 0 | 0 | 0 | 0 | 0 | 0 | 0 | 0 | 0 | 1 |
| [313] | A smart spirometry device for asthma diagnosis. | 2015 | Kassem et al. | 0 | 0 | 1 | 0 | 0 | 0 | 0 | 0 | 0 | 0 |
| [273] | Remote Monitoring of Children with Asthma , Being Treated in Multidisciplinary Hospital | 2015 | Namazova-baranova et al. | 0 | 0 | 0 | 0 | 0 | 0 | 0 | 0 | 0 | 1 |
| [241] | Young People's Preferences for an Asthma Self-Management App Highlight Psychological Needs: A Participatory Study. | 2017 | Peters et al. | 0 | 0 | 0 | 0 | 0 | 0 | 0 | 0 | 0 | 1 |
| [76] | SpiroPlay, a Suite of Breathing Games for Spirometry by Kids & Experts | 2020 | van Delden et al. | 0 | 0 | 1 | 0 | 0 | 0 | 0 | 0 | 0 | 1 |
| [77] | Wind Runners: Designing a Game to Encourage Medical Adherence for Children with Asthma | 2012 | Nikkila et al. | 0 | 0 | 1 | 0 | 0 | 0 | 0 | 0 | 0 | 0 |
| [242] | Applying Human-Centered Design to the Development of an Asthma Essentials Kit for School-Aged Children and Their Parents | 2019 | Sonney et al. | 0 | 0 | 0 | 0 | 0 | 0 | 0 | 1 | 0 | 1 |
| [314] | MobileSpiro: Portable Open-Interface Spirometry for Android | 2011 | Gupta et al. | 0 | 0 | 1 | 0 | 0 | 0 | 0 | 0 | 0 | 0 |
| [315] | A PDA-based Network for Telemonitoring Asthma Triggering Gases in the El Paso School Districts of the US - Mexico Border Region | 2005 | Shenoy et al. | 1 | 0 | 0 | 0 | 0 | 0 | 0 | 0 | 0 | 0 |
| [139] | Automatic identification of wheezing in auscultated lung sounds | 2016 | Kang et al. | 0 | 0 | 0 | 0 | 0 | 0 | 0 | 0 | 1 | 0 |
| [29] | Wearable and Stationary Point-of-Care IoT Air Pollution Sensors for Pediatric Asthma Research and Management* | 2019 | Dong et al. | 1 | 0 | 0 | 0 | 0 | 0 | 0 | 0 | 0 | 0 |
| [150] | Design of a smartphone application to monitor stress, asthma symptoms, and asthma inhaler use. | 2015 | Dzubur et al. | 0 | 0 | 0 | 0 | 0 | 0 | 1 | 1 | 0 | 0 |
| [31] | Wireless Sensor-Dependent Ecological Momentary Assessment for Pediatric Asthma mHealth Applications. | 2017 | Buonocore et al. | 1 | 0 | 1 | 1 | 1 | 0 | 1 | 0 | 0 | 1 |
| [47] | Exhalation flow and pressure-controlled reservoir collection of exhaled nitric oxide for remote and delayed analysis. | 1998 | Paredi et al. | 0 | 1 | 0 | 0 | 0 | 0 | 0 | 0 | 0 | 0 |
| [151] | Patient and Parent Perspectives on Improving Pediatric Asthma Self-Management Through a Mobile Health Intervention: Pilot Study. | 2020 | Nichols et al. | 0 | 0 | 0 | 0 | 0 | 0 | 1 | 1 | 0 | 1 |
| [316] | A Novel Data Mining Mechanism Considering Bio-Signal and Environmental Data with Applications on Asthma Monitoring | 2011 | Lee et al. | 1 | 0 | 0 | 0 | 0 | 0 | 0 | 0 | 0 | 0 |
| [278] | Asthma-Nauts: Apps Using Gameplay to Collect Health Metrics and Educate Kids About Asthma | 2019 | Jayaprakash et al. | 0 | 0 | 0 | 0 | 0 | 0 | 0 | 0 | 0 | 1 |
| [284] | Adolescent asthma self-management: patient and parent-caregiver perspectives on using social media to improve care. | 2013 | Panzera et al. | 0 | 0 | 0 | 0 | 0 | 0 | 0 | 0 | 0 | 1 |
| [106] | Peak expiratory flow rate control chart in asthma care: chart construction and use in asthma care. | 1998 | Boggs et al. | 0 | 0 | 1 | 0 | 0 | 0 | 0 | 0 | 0 | 0 |
| [257] | Patient Perspectives on a Text Messaging Program to Support Asthma Management: A Qualitative Study. | 2019 | Doyle et al. | 0 | 0 | 0 | 0 | 0 | 0 | 0 | 0 | 0 | 1 |
| [244] | "Kiss myAsthma": Using a participatory design approach to develop a self-management app with young people with asthma. | 2018 | Davis et al. | 0 | 0 | 0 | 0 | 0 | 0 | 0 | 0 | 0 | 1 |
| [34] | Biomedical REAl-Time Health Evaluation (BREATHE): toward an mHealth informatics platform. | 2020 | Bui et al. | 1 | 0 | 1 | 1 | 1 | 0 | 0 | 1 | 0 | 1 |
| [27] | A Wearable IoT Aldehyde Sensor for Pediatric Asthma Research and Management. | 2019 | Li et al. | 1 | 0 | 0 | 0 | 0 | 0 | 0 | 0 | 0 | 0 |
| [317] | Asthmon: Empowering Asthmatic Children's Self-Management with a Virtual Pet | 2010 | Lee et al. | 0 | 0 | 1 | 0 | 0 | 0 | 0 | 0 | 0 | 0 |
| [245] | Patient and Family Engagement in the Design of a Mobile Health Solution for Pediatric Asthma: Development and Feasibility Study. | 2018 | McWilliams et al. | 0 | 0 | 0 | 0 | 0 | 0 | 0 | 0 | 0 | 1 |
| [282] | Parent and Clinician Preferences for an Asthma App to Promote Adolescent Self-Management: A Formative Study. | 2016 | Geryk et al. | 0 | 0 | 0 | 0 | 0 | 0 | 0 | 0 | 0 | 1 |
| [119] | PhD Forum: Multimodal IoT and EMR based Smart Health Application for Asthma Management in Children. | 2017 | Jaimini et al. | 1 | 1 | 1 | 0 | 1 | 0 | 1 | 0 | 0 | 0 |
| [132] | Identi-wheez - A device for in-home diagnosis of asthma. | 2016 | Satat et al. | 0 | 0 | 0 | 0 | 0 | 0 | 0 | 0 | 1 | 0 |
| [262] | Development of online diary and self-management system on e-Healthcare for asthmatic children in Taiwan. | 2014 | Lin et al. | 0 | 0 | 0 | 0 | 0 | 0 | 0 | 0 | 0 | 1 |
| [189] | Feasibility of a novel mHealth management system to capture and improve medication adherence among adolescents with asthma. | 2016 | Cushing et al. | 0 | 0 | 0 | 0 | 0 | 0 | 0 | 1 | 0 | 1 |
| [176] | Smart electronic dose counter for pressurized metered dose inhaler | 2015 | Chen et al. | 0 | 0 | 0 | 0 | 0 | 0 | 0 | 1 | 0 | 0 |
| [258] | Physicians' perceptions of mobile technology for enhancing asthma care for youth. | 2016 | Schneider et al. | 0 | 0 | 0 | 0 | 0 | 0 | 0 | 0 | 0 | 1 |
| [267] | Building patient relationships: a smartphone application supporting communication between teenagers with asthma and the RN care coordinator. | 2013 | Haze et al. | 0 | 0 | 0 | 0 | 0 | 0 | 0 | 0 | 0 | 1 |
| [318] | Architectural and data model of clinical decision support system for managing asthma in school-aged children | 2011 | Aleksovska-Stojkovska et al. | 0 | 0 | 1 | 0 | 0 | 0 | 0 | 0 | 0 | 0 |
| [283] | InSpire to promote lung assessment in youth: Evolving the self-management paradigms of young people with asthma | 2013 | Elias et al. | 0 | 0 | 1 | 0 | 0 | 0 | 0 | 0 | 0 | 1 |
| [266] | Exploring the theoretical pathways through which asthma app features can promote adolescent self-management. | 2016 | Carpenter et al. | 0 | 0 | 0 | 0 | 0 | 0 | 0 | 0 | 0 | 1 |
| [268] | There is an "app" for that: Designing mobile phone technology to improve asthma action plan use in adolescent patients. | 2016 | Odom et al. | 0 | 0 | 0 | 0 | 0 | 0 | 0 | 0 | 0 | 1 |
| [231] | Effective Engagement of Adolescent Asthma Patients With Mobile Health-Supporting Medication Adherence. | 2019 | Kosse et al. | 0 | 0 | 0 | 0 | 0 | 0 | 0 | 1 | 0 | 1 |
| [164] | Barriers and Facilitators When Implementing Web-Based Disease Monitoring and Management as a Substitution for Regular Outpatient Care in Pediatric Asthma: Qualitative Survey Study. | 2018 | van den Wijngaart et al. | 0 | 0 | 0 | 0 | 0 | 0 | 1 | 0 | 0 | 1 |
| [256] | Adaptive Mobile Health Intervention for Adolescents with Asthma: Iterative User-Centered Development. | 2020 | Fedele et al. | 0 | 0 | 0 | 0 | 0 | 0 | 0 | 1 | 0 | 1 |
| [219] | Feasibility of medication monitoring sensors in high risk asthmatic children. | 2019 | Hoch et al. | 0 | 0 | 0 | 0 | 0 | 0 | 0 | 1 | 0 | 1 |
| [274] | Physician usage of technology and opportunities for continuous care management of pediatric asthma patients | 2010 | Osuntogun et al. | 0 | 0 | 0 | 0 | 0 | 0 | 0 | 0 | 0 | 1 |
| [23] | Design and clinical feasibility of personal wearable monitor for measurement of activity and environmental exposure. | 2014 | Fletcher et al. | 1 | 0 | 0 | 0 | 1 | 0 | 0 | 0 | 1 | 1 |
| [319] | Designing a respiratory CO2 measurement device for home monitoring of asthma severity | 2014 | Zaharudin et al. | 0 | 0 | 0 | 1 | 0 | 0 | 0 | 0 | 0 | 0 |
| [24] | Feasibility of a Secure Wireless Sensing Smartwatch Application for the Self-Management of Pediatric Asthma. | 2017 | Hosseini et al. | 1 | 0 | 1 | 1 | 1 | 0 | 0 | 0 | 0 | 1 |
| [320] | An unidentified monster in the bed--assessing nocturnal asthma in children. | 2009 | Ginsberg et al. | 0 | 0 | 0 | 0 | 0 | 0 | 0 | 0 | 1 | 0 |
| [187] | kBot: Knowledge-enabled Personalized Chatbot for Asthma Self-Management. | 2019 | Kadariya et al. | 0 | 0 | 0 | 0 | 0 | 0 | 0 | 1 | 0 | 1 |
| [263] | Internet telehealth for pediatric asthma case management: integrating computerized and case manager features for tailoring a Web-based asthma education program. | 2007 | Wise et al. | 0 | 0 | 0 | 0 | 0 | 0 | 0 | 0 | 0 | 1 |
| [261] | Aspira: Employing a serious game in an mHealth app to improve asthma outcomes | 2017 | Thomson et al. | 1 | 0 | 1 | 0 | 0 | 0 | 0 | 0 | 0 | 1 |
| [183] | Applying Interactive Mobile health to Asthma Care in Teens (AIM2ACT): Development and design of a randomized controlled trial. | 2018 | Fedele et al. | 0 | 0 | 1 | 0 | 0 | 0 | 0 | 1 | 0 | 1 |
| [264] | Tracking and Reporting Asthma Data for Children | 2019 | Nyapathy et al. | 0 | 0 | 0 | 0 | 0 | 0 | 0 | 0 | 0 | 1 |
| [279] | Beneficial Features of a mHealth Asthma App for Children and Caregivers: Qualitative Study. | 2020 | Iio et al. | 0 | 0 | 0 | 0 | 0 | 0 | 0 | 0 | 0 | 1 |
| [280] | Protocol for the Development of a Behavioral Family Lifestyle Intervention Supported by Mobile Health to Improve Weight Self-Management in Children With Asthma and Obesity. | 2019 | Fedele et al. | 0 | 0 | 0 | 0 | 0 | 0 | 0 | 0 | 0 | 1 |
| [270] | Smartphone app for monitoring asthma in children and adolescents | 2021 | Mayoral et al. | 0 | 0 | 0 | 0 | 0 | 0 | 1 | 0 | 0 | 1 |
| [220] | Training health care providers to conduct adherence promotion in pediatric settings: An example with pediatric asthma | 2013 | Rohan et al. | 0 | 0 | 0 | 0 | 0 | 0 | 0 | 1 | 0 | 0 |
| [25] | The Fresh Air Wristband: A Wearable Air Pollutant Sampler | 2020 | Lin et al. | 1 | 0 | 0 | 0 | 0 | 0 | 0 | 0 | 0 | 0 |
| [236] | AIR Louisville: Addressing asthma with technology, crowdsourcing, cross-sector collaboration, and policy | 2018 | Barrett et al. | 0 | 0 | 0 | 0 | 0 | 0 | 0 | 1 | 0 | 1 |
| [188] | Design and development of a non-volatile counter for metered dose inhaler (MDI) | 2018 | Hin et al. | 0 | 0 | 0 | 0 | 0 | 0 | 0 | 1 | 0 | 0 |
| [255] | Examination of the uses, needs, and preferences for health technology use in adolescents with asthma | 2019 | Ramsey et al. | 0 | 0 | 0 | 0 | 0 | 0 | 0 | 0 | 0 | 1 |
| [281] | Astmapp: A platform for asthma self-management | 2018 | Luna-Aveiga et al. | 0 | 0 | 0 | 0 | 0 | 0 | 0 | 0 | 0 | 1 |
| [186] | How inspiring is your app? A usability take on an app for asthmamedication adherence | 2019 | Ferreira et al. | 0 | 0 | 0 | 0 | 0 | 0 | 0 | 1 | 0 | 1 |
| [130] | Mobile nocturnal long-term monitoring of wheezing and cough | 2007 | Gross et al. | 0 | 0 | 0 | 0 | 0 | 0 | 0 | 0 | 1 | 0 |
| [138] | Rapid wheezing detection algorithm for real-time asthma diagnosis and personal health care | 2008 | Yu et al. | 0 | 0 | 0 | 0 | 0 | 0 | 0 | 0 | 1 | 0 |
| [246] | A Shared e-decision support portal for pediatric asthma | 2014 | Fiks et al. | 0 | 0 | 0 | 0 | 0 | 0 | 0 | 0 | 0 | 1 |
| [321] | A participant-based approach to indoor/outdoor air monitoring in community health studies | 2009 | Johnson et al. | 1 | 0 | 0 | 0 | 0 | 0 | 0 | 0 | 0 | 0 |
| [141] | Automated cough assessment on a mobile platform | 2014 | Sterling et al. | 0 | 0 | 0 | 0 | 0 | 0 | 0 | 0 | 1 | 0 |
| [82] | Experience with home telehealth to support disease management in teenagers with asthma | 2006 | Cai et al. | 0 | 0 | 1 | 0 | 0 | 0 | 0 | 0 | 0 | 0 |
| [272] | Mobile phone-based asthma self-management aid for adolescents (mASMAA): A feasibility study | 2014 | Rhee et al. | 0 | 0 | 0 | 0 | 0 | 0 | 0 | 0 | 0 | 1 |
| [128] | Computer-assisted assay of respiratory sounds of children suffering from bronchial asthma | 2014 | Furman et al. | 0 | 0 | 0 | 0 | 0 | 0 | 0 | 0 | 1 | 0 |
| [271] | Development of a novel tool for engaging children and parents in asthma self-management. | 2012 | Nkoy et al. | 0 | 0 | 0 | 0 | 0 | 0 | 0 | 0 | 0 | 1 |
| [140] | A signal processing approach for the diagnosis of asthma from cough sounds | 2013 | Al-Khassaweneh et al. | 0 | 0 | 0 | 0 | 0 | 0 | 0 | 0 | 1 | 0 |
| [125] | A microcomputer-based interactive cough sound analysis system | 1991 | William Thorpe et al. | 0 | 0 | 0 | 0 | 0 | 0 | 0 | 0 | 1 | 0 |
| [126] | Towards a quantitative description of asthmatic cough sounds | 1992 | Thorpe et al. | 0 | 0 | 0 | 0 | 0 | 0 | 0 | 0 | 1 | 0 |
| [127] | Cough sound analysis: A new tool for the diagnosis of asthma? | 1989 | Toop et al. | 0 | 0 | 0 | 0 | 0 | 0 | 0 | 0 | 1 | 0 |
| [59] | Assessment of a new low-cost portable spirometer (VMPlus, Clement Clarke) for use in children  Dezelfde als 289, alleen ander jaartal, maar 2002 klopt (?) | 1999 | Bastian-Lee et al. | 0 | 0 | 1 | 0 | 0 | 0 | 0 | 0 | 0 | 0 |
| [276] | Internet Telehealth for Pediatric Asthma Case Management | 2005 | NCT00214383 | 0 | 0 | 0 | 0 | 0 | 0 | 0 | 0 | 0 | 1 |
| [247] | Using a co-design approach to develop an appealing goal-setting and self-management app for young people with asthma | 2017 | Foster et al. | 0 | 0 | 0 | 0 | 0 | 0 | 0 | 0 | 0 | 1 |
| [172] | Assessing adolescent asthma symptoms and adherence using mobile phones. | 2013 | Mulvaney et al. | 0 | 0 | 0 | 0 | 0 | 0 | 0 | 1 | 0 | 0 |
| [253] | Adolescent Preferences and Design Recommendations for an Asthma Self-Management App: Mixed-Methods Study. | 2018 | Roberts et al. | 0 | 0 | 0 | 0 | 0 | 0 | 0 | 0 | 0 | 1 |
| [254] | Adolescent, Caregiver, and Provider Preferences for an Asthma Self-Management App | 2015 | Carpenter et al. | 0 | 0 | 0 | 0 | 0 | 0 | 0 | 0 | 0 | 1 |
| ***PHASE: VALIDATION*** | |  |  |  |  |  |  |  |  |  |  |  |  |
| [190] | Methods of assessing adherence to inhaled corticosteroid therapy in children and adolescents: adherence rates and their implications for clinical practice. | 2008 | Jentzsch et al. | 0 | 0 | 0 | 0 | 0 | 0 | 0 | 1 | 0 | 0 |
| [203] | A tailored mobile health intervention to improve adherence and asthma control in minority adolescents | 2015 | Mosnaim et al. | 0 | 0 | 0 | 0 | 0 | 0 | 0 | 1 | 0 | 1 |
| [322] | Predicting asthma control deterioration in children. | 2015 | Luo et al. | 0 | 0 | 0 | 0 | 0 | 0 | 1 | 0 | 0 | 0 |
| [229] | Mobile direct observation of therapy (MDOT) - A rapid systematic review and pilot study in children with asthma. | 2018 | Shields et al. | 0 | 0 | 0 | 0 | 0 | 0 | 0 | 1 | 1 | 1 |
| [105] | Effect of the whistle watch device on bronchodilator use in children with asthma. | 2001 | Savva et al. | 0 | 0 | 1 | 0 | 0 | 0 | 0 | 0 | 0 | 0 |
| [323] | Lung Function Variability in Children and Adolescents With and Without Asthma (LUV Study): Protocol for a Prospective, Nonrandomized, Clinical Trial. | 2020 | Frima et al. | 0 | 0 | 1 | 0 | 0 | 0 | 0 | 0 | 0 | 0 |
| [324] | Expiratory variability index (EVI) is associated with the severity of acute bronchial obstruction in small children: A proof-of-concept study. | 2020 | Seppä et al. | 0 | 0 | 1 | 0 | 0 | 0 | 0 | 0 | 0 | 0 |
| [74] | Evaluation of daily home spirometry for school children with asthma: new insights. | 2006 | Thompson et al. | 0 | 0 | 1 | 0 | 0 | 0 | 0 | 0 | 0 | 0 |
| [191] | Adherence to medication in children and adolescents with asthma: methods for monitoring and intervention. | 2018 | Pearce et al. | 0 | 0 | 0 | 0 | 0 | 0 | 0 | 1 | 0 | 0 |
| [22] | Night cough in a population-based sample of children: characteristics, relation to symptoms and associations with measures of asthma severity. | 1996 | Brooke et al. | 1 | 0 | 1 | 0 | 0 | 0 | 0 | 0 | 1 | 0 |
| [134] | Cough frequency in children with stable asthma: correlation with lung function, exhaled nitric oxide, and sputum eosinophil count. | 2003 | Li et al. | 0 | 0 | 0 | 0 | 0 | 0 | 0 | 0 | 1 | 0 |
| [36] | Panel studies of air pollution on children's lung function and respiratory symptoms: a literature review. | 2012 | Li et al. | 1 | 0 | 1 | 0 | 0 | 0 | 0 | 0 | 0 | 0 |
| [174] | Use of metered-dose inhaler electronic monitoring in a real-world asthma randomized controlled trial. | 2013 | Patel et al. | 0 | 0 | 0 | 0 | 0 | 0 | 0 | 1 | 0 | 0 |
| [259] | Engagement in "My Child's Asthma", an interactive web-based pediatric asthma management intervention. | 2011 | Meischke et al. | 0 | 0 | 0 | 0 | 0 | 0 | 0 | 0 | 0 | 1 |
| [73] | Evaluating the use of a portable spirometer in a study of pediatric asthma. | 2003 | Mortimer et al. | 0 | 0 | 1 | 0 | 0 | 0 | 0 | 0 | 0 | 0 |
| [325] | Adolescent and caregivers' experiences of electronic adherence assessment in paediatric problematic severe asthma. | 2018 | Stewart et al. | 0 | 0 | 0 | 0 | 0 | 0 | 0 | 1 | 0 | 0 |
| [146] | Seasonal variation in 1/f fluctuations of heart rate in asthmatic children. | 2001 | Kazuma et al. | 0 | 0 | 0 | 1 | 0 | 0 | 0 | 0 | 0 | 0 |
| [159] | Longitudinal validation of a tool for asthma self-monitoring. | 2013 | Nkoy et al. | 0 | 0 | 0 | 0 | 0 | 0 | 1 | 0 | 0 | 0 |
| [168] | Medication adherence in the asthmatic child and adolescent. | 2011 | Desai et al. | 0 | 0 | 0 | 0 | 0 | 0 | 0 | 1 | 0 | 0 |
| [70] | Is home monitoring of lung function worthwhile for children with asthma? | 2001 | Sly et al. | 0 | 0 | 1 | 0 | 0 | 0 | 0 | 0 | 0 | 0 |
| [120] | Exploring the Association Between Self-Reported Asthma Impact and Fitbit-Derived Sleep Quality and Physical Activity Measures in Adolescents. | 2017 | Bian et al. | 0 | 0 | 0 | 0 | 1 | 1 | 0 | 0 | 0 | 0 |
| [326] | A Cloud-Connected NO2 and Ozone Sensor System for Personalized Pediatric Asthma Research and Management | 2020 | Dong et al. | 1 | 0 | 0 | 0 | 0 | 0 | 0 | 0 | 0 | 0 |
| [327] | Using Video from Mobile Phones to Improve Pediatric Phone Triage in an Underserved Population. | 2017 | Freeman et al. | 0 | 0 | 0 | 0 | 0 | 0 | 0 | 0 | 1 | 0 |
| [328] | Development of temporally refined land-use regression models predicting daily household-level air pollution in a panel study of lung function among asthmatic children. | 2013 | Johnson et al. | 1 | 0 | 0 | 0 | 0 | 0 | 0 | 0 | 0 | 0 |
| [329] | Assessing physical activity in children with asthma: convergent validity between accelerometer and electronic diary data. | 2009 | Floro et al. | 0 | 0 | 0 | 0 | 1 | 0 | 0 | 0 | 0 | 0 |
| [145] | Seasonal variation in heart rate variability in asthmatic children. | 2000 | Kazuma et al. | 0 | 0 | 0 | 1 | 0 | 0 | 0 | 0 | 0 | 0 |
| [81] | Spirometric patterns in childhood asthma: peak flow compared with other indices. | 1995 | Klein et al. | 0 | 0 | 1 | 0 | 0 | 0 | 0 | 0 | 0 | 0 |
| [182] | Family asthma management routines: connections to medical adherence and quality of life. | 2005 | Fiese et al. | 0 | 0 | 0 | 0 | 0 | 0 | 0 | 1 | 0 | 0 |
| [260] | Assessing asthma management practices through in-home technology probes | 2010 | Yun et al. | 1 | 0 | 1 | 0 | 0 | 0 | 0 | 0 | 0 | 1 |
| [21] | HIPAA Compliant Wireless Sensing Smartwatch Application for the Self-Management of Pediatric Asthma. | 2016 | Hosseini et al. | 1 | 0 | 1 | 1 | 0 | 0 | 0 | 0 | 0 | 1 |
| [250] | Patient-clinician mobile communication: analyzing text messaging between adolescents with asthma and nurse case managers. | 2015 | Yoo et al. | 0 | 0 | 0 | 0 | 0 | 0 | 0 | 0 | 0 | 1 |
| [63] | Peak flow diaries in childhood asthma are unreliable. | 2001 | Kamps et al. | 0 | 0 | 1 | 0 | 0 | 0 | 0 | 0 | 0 | 0 |
| [54] | Interactive effects of family functioning and sleep experiences on daily lung functioning in pediatric asthma: An ecological momentary assessment approach | 2020 | Ghriwati et al. | 0 | 0 | 1 | 0 | 0 | 1 | 1 | 0 | 0 | 1 |
| [39] | In-home particle concentrations and childhood asthma morbidity | 2009 | McCormack et al. | 1 | 0 | 0 | 0 | 0 | 0 | 0 | 0 | 0 | 0 |
| [62] | Discrepancy between Lung Function Measurements at Home and in the Hospital in Children with Asthma and CF | 2020 | Gerzon et al. | 0 | 0 | 1 | 0 | 0 | 0 | 0 | 0 | 0 | 0 |
| [158] | Electronic monitoring of symptoms and lung function to assess asthma control in children. | 2014 | van Vliet et al. | 0 | 0 | 1 | 0 | 0 | 0 | 1 | 0 | 0 | 0 |
| [330] | Perceptions of asthma by adolescents at home. | 2000 | Rietveld et al. | 0 | 0 | 0 | 0 | 0 | 0 | 0 | 0 | 1 | 0 |
| [45] | An Australian national panel study of diurnal temperature range and children's respiratory health. | 2014 | Li et al. | 1 | 0 | 1 | 0 | 0 | 0 | 0 | 0 | 0 | 0 |
| [175] | Measuring adherence with the Doser CT in children with asthma. | 2004 | O'Connor et al. | 0 | 0 | 0 | 0 | 0 | 0 | 0 | 1 | 0 | 0 |
| [83] | Pulmonary function in a hospital population of asthmatic children. | 1991 | Foo et al. | 0 | 0 | 1 | 0 | 0 | 0 | 0 | 0 | 0 | 0 |
| [33] | Determination of Personalized Asthma Triggers From Multimodal Sensing and a Mobile App: Observational Study. | 2019 | Venkataramanan et al. | 1 | 0 | 1 | 0 | 1 | 1 | 1 | 1 | 0 | 1 |
| [41] | Effect of air pollution on lung function in schoolchildren in Rio de Janeiro, Brazil. | 2009 | Castro et al. | 0 | 0 | 1 | 0 | 0 | 0 | 0 | 0 | 0 | 0 |
| [28] | "How Is My Child's Asthma?" Digital Phenotype and Actionable Insights for Pediatric Asthma. | 2018 | Jaimini et al. | 1 | 0 | 1 | 0 | 1 | 1 | 1 | 1 | 0 | 1 |
| [50] | Daily ambulatory exhaled nitric oxide measurements in asthma. | 2006 | Pijnenburg et al. | 0 | 1 | 0 | 0 | 0 | 0 | 1 | 0 | 0 | 0 |
| [142] | Evaluating the Validity of an Automated Device for Asthma Monitoring for Adolescents: Correlational Design. | 2015 | Rhee et al. | 0 | 0 | 0 | 0 | 0 | 0 | 1 | 0 | 1 | 0 |
| [331] | Reference values for peak flow and FEV1 variation in healthy schoolchildren using home spirometry | 2008 | Brouwer et al. | 0 | 0 | 1 | 0 | 0 | 0 | 0 | 0 | 0 | 0 |
| [38] | Asthma Exacerbations and Symptom Variability in Children Due to Short-term Ambient Air Pollution Changes in Ostrava, Czech Republic. | 2015 | Velická et al. | 1 | 0 | 0 | 0 | 0 | 0 | 1 | 0 | 0 | 0 |
| [64] | Usefulness of monitoring lung function in asthma. | 2003 | Brand et al. | 0 | 0 | 1 | 0 | 0 | 0 | 0 | 0 | 0 | 0 |
| [60] | Comparison between peak expiratory flow and FEV(1) measurements on a home spirometer and on a pneumotachograph in children with asthma. | 2007 | Brouwer et al. | 0 | 0 | 1 | 0 | 0 | 0 | 0 | 0 | 0 | 0 |
| [135] | Wheezing recognition algorithm using recordings of respiratory sounds at the mouth in a pediatric population. | 2016 | Bokov et al. | 0 | 0 | 0 | 0 | 0 | 0 | 0 | 0 | 1 | 0 |
| [55] | Diagnostics of spontaneous cough in childhood asthma | 1998 | Rietveld et al. | 0 | 0 | 1 | 0 | 0 | 0 | 0 | 0 | 1 | 0 |
| [65] | Accuracy of children's self-reported adherence to treatment. | 2001 | Burkhart et al. | 0 | 0 | 1 | 0 | 0 | 0 | 0 | 0 | 0 | 0 |
| [44] | Effects of personal particulate matter on peak expiratory flow rate of asthmatic children. | 2007 | Tang et al. | 1 | 0 | 1 | 0 | 0 | 0 | 1 | 0 | 0 | 0 |
| [53] | Daily exhaled nitric oxide measurements and asthma exacerbations in children. | 2012 | van der Valk et al. | 0 | 1 | 0 | 0 | 0 | 0 | 1 | 0 | 0 | 0 |
| [51] | Daily home measurements of exhaled nitric oxide in asthmatic children during natural birch pollen exposure. | 2006 | Vahlkvist et al. | 0 | 1 | 1 | 0 | 0 | 0 | 0 | 0 | 0 | 0 |
| [56] | Characteristics and diagnostic significance of spontaneous wheezing in children with asthma: results of continuous in vivo sound recording. | 1999 | Rietveld et al. | 0 | 0 | 1 | 0 | 0 | 0 | 0 | 0 | 1 | 0 |
| [332] | Traffic-related exposures, airway function, inflammation, and respiratory symptoms in children. | 2007 | Holguin et al. | 1 | 0 | 0 | 0 | 0 | 0 | 0 | 0 | 0 | 0 |
| [333] | Contribution of indoor and outdoor environments to PM2.5 personal exposure of children--VESTA study. | 2002 | Gauvin et al. | 1 | 0 | 0 | 0 | 0 | 0 | 0 | 0 | 0 | 0 |
| [79] | Home recording of PEF in young asthmatics: does it contribute to management? | 1996 | Uwyyed et al. | 0 | 0 | 1 | 0 | 0 | 0 | 0 | 0 | 0 | 0 |
| [35] | Knowledge-Driven Personalized Contextual mHealth Service for Asthma Management in Children | 2015 | Anantharam et al. | 1 | 1 | 1 | 0 | 0 | 0 | 1 | 0 | 1 | 1 |
| [84] | Home spirometry and asthma severity in children. | 2006 | Brouwer et al. | 0 | 0 | 1 | 0 | 0 | 0 | 0 | 0 | 0 | 0 |
| [334] | Asthma in life context: Video Intervention/Prevention Assessment (VIA). | 2000 | Rich et al. | 0 | 0 | 0 | 0 | 0 | 0 | 0 | 0 | 1 | 0 |
| [156] | Using information technology to reduce asthma disparities in underserved populations: a pilot study. | 2010 | Vargas et al. | 0 | 0 | 0 | 0 | 0 | 0 | 1 | 0 | 0 | 0 |
| [72] | The quality of home spirometry in school children with asthma. | 2001 | Wensley et al. | 0 | 0 | 1 | 0 | 0 | 0 | 0 | 0 | 0 | 0 |
| [122] | Featured Article: Multiple Comorbid Conditions, Sleep Quality and Duration, and Academic Performance in Urban Children With Asthma. | 2018 | Reynolds et al. | 0 | 0 | 0 | 0 | 0 | 1 | 0 | 0 | 0 | 0 |
| [37] | A longitudinal study of indoor nitrogen dioxide levels and respiratory symptoms in inner-city children with asthma. | 2008 | Hansel et al. | 1 | 0 | 0 | 0 | 0 | 0 | 0 | 0 | 0 | 0 |
| [192] | Monitoring adherence to beclomethasone in asthmatic children and adolescents through four different methods. | 2009 | Jentzsch et al. | 0 | 0 | 0 | 0 | 0 | 0 | 0 | 1 | 0 | 0 |
| [335] | Comparison of normal and asthmatic circadian rhythms in peak expiratory flow rate. | 1980 | Hetzel et al. | 0 | 0 | 1 | 0 | 0 | 0 | 0 | 0 | 0 | 0 |
| [155] | Electronic versus paper questionnaires: a further comparison in persons with asthma. | 2003 | Bushnell et al. | 0 | 0 | 0 | 0 | 0 | 0 | 1 | 0 | 0 | 0 |
| [66] | Reduction in resource utilization by an asthma outreach program. | 1995 | Greineder et al. | 0 | 0 | 1 | 0 | 0 | 0 | 0 | 0 | 0 | 0 |
| [336] | Personal exposures to traffic-related particle pollution among children with asthma in the South Bronx, NY. | 2010 | Spira-Cohen et al. | 1 | 0 | 0 | 0 | 0 | 0 | 0 | 0 | 0 | 0 |
| [71] | Reproducibility of home spirometry in children with newly diagnosed asthma. | 2000 | Pelkonen et al. | 0 | 0 | 1 | 0 | 0 | 0 | 0 | 0 | 0 | 0 |
| [144] | Heart rate variability during 24 hours in asthmatic children. | 1997 | Kazuma et al. | 0 | 0 | 0 | 1 | 0 | 0 | 0 | 0 | 0 | 0 |
| [133] | Cough frequency in children with mild asthma correlates with sputum neutrophil count. | 2006 | Li et al. | 0 | 0 | 0 | 0 | 0 | 0 | 0 | 0 | 1 | 0 |
| [131] | Soft stethoscope for detecting asthma wheeze in young children. | 2013 | Yu et al. | 0 | 0 | 0 | 0 | 0 | 0 | 0 | 0 | 1 | 0 |
| [143] | Wheeze monitoring in children for assessment of nocturnal asthma and response to therapy. | 2003 | Bentur et al. | 0 | 0 | 0 | 0 | 0 | 0 | 0 | 0 | 1 | 0 |
| [80] | Twice-daily peak expiratory flow rate monitoring for the assessment of childhood asthma. | 1993 | Linna et al. | 0 | 0 | 1 | 0 | 0 | 0 | 0 | 0 | 0 | 0 |
| [59] | Assessment of a low-cost home monitoring spirometer for children. | 2002 | Bastian-Lee, et al. | 0 | 0 | 1 | 0 | 0 | 0 | 0 | 0 | 0 | 0 |
| [67] | The validity and acceptability of a text-based monitoring system for pediatric asthma studies. | 2016 | Gahleitner et al. | 0 | 0 | 1 | 0 | 0 | 0 | 0 | 0 | 0 | 1 |
| [177] | Patterns of quick-relief and long-term controller medication use in pediatric asthma. | 2005 | Walders et al. | 0 | 0 | 0 | 0 | 0 | 0 | 0 | 1 | 0 | 0 |
| [85] | Airway obstruction at time of symptoms prompting use of reliever therapy in children with asthma. | 2010 | Brouwer et al. | 0 | 0 | 1 | 0 | 0 | 0 | 0 | 0 | 0 | 0 |
| [337] | A cluster-randomized trial to provide clinicians inhaled corticosteroid adherence information for their patients with asthma. | 2010 | Williams et al. | 0 | 0 | 0 | 0 | 0 | 0 | 0 | 1 | 0 | 0 |
| [57] | Sleep and pulmonary function in children with well-controlled, stable asthma. | 1998 | Sadeh et al. | 0 | 0 | 1 | 0 | 0 | 1 | 0 | 0 | 0 | 0 |
| [338] | Particle concentrations in inner-city homes of children with asthma: the effect of smoking, cooking, and outdoor pollution. | 2003 | Wallace et al. | 1 | 0 | 0 | 0 | 0 | 0 | 0 | 0 | 0 | 0 |
| [86] | Accuracy of mini peak flow meters in indicating changes in lung function in children with asthma. | 1994 | Sly et al. | 0 | 0 | 1 | 0 | 0 | 0 | 0 | 0 | 0 | 0 |
| [121] | Decreased physical activity among Head Start children with a history of wheezing: use of an accelerometer to measure activity. | 2005 | Firrincieli et al. | 0 | 0 | 0 | 0 | 1 | 0 | 0 | 0 | 0 | 0 |
| [40] | Relationship of endotoxin and tobacco smoke exposure to wheeze and diurnal peak expiratory flow variability in children and adolescents. | 2011 | Lawson et al. | 1 | 0 | 1 | 0 | 0 | 0 | 0 | 0 | 0 | 0 |
| [181] | Symptom perception and functional morbidity across a 1-year follow-up in pediatric asthma. | 2007 | Feldman et al. | 0 | 0 | 1 | 0 | 0 | 0 | 0 | 0 | 0 | 0 |
| [339] | Ambulatory monitoring of peak expiratory flow. Reproducibility and quality control. | 1995 | Enright et al. | 0 | 0 | 1 | 0 | 0 | 0 | 0 | 0 | 0 | 0 |
| [269] | PELICAN: Content evaluation of patient-centered care for children with asthma based on an online tool. | 2016 | van Bragt et al. | 0 | 0 | 0 | 0 | 0 | 0 | 1 | 0 | 0 | 1 |
| [43] | The role of traffic-related air pollution in pm-health effects associations among inner city children with asthma | 2010 | Spira-Cohen et al. | 1 | 0 | 1 | 0 | 0 | 0 | 0 | 0 | 0 | 0 |
| [340] | Improving adherence to inhaled corticosteroids in children with asthma | 2003 | Kamps et al. | 0 | 0 | 0 | 0 | 0 | 0 | 0 | 1 | 0 | 0 |
| [341] | Perceptions of Asthma and Exercise, and Associations With Weight Status and Asthma Morbidity in Urban Children | 2020 | Eisenberg et al. | 0 | 0 | 1 | 0 | 0 | 0 | 0 | 0 | 0 | 0 |
| [342] | Home-based forced Oscillation technique day-to-day variability in Pediatric Asthma | 2019 | Wong et al. | 0 | 0 | 1 | 0 | 0 | 0 | 0 | 0 | 0 | 0 |
| [147] | Bringing technology to day-to-day asthma management | 2018 | Messinger et al. | 0 | 0 | 0 | 1 | 0 | 1 | 0 | 0 | 0 | 0 |
| [265] | Measurement properties of an asthma symptom and rescue medication use diary | 2015 | Clark et al. | 0 | 0 | 0 | 0 | 0 | 0 | 0 | 0 | 0 | 1 |
| [248] | Asthma app use and interest among patients with asthma: A multicenter study | 2020 | Jácome et al. | 0 | 0 | 0 | 0 | 0 | 0 | 0 | 0 | 0 | 1 |
| [343] | Electronic monitoring of adherence to inhaled corticosteroids: An essential tool in identifying severe asthma in children | 2018 | Nimmagadda et al. | 0 | 0 | 0 | 0 | 0 | 0 | 0 | 1 | 0 | 0 |
| [26] | A field application of a personal sensor for ultrafine particle exposure in children | 2015 | Ryan et al. | 1 | 0 | 0 | 0 | 0 | 0 | 0 | 0 | 0 | 0 |
| [178] | Monitoring nebulizer use in children: Comparison of electronic and asthma diary data | 2005 | Butz et al. | 0 | 0 | 0 | 0 | 0 | 0 | 0 | 1 | 0 | 0 |
| [184] | True adherence with the Turbuhaler in young children with asthma | 2011 | Nikander et al. | 0 | 0 | 0 | 0 | 0 | 0 | 0 | 1 | 0 | 0 |
| [157] | Computerised paediatric asthma quality of life questionnaires in routine care | 2007 | Mussaffi et al. | 0 | 0 | 0 | 0 | 0 | 0 | 1 | 0 | 0 | 0 |
| [344] | Exposure assessment and modeling of particulate matter for asthmatic children using personal nephelometers | 2005 | Wu et al. | 1 | 0 | 0 | 0 | 0 | 0 | 0 | 0 | 0 | 0 |
| [49] | Exhaled nitric oxide daily evaluation is effective in monitoring exposure to relevant allergens in asthmatic children | 2007 | Bodini et al. | 0 | 1 | 1 | 0 | 0 | 0 | 0 | 0 | 0 | 0 |
| [171] | Utilizing computerized phone diary procedures to assess health behaviors in family and social contexts | 2006 | Modi et al. | 0 | 0 | 0 | 0 | 0 | 0 | 0 | 1 | 0 | 0 |
| [42] | Personal and ambient air pollution exposures and lung function decrements in children with asthma | 2008 | Delfino et al. | 1 | 0 | 1 | 0 | 0 | 0 | 0 | 1 | 0 | 0 |
| [30] | Development and in-home testing of the pretoddler inhalable particulate environmental robotic (PIPER Mk IV) sampler | 2011 | Shalat et al. | 1 | 0 | 0 | 0 | 0 | 0 | 0 | 0 | 0 | 0 |
| [345] | Household levels of nitrogen dioxide and pediatric asthma severity | 2013 | Belanger et al. | 1 | 0 | 0 | 0 | 0 | 0 | 0 | 0 | 0 | 0 |
| [346] | Intranasal air sampling in homes: Relationships among reservoir allergen concentrations and asthma severity | 2006 | Gore et al. | 1 | 0 | 0 | 0 | 0 | 0 | 0 | 0 | 0 | 0 |
| [153] | An electronic diary is shown to be more reliable than a paper diary: Results from a randomized crossover study in patients with persistent asthma | 2012 | Ireland et al. | 0 | 0 | 0 | 0 | 0 | 0 | 1 | 0 | 0 | 1 |
| [48] | Comparability of a hand-held nitric oxide analyser with online and offline chemiluminescence-based nitric oxide measurement | 2009 | Schiller et al. | 0 | 1 | 0 | 0 | 0 | 0 | 0 | 0 | 0 | 0 |
| [347] | Adherence with a hand-held electronic device versus conventional peak expiratory flow rate monitoring in children with asthma | 2006 | Stahlman et al. | 0 | 0 | 1 | 0 | 0 | 0 | 0 | 0 | 0 | 0 |
| [154] | Internet and written respiratory questionnaires yield equivalent results for adolescents | 2007 | Raat et al. | 0 | 0 | 0 | 0 | 0 | 0 | 1 | 0 | 0 | 0 |
| [169] | Electronic measurement of non-compliance to inhaled corticosteroids in a multicultural population of children with asthma in Amsterdam (Compliance Objectively measured in a Multicultural Population of children Living in Amsterdam Needing inhaled Corticosteroids for Effective asthma treatment; COMPLIANCE) | 2010 | Vasbinder et al. | 0 | 0 | 0 | 0 | 0 | 0 | 0 | 1 | 0 | 0 |
| [179] | Adherence with preventive medication in childhood asthma | 2011 | Burgess et al. | 0 | 0 | 0 | 0 | 0 | 0 | 0 | 1 | 0 | 0 |
| [348] | Validity, reliability and discriminative capacity of an electronic quality of life instrument (Pelican) for childhood asthma in the Netherlands | 2014 | Van Bragt et al. | 0 | 0 | 0 | 0 | 0 | 0 | 1 | 0 | 0 | 0 |
| [69] | Compliance and reliability of electronic PEF monitoring in adolescents with asthma [1] | 2006 | Van Der Meer et al. | 0 | 0 | 1 | 0 | 0 | 0 | 0 | 0 | 0 | 1 |
| [118] | The development of an automated device for asthma monitoring for adolescents: methodologic approach and user acceptability. | 2014 | Rhee et al. | 0 | 0 | 0 | 0 | 1 | 0 | 0 | 1 | 1 | 1 |
| [61] | Inaccuracy of portable peak flow meters: Correction is not needed | 1997 | Brand et al. | 0 | 0 | 1 | 0 | 0 | 0 | 0 | 0 | 0 | 0 |
| [136] | Night cough counts and diary card scores in asthma | 1985 | Archer et al. | 0 | 0 | 0 | 0 | 0 | 0 | 0 | 0 | 1 | 0 |
| [349] | Can peak expiratory flow measurements estimate small airway function in asthmatic children? | 2001 | Goldberg et al. | 0 | 0 | 1 | 0 | 0 | 0 | 0 | 0 | 0 | 0 |
| [350] | Measurement of offline exhaled nitric oxide in a study of community exposure to air pollution | 2003 | Koenig et al. | 1 | 1 | 0 | 0 | 0 | 0 | 0 | 0 | 0 | 0 |
| [351] | A procedure for using peak expiratory flow rate data to increase the predictability of asthma episodes | 1978 | Taplin et al. | 0 | 0 | 1 | 0 | 0 | 0 | 0 | 0 | 0 | 0 |
| [58] | Nocturnal wheeze measurement in young asthmatics | 2004 | Bentur et al. | 0 | 0 | 1 | 0 | 0 | 0 | 0 | 0 | 1 | 0 |
| [352] | Bronchial lability index in the diagnosis of asthma in children | 1999 | Kannisto et al. | 0 | 0 | 1 | 0 | 0 | 0 | 0 | 0 | 0 | 0 |
| [46] | Condensed expirate nitrite as a home marker for acute asthma | 1995 | Hunt et al. | 0 | 1 | 0 | 0 | 0 | 0 | 0 | 0 | 0 | 0 |
| [68] | Reliability of PEF diaries | 2001 | Anees et al. | 0 | 0 | 1 | 0 | 0 | 0 | 0 | 0 | 0 | 0 |
| [180] | Electronic adherence monitoring device performance and patient acceptability: a randomized control trial | 2017 | Chan et al. | 0 | 0 | 0 | 0 | 0 | 0 | 0 | 1 | 0 | 0 |
| [353] | Analysis of the relationship between handheld and clinic-based spirometry measurements in a randomized, double-blind, placebo-controlled study of beclomethasone dipropionate via breath-actuated inhaler for persistent asthma | 2017 | Kerwin et al. | 0 | 0 | 1 | 0 | 0 | 0 | 0 | 0 | 0 | 0 |
| [185] | Impact of interview mode on accuracy of child and parent report of adherence with asthma-controller medication. | 2007 | Bender et al. | 0 | 0 | 0 | 0 | 0 | 0 | 1 | 1 | 0 | 0 |
| [124] | Assessing effects of personal behaviors and environmental exposure on asthma episodes: a diary-based approach. | 2019 | Chan et al. | 1 | 0 | 0 | 0 | 0 | 1 | 0 | 0 | 0 | 1 |
| [354] | Increased levels of outdoor air pollutants are associated with reduced bronchodilation in children with asthma. | 2009 | Hernández-Cadena et al. | 1 | 0 | 0 | 0 | 0 | 0 | 0 | 0 | 0 | 0 |
| [123] | Passive nocturnal physiologic monitoring enables early detection of exacerbations in children with asthma a proof-of-concept study | 2018 | Huffaker et al. | 0 | 0 | 0 | 1 | 0 | 1 | 0 | 0 | 0 | 0 |
| [3] | WEARCON: Wearable home monitoring in children with asthma reveals a strong association with hospital based assessment of asthma control. | 2020 | van der Kamp et al. | 0 | 0 | 1 | 1 | 1 | 1 | 0 | 1 | 0 | 0 |
| ***PHASE: INTERVENTION*** | |  |  |  |  |  |  |  |  |  |  |  |  |
| [173] | Low-cost electronic dose counter for pressurized metered dose inhaler | 2015 | Chen et al. | 0 | 0 | 0 | 0 | 0 | 0 | 0 | 1 | 0 | 0 |
| [212] | e-Monitoring of Asthma Therapy to Improve Compliance in children using a real-time medication monitoring system (RTMM): the e-MATIC study protocol. | 2013 | Vasbinder et al. | 0 | 0 | 0 | 0 | 0 | 0 | 0 | 1 | 0 | 0 |
| [240] | The effect of telepharmacy counseling on metered-dose inhaler technique among adolescents with asthma in rural Arkansas. | 2001 | Bynum et al. | 0 | 0 | 0 | 0 | 0 | 0 | 0 | 1 | 1 | 0 |
| [226] | Telehealth delivery of adherence and medication management system improves outcomes in inner-city children with asthma. | 2020 | Lin et al. | 0 | 0 | 0 | 0 | 0 | 0 | 0 | 1 | 0 | 0 |
| [355] | Monitoring asthma in childhood: symptoms, exacerbations and quality of life. | 2015 | Brand et al. | 0 | 0 | 0 | 0 | 0 | 0 | 1 | 0 | 0 | 0 |
| [287] | Electronic health (e-Health): emerging role in asthma. | 2017 | Bonini et al. | 0 | 0 | 0 | 0 | 0 | 0 | 0 | 0 | 0 | 1 |
| [166] | Randomized trial of an electronic asthma monitoring system among New York City children. | 2009 | Jacobson et al. | 0 | 0 | 0 | 0 | 0 | 0 | 1 | 0 | 0 | 0 |
| [161] | Cost-effectiveness of FENO-based and web-based monitoring in paediatric asthma management: a randomised controlled trial. | 2016 | Beerthuizen et al. | 0 | 0 | 0 | 0 | 0 | 0 | 1 | 0 | 0 | 0 |
| [227] | The impact of peer support and mp3 messaging on adherence to inhaled corticosteroids in minority adolescents with asthma: a randomized, controlled trial. | 2013 | Mosnaim et al. | 0 | 0 | 0 | 0 | 0 | 0 | 0 | 1 | 1 | 0 |
| [209] | mHealth intervention to support asthma self-management in adolescents: the ADAPT study. | 2017 | Kosse et al. | 0 | 0 | 0 | 0 | 0 | 0 | 1 | 1 | 0 | 1 |
| [7] | Smartphone Applications for Encouraging Asthma Self-Management in Adolescents: A Systematic Review. | 2018 | Alquran et al. | 0 | 0 | 0 | 0 | 0 | 0 | 0 | 0 | 0 | 1 |
| [309] | Real-world evaluation of a mobile health application in children with asthma. | 2018 | Stukus et al. | 0 | 0 | 0 | 0 | 0 | 0 | 0 | 0 | 0 | 1 |
| [196] | Does immediate smart feedback on therapy adherence and inhalation technique improve asthma control in children with uncontrolled asthma? A study protocol of the IMAGINE I study. | 2020 | Sportel et al. | 0 | 0 | 1 | 0 | 0 | 0 | 0 | 1 | 0 | 0 |
| [303] | The effectiveness of telegram-based virtual education versus in-person education on the quality of life in adolescents with moderate-to-severe asthma: A pilot randomized controlled trial. | 2020 | Faraji et al. | 0 | 0 | 0 | 0 | 0 | 0 | 0 | 0 | 0 | 1 |
| [304] | Effects of maintaining web-based diaries by caregivers on adherence to care regimens in preschoolers with asthma. | 2019 | Hashi et al. | 0 | 0 | 0 | 0 | 0 | 0 | 0 | 0 | 0 | 1 |
| [8] | Systematic Review of Digital Interventions for Pediatric Asthma Management | 2020 | Ramsey et al. | 0 | 0 | 0 | 0 | 0 | 0 | 0 | 0 | 0 | 1 |
| [213] | Responsive Asthma Care for Teens (ReACT): development protocol for an adaptive mobile health intervention for adolescents with asthma. | 2019 | Cushing et al. | 0 | 0 | 0 | 0 | 0 | 0 | 0 | 1 | 0 | 1 |
| [160] | Outcomes of a population-based asthma management program: quality of life, absenteeism, and utilization. | 2000 | Legorreta et al. | 0 | 0 | 1 | 0 | 0 | 0 | 1 | 0 | 0 | 0 |
| [98] | An internet-based interactive telemonitoring system for improving childhood asthma outcomes in Taiwan. | 2007 | Jan et al. | 0 | 0 | 1 | 0 | 0 | 0 | 1 | 0 | 0 | 1 |
| [109] | Value of home peak flow monitoring for asthma control. | 1995 | Janson, S | 0 | 0 | 1 | 0 | 0 | 0 | 0 | 0 | 0 | 0 |
| [111] | Effects of Symptom Perception Interventions on Trigger Identification and Quality of Life in Children with Asthma | 2015 | Janssens et al. | 0 | 0 | 1 | 0 | 0 | 0 | 0 | 0 | 0 | 0 |
| [293] | The effectiveness of nurse-led telemonitoring of asthma: results of a randomized controlled trial. | 2008 | Willems et al. | 0 | 0 | 0 | 0 | 0 | 0 | 0 | 0 | 0 | 1 |
| [14] | Online asthma management for children is cost-effective. | 2017 | van den Wijngaart et al. | 0 | 0 | 0 | 0 | 0 | 0 | 1 | 0 | 0 | 1 |
| [296] | Phase II trial of web-based tailored asthma management intervention in adolescents at clinics. | 2019 | Lu et al. | 0 | 0 | 0 | 0 | 0 | 0 | 0 | 0 | 0 | 1 |
| [288] | Implementing Telehealth in Pediatric Asthma. | 2020 | Perry et al. | 0 | 0 | 0 | 0 | 0 | 0 | 0 | 0 | 0 | 1 |
| [230] | The efficacy of a novel monitoring device on asthma control in children with asthma. | 2020 | Behrooz et al. | 0 | 0 | 0 | 0 | 0 | 0 | 0 | 1 | 0 | 0 |
| [290] | The Development and Preliminary Impact of CAMP Air: A Web-based Asthma Intervention to Improve Asthma Among Adolescents. | 2020 | Bruzzese et al. | 0 | 0 | 0 | 0 | 0 | 0 | 0 | 0 | 0 | 1 |
| [214] | Tailored Medication Adherence Incentives Using mHealth for Children With High-Risk Asthma (TAICAM): Protocol for a Randomized Controlled Trial. | 2020 | Henderson et al. | 0 | 0 | 0 | 0 | 0 | 0 | 0 | 1 | 0 | 0 |
| [356] | Impact of a self-monitoring application on pediatric asthma disparities. | 2020 | Nkoy et al. | 0 | 0 | 0 | 0 | 0 | 0 | 1 | 0 | 0 | 0 |
| [9] | Smart devices for the management of pediatric asthma: a scoping review protocol. | 2019 | Betz et al. | 0 | 0 | 0 | 0 | 0 | 0 | 0 | 1 | 0 | 0 |
| [194] | Evaluation of a web-based asthma self-management system: a randomised controlled pilot trial. | 2015 | Wiecha et al. | 0 | 0 | 0 | 0 | 0 | 0 | 0 | 1 | 0 | 1 |
| [306] | Impacts of online peer support for children with asthma and allergies: It just helps you every time you can't breathe well". | 2013 | Stewart et al. | 0 | 0 | 0 | 0 | 0 | 0 | 0 | 0 | 0 | 1 |
| [357] | Testing an intervention to promote children's adherence to asthma self-management. | 2007 | Burkhart et al. | 0 | 0 | 1 | 0 | 0 | 0 | 0 | 0 | 0 | 0 |
| [221] | Novel electronic adherence monitoring devices in children with asthma: a mixed-methods study. | 2020 | Makhecha et al. | 0 | 0 | 0 | 0 | 0 | 0 | 0 | 1 | 0 | 0 |
| [113] | Asthma education and monitoring: what has been shown to work. | 2008 | Brouwer et al. | 0 | 0 | 1 | 0 | 0 | 0 | 0 | 0 | 0 | 0 |
| [305] | Parent-reported outcomes of a shared decision-making portal in asthma: a practice-based RCT | 2015 | Fiks et al. | 0 | 0 | 0 | 0 | 0 | 0 | 0 | 0 | 0 | 1 |
| [228] | Adoption of a Portal for the Primary Care Management of Pediatric Asthma: A Mixed-Methods Implementation Study | 2016 | Fiks et al. | 0 | 0 | 0 | 0 | 0 | 0 | 0 | 1 | 0 | 1 |
| [358] | Asthma mobile applications: Are they ready for prime time? | 2018 | Blaiss et al. | 0 | 0 | 0 | 0 | 0 | 0 | 0 | 0 | 0 | 1 |
| [359] | Impact of online support for youth with asthma and allergies: pilot study. | 2012 | Letourneau et al. | 0 | 0 | 0 | 0 | 0 | 0 | 0 | 0 | 0 | 1 |
| [97] | Evaluation of home-monitoring of asthmatic children with the mini-Wright peak flow meter. | 1982 | Battu et al. | 0 | 0 | 1 | 0 | 0 | 0 | 0 | 0 | 0 | 0 |
| [88] | Peak flow monitoring for guided self-management in childhood asthma: a randomized controlled trial. | 2004 | Wensley et al. | 0 | 0 | 1 | 0 | 0 | 0 | 0 | 0 | 0 | 0 |
| [104] | Mobile phone technology in the management of asthma. | 2005 | Ryan et al. | 0 | 0 | 1 | 0 | 0 | 0 | 0 | 0 | 0 | 0 |
| [15] | A virtual asthma clinic for children: fewer routine outpatient visits, same asthma control. | 2017 | Van den Wijngaart et al. | 0 | 0 | 0 | 0 | 0 | 0 | 1 | 0 | 0 | 1 |
| [218] | Smartphones for Real-time Assessment of Adherence Behavior and Symptom Exacerbation for High-Risk Youth with Asthma: Pilot Study. | 2018 | Teufel et al. | 0 | 0 | 0 | 0 | 0 | 0 | 0 | 1 | 0 | 1 |
| [360] | Home telemonitoring and remote feedback between clinic visits for asthma. | 2016 | Kew et al. | 0 | 0 | 0 | 0 | 0 | 0 | 0 | 0 | 0 | 1 |
| [285] | What Is the Impact of Innovative Electronic Health Interventions in Improving Treatment Adherence in Asthma? The Pediatric Perspective. | 2019 | Licari et al. | 0 | 0 | 0 | 0 | 0 | 0 | 0 | 0 | 0 | 1 |
| [234] | Adherence feedback to improve asthma outcomes among inner-city children: a randomized trial. | 2009 | Otsuki et al. | 0 | 0 | 0 | 0 | 0 | 0 | 0 | 1 | 0 | 0 |
| [10] | Impact of eHealth on medication adherence among patients with asthma: A systematic review and meta-analysis | 2019 | Jeminiwa et al. | 0 | 0 | 0 | 0 | 0 | 0 | 0 | 1 | 0 | 1 |
| [302] | Internet-based home monitoring and education of children with asthma is comparable to ideal office-based care: results of a 1-year asthma in-home monitoring trial. | 2007 | Chan et al. | 0 | 0 | 0 | 0 | 0 | 0 | 0 | 0 | 0 | 1 |
| [238] | Effectiveness of Population Health Management Using the Propeller Health Asthma Platform: AÂ Randomized Clinical Trial. | 2016 | Merchant et al. | 0 | 0 | 0 | 0 | 0 | 0 | 0 | 1 | 0 | 1 |
| [205] | Enhancing medication adherence among inner-city children with asthma: results from pilot studies. | 2002 | Bartlett et al. | 0 | 0 | 0 | 0 | 0 | 0 | 0 | 1 | 0 | 0 |
| [96] | Clinical effect on uncontrolled asthma using a novel digital automated self-management solution: a physician-blinded randomised controlled crossover trial. | 2019 | Ljungberg et al. | 0 | 0 | 1 | 0 | 0 | 0 | 1 | 0 | 0 | 1 |
| [202] | Controller adherence following hospital discharge in high risk children: A pilot randomized trial of text message reminders. | 2019 | Kenyon et al. | 0 | 0 | 0 | 0 | 0 | 0 | 0 | 1 | 0 | 1 |
| [210] | Pragmatic trial of health care technologies to improve adherence to pediatric asthma treatment: a randomized clinical trial. | 2015 | Bender et al. | 0 | 0 | 0 | 0 | 0 | 0 | 0 | 1 | 0 | 0 |
| [114] | Improving pediatric asthma patient outcomes by incorporation of effective interventions. | 2001 | Marosi et al. | 0 | 0 | 1 | 0 | 0 | 0 | 0 | 0 | 0 | 0 |
| [100] | Using statistical process control charts for the continual improvement of asthma care. | 1999 | Boggs et al. | 0 | 0 | 1 | 0 | 0 | 0 | 0 | 0 | 0 | 0 |
| [294] | Pilot study of a randomized trial to evaluate a Web-based intervention targeting adolescents presenting to the emergency department with acute asthma. | 2018 | Joseph et al. | 0 | 0 | 0 | 0 | 0 | 0 | 0 | 0 | 0 | 1 |
| [361] | The value of FeNO measurement in asthma management: the motion for Yes, it's NO--or, the wrong end of the Stick! | 2008 | Bush et al. | 0 | 1 | 0 | 0 | 0 | 0 | 0 | 0 | 0 | 0 |
| [299] | Formative Evaluation for Implementation of a Low Literacy Pictorial Asthma Action Plan Delivered via Telehealth Improves Asthma Control. | 2020 | Vallabhan et al. | 0 | 0 | 0 | 0 | 0 | 0 | 0 | 0 | 0 | 1 |
| [362] | Novel methods for device and adherence monitoring in asthma. | 2018 | Bonini et al. | 0 | 0 | 0 | 0 | 0 | 0 | 0 | 1 | 0 | 0 |
| [300] | Mobile-based asthma action plans for adolescents | 2015 | Burbank et al. | 0 | 0 | 0 | 0 | 0 | 0 | 0 | 0 | 0 | 1 |
| [94] | Targeting quality of life in asthmatic children: The MyTEP pilot randomized trial. | 2019 | Montalbano et al. | 0 | 0 | 1 | 0 | 0 | 0 | 0 | 0 | 0 | 1 |
| [149] | Pulse oximeters to self monitor oxygen saturation levels as part of a personalised asthma action plan for people with asthma. | 2015 | Welsh et al. | 0 | 0 | 0 | 1 | 0 | 0 | 0 | 0 | 0 | 0 |
| [204] | The effect of an electronic monitoring device with audiovisual reminder function on adherence to inhaled corticosteroids and school attendance in children with asthma: a randomised controlled trial. | 2015 | Chan et al. | 0 | 0 | 0 | 0 | 0 | 0 | 0 | 1 | 0 | 0 |
| [216] | Health provider perspectives of electronic medication monitoring in outpatient asthma care: a qualitative investigation using the consolidated framework for implementation research. | 2020 | Kan et al. | 0 | 0 | 0 | 0 | 0 | 0 | 0 | 1 | 0 | 0 |
| [52] | Daily Telemonitoring of Exhaled Nitric Oxide and Symptoms in the Treatment of Childhood Asthma | 2009 | de Jongste et al. | 0 | 1 | 0 | 0 | 0 | 0 | 0 | 0 | 0 | 1 |
| [87] | How useful do parents find home peak flow monitoring for children with asthma? | 1992 | Lloyd et al. | 0 | 0 | 1 | 0 | 0 | 0 | 0 | 0 | 0 | 0 |
| [291] | Remote versus face-to-face check-ups for asthma. | 2016 | Kew et al. | 0 | 0 | 0 | 0 | 0 | 0 | 0 | 0 | 0 | 1 |
| [289] | Application of biomedical informatics to chronic pediatric diseases: a systematic review. | 2009 | Moeinedin et al. | 0 | 0 | 0 | 0 | 0 | 0 | 0 | 0 | 0 | 1 |
| [95] | Improving asthma outcomes and self-management behaviors of inner-city children: a randomized trial of the Health Buddy interactive device and an asthma diary. | 2002 | Guendelman et al. | 0 | 0 | 1 | 0 | 0 | 0 | 0 | 0 | 0 | 1 |
| [201] | The feasibility of text reminders to improve medication adherence in adolescents with asthma. | 2016 | Johnson et al. | 0 | 0 | 0 | 0 | 0 | 0 | 0 | 1 | 0 | 1 |
| [78] | Empowering pharmacists in asthma management through interactive SMS (EmPhAsIS): study protocol for a randomized controlled trial. | 2014 | De Vera et al. | 0 | 0 | 0 | 0 | 0 | 0 | 0 | 1 | 0 | 0 |
| [110] | Developing a Mobile Health Intervention for Low-Income, Urban Caregivers of Children with Asthma: A Pilot Study. | 2017 | Everhart et al. | 0 | 0 | 0 | 0 | 0 | 0 | 1 | 0 | 0 | 0 |
| [363] | Remote monitoring of asthma. | 2009 | Blanchet et al. | 0 | 0 | 0 | 0 | 0 | 0 | 0 | 0 | 0 | 1 |
| [292] | Computer game for inner-city children does not improve asthma outcomes. | 2003 | Huss et al. | 0 | 0 | 0 | 0 | 0 | 0 | 1 | 0 | 0 | 1 |
| [112] | Education, self-management and home peak flow monitoring in childhood asthma. | 2001 | Kamps et al. | 0 | 0 | 1 | 0 | 0 | 0 | 0 | 0 | 0 | 0 |
| [225] | Electronic Adherence Monitoring in a High-Utilizing Pediatric Asthma Cohort: A Feasibility Study. | 2016 | Kenyon et al. | 0 | 0 | 0 | 0 | 0 | 0 | 0 | 1 | 0 | 0 |
| [224] | Learnings from a pragmatic pilot trial of text messaging for high-risk adolescents with asthma. | 2018 | Dodds et al. | 0 | 0 | 0 | 0 | 0 | 0 | 0 | 1 | 0 | 1 |
| [102] | Home telemonitoring (forced expiratory volume in 1 s) in children with severe asthma does not reduce exacerbations. | 2012 | Deschildre et al. | 0 | 0 | 1 | 0 | 0 | 0 | 0 | 0 | 0 | 0 |
| [208] | The effects of combining Web-based eHealth with telephone nurse case management for pediatric asthma control: a randomized controlled trial. | 2012 | Gustafson et al. | 0 | 0 | 0 | 0 | 0 | 0 | 0 | 1 | 0 | 1 |
| [101] | Telemedicine offers new way to manage asthma. | 2001 | Murphy et al. | 0 | 0 | 1 | 0 | 0 | 0 | 0 | 1 | 0 | 0 |
| [89] | COVID-19: Technology-Supported Remote Assessment of Pediatric Asthma at Home. | 2020 | van der Kamp et al. | 0 | 0 | 1 | 0 | 0 | 0 | 0 | 1 | 0 | 1 |
| [364] | Technology-Based Interventions for Asthma-Can They Help Decrease Health Disparities? | 2016 | Baptist et al. | 0 | 0 | 0 | 0 | 0 | 0 | 0 | 0 | 0 | 1 |
| [365] | Using SMS to Provide Continuous Assessment and Improve Health Outcomes for Children with Asthma | 2012 | Yun et al. | 0 | 0 | 0 | 0 | 0 | 0 | 0 | 0 | 0 | 1 |
| [366] | Monitoring asthma with a Mini-Wright Peak Flow Meter. | 1985 | Plymat et al. | 0 | 0 | 1 | 0 | 0 | 0 | 0 | 0 | 0 | 0 |
| [11] | Digital asthma self-management interventions: A systematic review | 2014 | Morrison et al. | 0 | 0 | 0 | 0 | 0 | 0 | 0 | 0 | 0 | 1 |
| [367] | Asthma control and hospitalizations among inner-city children: results of a randomized trial. | 2004 | Guendelman et al. | 0 | 0 | 0 | 0 | 0 | 0 | 0 | 0 | 0 | 1 |
| [368] | Understanding the potential role of mobile phone-based monitoring on asthma self-management: qualitative study. | 2007 | Pinnock et al. | 0 | 0 | 1 | 0 | 0 | 0 | 0 | 0 | 0 | 1 |
| [308] | Telephone case management for asthma: an acceptable and effective intervention within a diverse pediatric population. | 2011 | Fisher-Owens et al. | 0 | 0 | 0 | 0 | 0 | 0 | 0 | 0 | 0 | 1 |
| [311] | A randomized controlled trial of an interactive voice response telephone system and specialist nurse support for childhood asthma management. | 2010 | Xu et al. | 0 | 0 | 0 | 0 | 0 | 0 | 0 | 0 | 0 | 1 |
| [237] | Effect of a mobile health, sensor-driven asthma management platform on asthma control | 2017 | Barrett et al. | 0 | 0 | 0 | 0 | 0 | 0 | 0 | 1 | 0 | 1 |
| [13] | Digital health interventions in children with asthma | 2020 | Ferrante et al. | 0 | 0 | 0 | 0 | 0 | 0 | 0 | 1 | 0 | 1 |
| [235] | Electronic monitoring devices as an intervention in Asthma: The story so far | 2018 | Adejumo et al. | 0 | 0 | 0 | 0 | 0 | 0 | 0 | 1 | 0 | 0 |
| [369] | The role of mobile apps in allergic respiratory diseases: An italian multicentre survey report | 2018 | Lombardi et al. | 0 | 0 | 0 | 0 | 0 | 0 | 0 | 0 | 0 | 1 |
| [252] | Adolescent feedback on predisposing, reinforcing and enabling features in asthma self-management apps | 2019 | Roberts et al. | 0 | 0 | 0 | 0 | 0 | 0 | 0 | 0 | 0 | 1 |
| [152] | Managing asthma and obesity related symptoms (Matadors): An mhealth intervention to facilitate symptom self-management among youth | 2020 | Nichols et al. | 0 | 0 | 0 | 0 | 0 | 0 | 1 | 0 | 0 | 1 |
| [370] | Screening for inhalation technique errors with electronic medication monitors | 2019 | Anderson et al. | 0 | 0 | 0 | 0 | 0 | 0 | 0 | 1 | 0 | 0 |
| [371] | Technology Interventions for Nonadherence: New Approaches to an Old Problem | 2018 | Bender et al. | 0 | 0 | 0 | 0 | 0 | 0 | 0 | 1 | 0 | 1 |
| [307] | Identifying an effective mobile health application for the self-management of allergic rhinitis and asthma in Australia | 2020 | Tan et al. | 0 | 0 | 0 | 0 | 0 | 0 | 0 | 0 | 0 | 1 |
| [372] | Evolution of Asthma Self-Management Programs in Adolescents: From the Crisis Plan to Facebook | 2016 | Liptzin et al. | 0 | 0 | 0 | 0 | 0 | 0 | 0 | 0 | 0 | 1 |
| [295] | Evidence-Based Review of Smartphone Versus Paper Asthma Action Plans on Asthma Control | 2019 | Murphy et al. | 0 | 0 | 0 | 0 | 0 | 0 | 0 | 0 | 0 | 1 |
| [373] | Short-term effect of a smart nebulizing device on adherence to inhaled corticosteroid therapy in asthma predictive index-positive wheezing children | 2018 | Zhou et al. | 0 | 0 | 0 | 0 | 0 | 0 | 0 | 1 | 0 | 1 |
| [374] | Monitoring and adherence in asthma management | 2015 | Szefler et al. | 0 | 0 | 0 | 0 | 0 | 0 | 0 | 1 | 0 | 0 |
| [217] | See I told you I was taking it! - Attitudes of adolescents with asthma towards a device monitoring their inhaler use: Implications for future design | 2017 | Howard et al. | 0 | 0 | 0 | 0 | 0 | 0 | 0 | 1 | 0 | 0 |
| [107] | Management of acute loss of asthma control in the yellow zone: A practice parameter | 2014 | Dinakar et al. | 0 | 0 | 1 | 0 | 0 | 0 |  | 0 | 0 | 0 |
| [375] | Adherence in childhood asthma: The elephant in the room | 2014 | Morton et al. | 0 | 0 | 0 | 0 | 0 | 0 | 0 | 1 | 0 | 0 |
| [165] | Telephone coaching for parents of children with asthma: Impact and lessons learned | 2010 | Garbutt et al. | 0 | 0 | 0 | 0 | 0 | 0 | 0 | 0 | 0 | 1 |
| [376] | Cost-effectiveness of a nurse-led telemonitoring intervention based on peak expiratory flow measurements in asthmatics: Results of a randomised controlled trial | 2007 | Willems et al. | 0 | 0 | 1 | 0 | 0 | 0 | 0 | 0 | 0 | 0 |
| [222] | Adherence in young children with asthma | 2006 | Graves et al. | 0 | 0 | 0 | 0 | 0 | 0 | 0 | 1 | 0 | 0 |
| [310] | The child asthma link line: A coalition-initiated, telephone-based, care coordination intervention for childhood asthma | 2010 | Coughey et al. | 0 | 0 | 0 | 0 | 0 | 0 | 0 | 0 | 0 | 1 |
| [232] | Electronic monitoring and feedback to improve adherence in pediatric asthma | 2012 | Spaulding et al. | 0 | 0 | 0 | 0 | 0 | 0 | 0 | 1 | 0 | 0 |
| [167] | Internet-based self-management compared with usual care in adolescents with asthma: A randomized controlled trial | 2012 | Rikkers-Mutsaerts et al. | 0 | 0 | 1 | 0 | 0 | 0 | 1 | 0 | 0 | 1 |
| [99] | Home Recording of Peak Expiratory Flow Rates and Perception of Asthma | 1985 | Sly et al. | 0 | 0 | 1 | 0 | 0 | 0 | 0 | 0 | 0 | 0 |
| [377] | Measuring adherence to asthma medication regimens | 1994 | Rand et al. | 0 | 0 | 0 | 0 | 0 | 0 | 0 | 1 | 0 | 0 |
| [108] | Improving patient outcomes with tools for asthma self-monitoring: A review of the literature | 2002 | Myers et al. | 0 | 0 | 1 | 0 | 0 | 0 | 0 | 1 | 0 | 0 |
| [103] | Asthma In-Home Monitoring (AIM) Trial | 2006 | NCT00282516 | 0 | 0 | 1 | 0 | 0 | 0 | 0 | 1 | 0 | 1 |
| [193] | Interventions on adherence to treatment in children with severe asthma: a systematic review . REF: | 2018 | Boutopoulou | 0 | 0 | 0 | 0 | 0 | 0 | 0 | 1 | 0 | 0 |
| [117] | Telemonitoring of Lung Function by Spirometry | 2020 | NCT04447664 | 0 | 0 | 1 | 0 | 0 | 0 | 0 | 0 | 0 | 1 |
| [162] | The virtual asthma clinic: description and analysis of website-use | 2016 | Van Den Wijngaart et al. | 0 | 0 | 0 | 0 | 0 | 0 | 1 | 0 | 0 | 1 |
| [197] | A mHealth Intervention to Improve Symptom Control in Children and Adolescents With Difficult-to-control Asthma | 2019 | NCT04166344 | 0 | 0 | 1 | 0 | 0 | 0 | 0 | 1 | 0 | 1 |
| [215] | Using Technology-Assisted Stepped Care Intervention to Improve Adherence in Adolescents With Asthma | 2019 | NCT04365556 | 0 | 0 | 0 | 0 | 0 | 0 | 0 | 1 | 0 | 0 |
| [239] | Using remote directly observed therapy (R-DOT) for optimising asthma therapy | 2017 | Shields et al. | 0 | 0 | 0 | 0 | 0 | 0 | 0 | 1 | 0 | 0 |
| [115] | Childhood Asthma Perception Study | 2016 | NCT02702687 | 0 | 0 | 1 | 0 | 0 | 0 | 0 | 0 | 0 | 0 |
| [116] | DragONE Study: acquisition and Maintenance of Paediatric Asthma Control: usual Care vs Innovative Devices | 2017 | NCT03273933 | 0 | 0 | 1 | 0 | 0 | 0 | 1 | 0 | 0 | 0 |
| [286] | AIM2ACT: a Mobile Health Tool to Help Adolescents Self-Manage Asthma (AIM2ACT) | 2020 | NCT04448002 | 0 | 0 | 0 | 0 | 0 | 0 | 1 | 0 | 0 | 1 |
| [223] | A prospective, randomized, controlled study of inhaler electronic monitoring devices to improve adherence in children with asthma | 2019 | Simoneau et al. | 0 | 0 | 0 | 0 | 0 | 0 | 0 | 1 | 0 | 0 |
| [195] | Pilot study of asthmawin mobile iphone app in the management of asthma | 2017 | Reece et al. | 0 | 0 | 1 | 0 | 0 | 0 | 0 | 1 | 0 | 1 |
| [301] | Internet-based self-management in adolescents with asthma: the role of education, monitoring and symptom perception | 2014 | Rikkers-Mutsaerts et al. | 0 | 0 | 0 | 0 | 0 | 0 | 0 | 0 | 0 | 1 |
| [206] | The effect of electronic monitoring combined with weekly feedback and reminders on adherence to inhaled corticosteroids in infants and younger children with asthma: a randomized controlled trial | 2020 | Chen et al. | 0 | 0 | 0 | 0 | 0 | 0 | 0 | 1 | 0 | 0 |
| [297] | Evaluation of a web-based asthma management intervention program for urban teenagers: reaching the hard to reach. | 2013 | Joseph et al. | 0 | 0 | 0 | 0 | 0 | 0 | 0 | 0 | 0 | 1 |
| [90] | Process evaluation of a nurse-led telemonitoring programme for patients with asthma. | 2007 | Willems et al. | 0 | 0 | 1 | 0 | 0 | 0 | 0 | 0 | 0 | 0 |
| [298] | A randomized controlled trial of a mobile application-assisted nurse-led model used to improve treatment outcomes in children with asthma. | 2019 | Lv et al. | 0 | 0 | 0 | 0 | 0 | 0 | 0 | 1 | 0 | 1 |
| [200] | Evaluation of a mobile health intervention to support asthma self-management and adherence in the pharmacy. | 2019 | Kosse et al. | 0 | 0 | 0 | 0 | 0 | 0 | 0 | 1 | 0 | 1 |
| [207] | Application of Human Augmentics: A Persuasive Asthma Inhaler. | 2017 | Grossman et al. | 0 | 0 | 0 | 0 | 0 | 0 | 0 | 1 | 0 | 1 |
| [194] | Evaluation of a web-based asthma self-management system: a randomised controlled pilot trial. | 2015 | Wiecha et al. | 0 | 0 | 0 | 1 | 0 | 0 | 0 | 1 | 0 | 1 |
| [198] | Asthma Management in the Era of Smart-Medicine: Devices, Gadgets, Apps and Telemedicine. | 2018 | Katwa et al. | 0 | 0 | 0 | 1 | 0 | 0 | 0 | 1 | 1 | 1 |
| [211] | Controller Medication Refill Rates in Underserved Pediatric Asthma Patients After Use of a Smartphone Application | 2019 | Sabhae Gangadharappa et al. | 0 | 0 | 0 | 0 | 0 | 0 | 0 | 1 | 0 | 1 |
| [93] | Smartphone-based vs paper-based asthma action plans for adolescents. | 2017 | Perry et al. | 0 | 0 | 1 | 0 | 0 | 0 | 0 | 0 | 0 | 1 |
| [233] | Sensor-Based Electronic Monitoring for Asthma: A Randomized Controlled Trial | 2021 | Gupta et al. | 0 | 0 | 0 | 0 | 0 | 0 | 0 | 1 | 0 | 0 |
| [378] | Exciting Era of Sensor-Based Electronic Monitoring of Adherence in Pediatric Asthma | 2021 | Ramsey et al. | 0 | 0 | 0 | 0 | 0 | 0 | 0 | 1 | 0 | 0 |
| [163] | Ambulatory Management of Childhood Asthma Using a Novel Self-management Application. | 2019 | Nkoy et al. | 0 | 0 | 0 | 0 | 0 | 0 | 1 | 0 | 0 | 1 |
| [199] | Acceptability of an interactive asthma management mobile health application for children and adolescents. | 2015 | Farooqui et al. | 0 | 0 | 0 | 0 | 0 | 0 | 0 | 1 | 0 | 1 |
| [91] | I have most of my asthma under control and I know how my asthma acts: Users' perceptions of asthma self-management mobile app tailored for adolescents. | 2020 | Schneider et al. | 0 | 0 | 1 | 0 | 0 | 0 | 0 | 0 | 0 | 1 |
| [12] | A review of the use and effectiveness of digital health technologies in patients with asthma | 2018 | Unni et al. | 0 | 0 | 0 | 0 | 0 | 0 | 0 | 0 | 0 | 1 |
| [92] | An Internet-based store-and-forward video home telehealth system for improving asthma outcomes in children | 2003 | Chan et al. | 0 | 0 | 1 | 0 | 0 | 0 | 0 | 0 | 0 | 1 |
