# Supplementary material for: eHealth Technologies for Monitoring Pediatric Asthma at Home: Scoping Review
Source: J Med Internet Res. 2023 Jul 21;25:e45896. doi: 10.2196/45896 (PMC10403763; doi:10.2196/45896)
Supplement: Multimedia Appendix 3 [file jmir_v25i1e45896_app3.docx]

**Multimedia Appendix 3.** Domain-specific discussion and future perspectives.

***Domain air quality***
Childhood asthma is characterized by airway hyperresponsiveness and commonly associated with allergy [384]. Increased exposure to allergens, air pollution or unfavorable weather conditions can trigger asthma exacerbations [5,385-387] and monitoring air quality can help to alarm patients that they are more prone to an exacerbation, even in the absence of symptoms [5]. This could potentially help to remind patients not to forget, or possibly even increase, their medication. Furthermore, it could help patients to identify personal triggers or identify areas with increased exposure that they might be able to avoid [388,389]. This could support patient self-management of asthma symptoms.
We identified several measurement methods: bedroom measurements, measurements with a wearable monitor and adjusted measurements from weather stations. Since people in western life-style areas spend approximately 80% of their time indoors, indoor concentrations represent an important determinant of personal exposure and this makes bedroom or wearable measurements the preferred option to assess individual exposure [333]. Although monitoring air quality is available and seems a valuable monitoring tool, the added value to other asthma monitoring tools and the application of air quality monitoring in (multi-domain) asthma interventions has yet to be determined.

***Domain airway inflammation markers***
Fractional exhaled nitric oxide (FeNO) is thought to reflect the presence of eosinophilic airway inflammation, and is an easy, non-invasive test that has held promise in providing additional objective data. It has however not shown to be of additional value in home-based pediatric asthma management [5,390]. This is reflected in the limited number of studies that investigated FeNo home-monitoring. They mostly focused on the feasibility, validity and repeatability of FeNo measurements at home, and despite the technical possibilities to measure FeNo at home, the lack of added value for pediatric asthma management has not led to many intervention studies using FeNo.

***Domain lung function***
Lung function monitoring has been a monitoring domain from the start of asthma home-monitoring, as changes in lung function directly reflect airway physiology and are therefore related to asthma manifestations. Lung function self-monitoring is included in current guidelines, especially in patients who are known to be poor symptom perceivers [5]. This review showed that 1) home-monitoring lung function devices are accurate [59,73,353], 2) lung function measurements can be performed reliably at home [73,78] with the right instructions [76,78,102], quality control systems [73,78] and/or gamification approaches to enhance spirometry quality [75,76] and 3) lung function should be monitored electronically to prevent reporting bias [67,69,70,212]. Moreover, caution is needed with regards to the interpretation of long term home-spirometry monitoring, as a considerable degree of lung function variation in children with asthma has been shown, even when asthma is clinically stable [113]. This, in combination with the aforementioned conditions, may explain the limited correlation of home-measured lung function parameters (especially PEF [64,66,83-85]) with asthma disease activity [64,66,83-85] and varying asthma outcomes of lung function monitoring intervention studies. Regular monitoring on standard intervals may not be the most effective monitoring protocol and it can be suggested to perform additional measurements when experiencing symptoms or measuring reversibility at home may be more effective, as this information may especially help patients (or healthcare providers) to timely intervene during a starting exacerbation [2]. Only few studies within this review performed these type of lung function measurements at home [87-90], indicating a scope for future intervention studies.

***Domain activity***
Physical activity (PA) is paramount for a healthy life in children and is also closely related to asthma, as recurrent (exercise induced) asthmatic symptoms may withhold children from fully participating in physical activity. Throughout literature different results are reported with regards to whether PA levels are different in symptomatic asthmatics [391-394]. A similar diversity in results was found in the validation studies in this review [120,121,146], which may be explained by the heterogeneity of methods used to assess PA and asthma. Asthma guidelines therefore do not focus on regular physical activity monitoring, explaining no intervention studies in this domain. However, guidelines do recognize its important health benefits and recommend to encourage asthmatics to engage in PA [5], which may explain why the use of accelerometry monitoring is reflected within multi-domain asthma eHealth tools [33,34,118]. Moreover, it may play a role in personalized trigger identification by combining PA monitoring with symptoms [24,31,118] and/or air quality monitoring [23]. Future research may therefore focus on how PA monitoring could be applied to benefit asthma treatment. Moreover, systematic evaluation of the relation between asthma and PA could be performed, while taking the heterogeneity of methods and asthma definitions in consideration, therefore providing detailed recommendations for guideline-makers.

***Domain sleep***
Nighttime symptoms or awakenings are common symptoms of childhood asthma and disturbed sleep is associated with behavioral and developmental difficulties [395,396]. Nocturnal symptoms or earlier wake-up time could indicate loss of asthma control and identification and control of nocturnal symptoms is therefore essential [395]. The available non-obtrusive monitoring options allow an objective measurement of sleep duration and quality [120]. This is helpful, as it difficult to assess sleep by a questionnaire (only a moderate correlation was shown between the two) [120]. Although sleep seems a relatively easy (nowadays even most smartwatches can measure sleep duration and give an indication on sleep quality) and potentially meaningful measurement, sleep monitoring has not yet been adopted into intervention studies. A possible explanation might be the many other factors besides asthma that influence sleep duration and sleep quality [397]. Further research is could therefore explore and validate which objectively home-measured sleep parameters do strongly relate to asthma manifestations and how these are influenced by other aspects such as age, sleep behavior, chronic rhinitis.

***Domain audiovisual***
Every pediatrician is trained to use auscultation to characterize lung-sounds during physical examination. An asthma exacerbation leads to bronchus obstruction, which can result in wheezing, prolonged expiration and increased cough [5]. As these sounds are closely related to asthma symptoms and auscultation is common asthma care practice, monitoring them at home could be beneficial in children with asthma. Different methods of sound monitoring were found, using microphones placed in the room [22], on the body [125-130] or in a digital stethoscope [131,132]. All methods recognized the importance of acquiring a clean signal by limiting ambient noise and applying the right filtering techniques to obtain good sensitivity and specificity. This would make sound monitoring as close to the body the best way to obtain a strong signal, however this is also the most obtrusive when performing longer measurements.

To date, approaches involving the characterization of asthma sounds seem insufficient for detailed diagnostics, for example to discriminate healthy from asthmatic coughs [125-127]. However, approaches for counting coughs and wheeze seem applicable in a home-setting and may provide relevant information correlating with exacerbations [55] and lung function [58,176]. We found no studies regarding video-based symptom monitoring at home, but Freeman et al. (2017) combined sound and video to triage asthma symptom severity in the ED [327]. Interestingly, most studies in the audiovisual domain focused on primary school children (74%), which may be explained by the fact that respiratory sounds and visual signs of respiratory distress (such as nasal flaring, retractions etc.) are more prominent at a young age [398]. An interesting follow-up question would thus be to investigate which population benefits most from audiovisual monitoring at home and to investigate the added value of visual monitoring compared to audio-monitoring alone.

***Domain other physiological measurements***Measuring other physiological parameters such as heart rate, respiratory rate, pulsoximetry and EMG may provide insights in the level of respiratory distress and therefore may enable to catch asthma exacerbations or first symptoms of deterioration. Limited work has been found in this review that investigated the home-based application of these measurements. Interesting is that most of the studies focused on young children, which may be explained by the fact that young children have less respiratory reserves to compensate for obstruction with tidal volume (variation) compared to adolescents and adults, leading to earlier notable signs of respiratory distress (high respiratory rate & heart rate, high diaphragm activity and in case of insufficient oxygenation a decrease in pulse oximetry) [398]. Applications for monitoring these physiological parameters seem feasible on either symptomatic periods [144-146], nighttime [3,123] or after exercise provocation [3]. However, further research is needed to validate these different monitoring applications within eHealth intervention on their effects on asthma outcomes.

***Domain questionnaires***
There are several questionnaires that are regularly used to evaluate asthma control, such as the (Childhood) Asthma Control Test or the Asthma Control Questionnaire [5,399-401]. These questionnaires evaluate asthma symptoms over the past week(s). They used to be administered on paper, but nowadays questionnaires are often administered electronically. This seems an improvement, as a higher concordance with symptoms was reported and patients preferred electronic over paper questionnaires [155,156].

Electronic questionnaires enable health care professionals to telemonitor symptoms between visits. This facilitates ongoing monitoring of asthma control and proactive medical decision-making when symptoms increase. Questionnaires are an easy-to-use, low-cost and widely available tool to monitor symptoms. A disadvantage however, is the poor relation between questionnaires and objective measurements of airflow limitation [402,403]. This might be due to the limited ability of some patients to accurately perceive symptoms of airflow limitation [181,404]. Hyper perceivers will report substantial discomfort in the face of minimal bronchoconstriction and poor perceivers will report no symptoms even in the presence of severe obstruction. The use of objective measures of airflow limitation would be beneficial in these groups because they are otherwise prone to overtreatment (hyper perceivers) or undertreatment and exacerbations (poor perceivers) [404]. Future work could thus be focusing the identification of patient groups that can be adequately monitored with online questionnaires (good perception) and patients groups that would require additional monitoring (poor perception) in order to achieve good asthma control.

***Domain medication monitoring***
Adherence to medication has been identified as an essential component of asthma management [8] and nonadherence rates are alarmingly high [28,168,405] and reported adherence rates largely vary between studies [375] Electronic medication monitoring can be a usable tool to identify patients with suboptimal adherence who need optimization of adherence instead of a medication step up [343]. Although extensively applied in research settings, application of electronic monitoring in daily clinical care is lacking [374]. Electronic medication monitoring devices can also function as an aid to remind patients to take medication or as a tool to motivate patients to stay compliant [179]. Monitoring of rescue medication use can help to determine the degree of symptom severity and to identify personal triggers that require rescue use. EMD’s accurately reflect real-life medication use, in contrast to pharmacy refill rates which only reflect the amount of collected medication, or diaries which are prone to recall bias and incorrect reporting. EMD’s are an easy-to-use and helpful tool. Its use can be especially beneficial in subgroups that are at a higher risk of non-adherence and thus require special attention and monitoring, such as adolescents [168] and minorities [168,169].

Besides medication adherence, a good inhalation technique is also essential to achieve optimal therapy effects. Although it is difficult to automatically measure inhaler technique, there are devices on the market that allow measurement of essential steps in taking inhaler medication, such as shaking the canister, correct positioning and inhalation force [196]. They are however not adopted into general practice. Video capture of inhaler technique does not require special equipment and can be used with any type of inhaler [92,229,239,240] but will not monitor every medication usage and is time-consuming to rewatch. A combined automated measurement of adherence and technique would therefore be preferable and could guide clinicians in optimizing medication regimens.

Most studies focusing on medication monitoring showed a positive effect on adherence, especially when combined with reminders. However, limited studies focused on other asthma outcomes or sustained effects. An interesting follow-up research questions could thus be ‘What are the long-term effects of medication monitoring on adherence and asthma outcomes?’. Moreover, when considering that certain subgroups are at higher risk of non-adherence and certain methods (such as reminders) resulted in better effects it may of interest to investigate: ‘What are the best personalized strategies to provide feedback based on medication monitoring data to optimize adherence?’

***Digital environment***
It is remarkable to notice that only a very small amount of children (3%) with asthma had ever used an app directly related to their asthma, while apps for other chronic diseases have been able to attract and retain users to a much greater degree [248]. Multiple examples of digital tools containing a variety of functionalities (such as diaries, education, digital action plan, medication reminder, HCP-communication) have been found, but do not effectively reach the target group, which may be due to clinical asthma guidelines who do not incorporate such tools yet [5]. Moreover, most tools have not been designed to address barriers faced by racial/ethnic minority groups or those with low socio-economic status or low health literacy [13]. This emphasizes the importance of patient-centered design approaches and personalized long-term adherence strategies, such as rewarding or gamification [406].

This review furthermore showed a high amount of phone based application (73%), which is comparable to other reviews [406]. A potential advantage of apps over other technological interventions is that children, especially adolescents, almost always carry their smartphones, which allows to capture real-life data in symptomatic events. Most interventions were furthermore nurse-led as asthma nurses have a valuable role in monitoring asthma progression and addressing asthma management pitfalls. eHealth interventions provide them the tools to monitor in an efficient way and consult the pediatrician when needed, provide help to patients when needed and build-up self-management [407]

Although study results on effects of digital asthma tools are heterogeneous, it can be concluded that most studies reported beneficial asthma outcomes and no negative effects were found, emphasizing the low risks of remote digital interventions. This has also been reported in other reviews about eHealth asthma interventions [406]. Also within the broader range of eHealth applications in chronic pediatric diseases mixed results between superiority and non-inferiority were reported [10,408-411]. Although these reviews included risk of bias analyses, some restraints will remain for potential publication bias. Eysenbach [67] suggested a specific eHealth registry for eHealth studies to reduce publication bias and to prevent selective reporting of positive outcomes. Moreover, eHealth specific CONSORT criteria were created, for example including the reporting of harms or unintended effects and the reporting of ancillary analyses in order to distinguish pre-specified from exploratory analyses [68]. The current trend of publishing eHealth protocols could also help to facilitate transparency [69].

Moreover, a recurrent pattern in the intervention results is that eHealth studies which target their intervention on children with uncontrolled asthma at baseline, seem to report better outcomes on symptom improvement [189,211,297] and medication adherence [228]. Therefore, suggesting that the careful selection of the target population for eHealth care is needed to find effectiveness for digital tools. Moreover, regular use of the tool [96] and the communication technology used [231] could benefit the outcomes. Future research could therefore focus on how to personalize eHealth strategies for pediatric asthma to maximize the effect on health outcomes, while obtaining long-term intervention adherence and reaching the complete target group.
